# Accounting for Differences in Plasma Protein Binding between Fish and Humans for Supporting Environmental Risk Assessment of Pharmaceuticals Using the Fish Plasma Model

**DOI:** 10.1021/acs.est.5c15513

**Published:** 2026-04-21

**Authors:** A. Ross Brown, Maciej Trznadel, Siffreya Pedersen, Alison Nimrod Perkins, Michael Lee, Michael Mohutsky, Alec Bell, Tea LM Pihlaja, Tiina Sikanen, Mirco Weil, Anja Coors, Rod W. Wilson, Charles R. Tyler

**Affiliations:** † Biosciences, Faculty of Health and Life Sciences, 3286 University of Exeter, Stocker Road, Exeter, Devon EX4 4QD, United Kingdom; ‡ 1539 Eli Lilly and Company, Indianapolis, Indiana 46285, United States; § Faculty of Pharmacy, 124452 University of Helsinki, PL 56, Viikinkaari 5, Helsinki 00014, Finland; ∥ ECT Oekotoxikologie GmbH, Boettgerstr. 2-14, Floersheim/Main 65439, Germany

**Keywords:** albumin, AGP, apolipoproteins, blood plasma, cyprinid fish, salmonid fish, ionizable pharmaceuticals

## Abstract

The Fish Plasma Model (FPM) predicts steady-state concentrations of active pharmaceutical ingredients (APIs) in fish blood plasma based on partitioning from the surrounding (water) environment. Total plasma concentration is then compared to the human therapeutic concentration to indicate environmental risk, assuming that human pharmaceutical targets are conserved in fish. However, only the unbound fraction (f_u_) of API is available for pharmacological action and plasma protein binding, and the binding of APIs may differ between species. We quantify f_u_ for 44 APIs with wide ranging physicochemical properties in three fish species: a salmonid, rainbow trout (*Oncorhynchus mykiss* *)* and two cyprinids, fathead minnow (*Pimephales promelas*), and koi carp (*Cyprinus rubrofuscus*), and draw comparisons with f_u_ in humans. We examine interspecies differences in f_u_ in relation to blood physicochemistry and protein and lipid composition. We show that anionic APIs often exhibit substantially (×10) higher f_u_ in fish compared with humans, and this was most apparent in rainbow trout, despite this species possessing protein(s) orthologous to human serum albumin, a major binding protein for anionic APIs in humans. We recommend accounting for f_u_ in fish versus humans and using rainbow trout as a conservative species in the FPM for modeling API availability and effects in fish.

## Graphical abstract

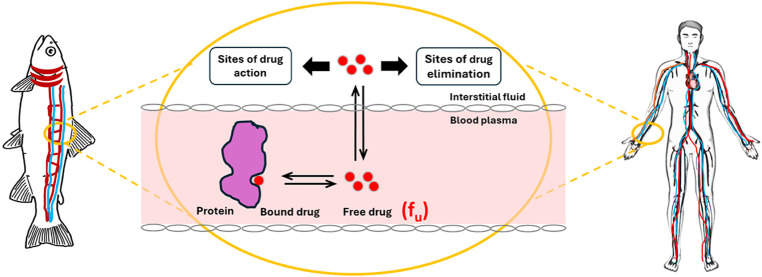

## 1 Introduction

Fish display high levels of conservation of human drug targets,^1–3^ and they are exposed to a wide range of active pharmaceutical ingredients (APIs) found in surface waters globally,^4^ indicating potential environmental risk. In addition to having comparable target binding site(s), risks of APIs to fish depend on their bioavailability (relative uptake from the water and/or diet), how they are distributed in the body tissues, the ability of fish to metabolize them, and the manner in which APIs are bound and transported around the body in the blood plasma. Predicting API risks in fish, therefore, can be better assessed by understanding how internal tissue concentrations compare with environmental exposure concentrations. In such analyses, factors including an organism’s anatomy and physiology and the API’s physicochemical properties (e.g., hydrophobicity and ionization state)^5,6^ may all affect how an API's concentration in the blood compares with the environmental water concentration.

Uptake of APIs from water into fish occurs principally via the gills into the bloodstream, and, assuming continuous aqueous exposure, the steady-state blood plasma concentration in fish (F_ss_PC) can be predicted for APIs using the Fish Plasma Model (FPM) based on their environmental water concentration (W) and hydrophobic partitioning (P_Blood:Water_) (eq 1).^5–7^ For the majority of APIs, which are ionizable, partitioning is best quantified using the pH-dependent distribution coefficient Log D_ow_ (P_Blood:Water_ = 10^(0.73 × Log Dow – 0.88)^).^8^

1 $$F_{\mathrm{ss}}{\mathrm{PC}=W\times P}_{\mathrm{Blood:Water}}$$

The threshold concentration of an API in water presenting a potential risk to fish (i.e., therapeutic water concentration, TWC), can also be predicted based on partitioning and the human therapeutic plasma concentration (*C*_max_) known to elicit pharmacological effects in humans (eq 2)­

2 $${\mathrm{TWC}=C}_{\max}{/P}_{\mathrm{blood:water}}$$

Key assumptions for this risk prediction are that *C*_max_ can be “read-across” from humans to fish and that adverse “off-target” effects (e.g., on survival, growth, and reproduction) will occur at higher plasma concentrations than pharmacological effects.^6,7,9,10^ Predicted steady-state plasma concentrations in fish have been shown for a range of APIs (largely cationic or neutral APIs) to meet or exceed measured plasma concentrations,^11^ providing an accurate or conservative assessment of exposure risk, respectively. However, plasma concentrations for anionic APIs, including nonsteroidal anti-inflammatory drugs (NSAIDs) such as ibuprofen, may be underpredicted by up to 2 orders of magnitude in the FPM.^12,13^ Furthermore, adverse effects of NSAIDs in fish have been shown to occur across a range of plasma concentrations, both above and below human therapeutic concentrations.^14^ API uptake from the surrounding water, concentration in the bloodstream, and potency to elicit effects in fish are likely to be affected by physiological factors that are not currently accounted for in the FPM, including metabolism and blood plasma protein binding of APIs in fish.

Plasma protein binding has a strong influence on API potency and efficacy.^15^ Indeed, according to the “free drug hypothesis”, only the unbound fraction (f_u_) of API in circulating blood plasma (not the total concentration in plasma, often represented by *C*_max_) is available for pharmacological action and for metabolism and excretion.^16,17^ Importantly, there may be substantial differences in f_u_ between fish and humans, which in turn could have a significant bearing on an API’s comparative potency. For example, according to limited data, f_u_ values for neutral and positively charged (cationic) APIs appear to be similar in rainbow trout and humans, while for negatively charged (anionic) APIs, such as ibuprofen, f_u_ is 15× higher in rainbow trout compared with humans. For another NSAID, naproxen, f_u_ is reported to be more than 80× higher in trout.^18^ Accounting for the higher unbound fractions of these NSAIDs in fish is likely to be especially important when assessing API fate and effects in fish.

Important outstanding questions when comparing API potencies in fish versus humans include whether comparable plasma proteins exist in fish, if they have similar capacities for binding APIs, and whether contrasting physiological conditions, such as blood pH and temperature, may also contribute to interspecies variations in f_u_.^18,19^ Given the high diversity among fish species (>37,000 extant species),^20^ establishing differences in plasma protein binding among fish species, and between fish and humans, is important for assessing the availability and environmental effects of APIs.

Plasma protein binding of APIs in humans is largely attributed to human serum albumin (HSA), which constitutes ∼55% of the total plasma protein mass, and alpha(1)-acid glycoprotein (AGP), which constitutes 3–5%.^21,22^ HSA has a molecular weight of 66 kDa, and there are three major binding sites for APIs: Sudlow drug site DS1 (with lysine residues) preferentially binds bulky, heterocyclic, anionic/acidic APIs such as warfarin; DS2 (bearing lysine, arginine, and serine residues) preferentially binds aromatic APIs such as anionic/acidic ibuprofen and cationic/basic APIs including diazepam^23^ and propranolol;^24^ DS3 (bearing multiple amino acid residues) binds a diverse assortment of acidic, neutral, and basic molecules.^25^ AGP has a molecular weight of 41–44 kDa, including five glycan chains bearing negatively charged sialic acid groups, which surround two high affinity binding sites for basic/cationic APIs.^26^ AGP also possesses one binding site for acidic/anionic APIs^27^ and up to seven sites for steroids.^28^

Albumin is conserved across mammalian species, and AGP is present in the majority of mammals.^29^ *In silico, in vitro,* and *in vivo* studies in humans and other mammals have shown that albumin, AGP, and numerous apolipoproteins (associated with HDL, LDL, VLDL cholesterol, and chylomicrons) can bind, store, and transport a wide variety of endogenous and exogenous ligands, including APIs.^21,26,30^ Other plasma proteins shown to play more limited roles in binding APIs in humans include fibrinogen (binding betablockers),^31^ C-reactive protein (binding statins),^32^ and transferrin (binding antineoplastics)^33^ (see summary in Supporting Information Table 1). Orthologous proteins to HSA have been shown, by genome sequencing, to be lacking in most fish (e.g., cyprinids, including carps and minnows), but HSA orthologues do occur in some fish species, which are phylogenetically closer to humans (e.g., salmonids, including trout).^30,34^ AGP also appears to be absent in most fish, apart from a few notable exceptions, again including trout.^35^

Here, we first conducted an analysis of published articles and databases concerning plasma protein composition and API binding potential in different fish species. We then performed a series of *in vitro* assays to quantify plasma proteins and plasma protein binding for a selection of 44 APIs, with wide-ranging physicochemical properties, in humans and select fish species, or genera, commonly used for pharmaceutical environmental risk assessment (ERA): rainbow trout (*Oncorhynchus mykiss*), koi carp (*Cyprinus rubrofuscus*), and fathead minnow (*Pimephales promelas*). The hypotheses we tested are (i) anionic APIs exhibit higher f_u_ in fish than in humans; (ii) f_u_ is higher in fish that lack albumin and AGP (i.e., koi carp and fathead minnow) compared with fish (i.e., rainbow trout) that possess albumin and AGP orthologues.^18^ Interspecies differences in f_u_ are anticipated to be greater for APIs that are highly bound in humans. The implications of our results for *in silico* modeling, including the Fish Plasma Model, were then explored with a view to (re)­prioritizing APIs for *in vivo* assessments of API availability and effects in fish.

## 2 Materials and Methods

### 2.1 Evaluation of Published Articles Characterizing Plasma Proteins in Fish

The capacity for plasma proteins to bind an API depends on various factors, including (i) the structural and functional properties of plasma proteins (e.g., charge, specificity, and accessibility of API binding sites)^21,36^ and (ii) the physicochemical properties of the API.^21,37^ In humans, APIs with low lipophilicity (Log K_ow_ < 3), low molecular weight (MW < 400), and high levels of ionization exhibit low plasma protein binding (f_u_ ≥ 10%).^37^ In the first instance, we reviewed publications concerning plasma protein composition in fish based on genome sequencing and annotation^30,34^ and plasma protein molecular properties and binding potentials in fish to establish commonalities and differences with those in humans.^38,39^

### 2.2 Evaluation of Current Genomic Data to Assess the Conservation of Plasma Proteins in Fish Compared with Humans

OrthoFinder (v3.0, https://github.com/davidemms/OrthoFinder)^40^ was used to search available databases for the most recently available data on orthologues of genes that encode albuminoid proteins, AGP, or any apolipoprotein. Selected species included a range of ray-finned, teleost fish, including cyprinids and salmonids, as well as various tetrapods, including humans, for comparison (Supporting Information Table 2). The ghostshark (*Callorhinchus milii*) was included to root the species tree. Proteomes for all species, with the exception of the fathead minnow, were downloaded from the Ensembl database,^41^ taking the “pep.all” files. The fathead minnow is not currently included in the Ensembl database, and therefore, the proteome for this species was downloaded from NCBI’s RefSeq database (RefSeq Release 230).^42^ The longest transcript per gene of the Ensembl data sets was selected using the primary_transcript.py script supplied with OrthoFinder, and the program was run using default parameters.

For the selection of orthogroups that represented the binding proteins of interest, BioMart (https://www.ensembl.org/info/data/biomart/index.html) was used to create 4 data sets that included all genes from human, zebrafish, common carp, and rainbow trout. These included the gene identifiers of any orthologues identified by Ensembl among the three fish species. These data sets were filtered to only include reference apolipoproteins, AGP, or albuminoids in the gene name, description, or symbol. A list of all identifiers of proteins defined as apolipoproteins of the fathead minnow was generated from NCBI’s RefSeq database.^42^ The resulting list of all gene IDs was used to search the results from OrthoFinder.^40^

### 2.3 Evaluation of Published Data on Plasma Protein Binding of APIs in Fish Compared with Humans

Existing data quantifying plasma protein binding of APIs in fish were retrieved from the Phish-Pharm database^43^ and supplemented by additional data retrieved from the available published literature. Metadata regarding study design (e.g., *in vitro, in vivo*), fish (e.g., fish species size, age, water and/or blood temperature, and blood pH), and APIs (e.g., acid or base, ionized or unionized, LogD_ow_ (pH), MW) were also captured alongside plasma protein binding data to enable identification of key factors associated with unbound fractions of API (f_u_).

Published human plasma protein binding data were gathered.^44–46^ For each API, the fraction unbound (f_u_) for each fish species was divided by f_u_ for humans to give the relative fraction unbound (Rf_u_):

3 $$Relative\;fraction\;unbound\left(Rf_{u}\right){=f}_{u}\mathrm{\_fish}/f_{u}\mathrm{\_human}$$

### 2.4 Analysis of Blood and Blood Plasma Composition in Selected Model Fish Species and in Humans

Human blood plasma was obtained from a disease-free cohort (*n* = 6 individuals) of mixed sex/age to account for sex-related variation in the plasma proteome.^47^ The pooled blood plasma was supplied by BioIVT (Westbury, NY) and stored at −80 °C for up to 3 months before use. Use of the commercially sourced human blood plasma samples was approved by the University of Exeter’s Research Ethics Committee: Reference 520764. Healthy fish, including *n* = 24 adult male and female fathead minnows (∼7 and ∼2 g in body (wet) weight, respectively), *n* = 20 subadult male and female koi carp (∼100 g wet weight), and *n* = 6 subadult, triploid female rainbow trout (∼300 g wet weight), were sourced from commercial suppliers, and prior to blood sampling, they were held on maintenance diets in the laboratory for one to two weeks at recommended species- or genus- specific water temperatures^48^ in flowthrough water conditions (>6 L per gram of fish per day),^49^ under low light, with minimal disturbance, and gentle water aeration (maintaining >80% dissolved oxygen, < 1 μg ammonia/L, and pH ∼ 7.8–8.0). Fish were not fed for 24 h before sampling to aid sedation and avoid postprandial alteration of blood chemistry.^50^ Furthermore, to avoid startle-induced metabolic acid, lactate release, and changes in blood pH during sampling,^51^ fish were sedated by slowly introducing nonacidifying anesthetic (75 mg/L benzocaine)^52^ into the fish holding tanks (which were covered the night before with opaque material to minimize visual disturbance). After confirming sedation using audible and tactile stimulation, fish were transferred to a gill irrigation chamber, allowing artificial ventilation of the gills with the same water chemistry as the fish holding tanks and containing 30 mg/L benzocaine to maintain sedation.^52^ Blood was sampled from each fish (1000 μL for rainbow trout, 5000 μL for koi carp, 20–100 μL for fathead minnow) via the caudal vasculature using a syringe and hypodermic needle (23 gauge for rainbow trout and koi carp, 30 gauge for fathead minnow). Before blood sampling, each syringe used for trout and fathead minnow was primed by drawing and emptying a solution of 5000 IU/mL heparin to prevent blood clotting.^50^ Syringes used for carp were coated with 1 mg/mL of the K3EDTA anticoagulant. The use of fish for blood sampling was approved by the University of Exeter’s Animal Welfare and Ethical Review Body (Study Request 0085). The blood sampling procedure was performed according to Protocol 1, Step 5 of Project License PP5718563. Fish were subsequently killed according to Schedule 1 of the Animal (Scientific Procedures) Act.^53^

Blood pH (±0.01 pH units) was measured in a 30–50 μL subsample of blood using a calibrated pH meter (Hanna Instruments, Leighton Buzzard, UK) with a clean micro-pH electrode (Metrohm, Biotrode, UK) held in a water bath at the same temperature as the fish holding tanks. Hematocrit (proportion of red blood cells to plasma volume) was determined for rainbow trout after drawing blood into a heparinized capillary tube, which was then capped and spun in a microcentrifuge (Thermo Fisher Scientific, Waltham, MA, USA) at 7000 rpm for 4 min. The remaining blood was centrifuged in aliquots of up to 1 mL at 10,000*g* at 4 °C for 2 min. Supernatant plasma was then transferred in 100–500 μL aliquots into separate microcentrifuge tubes for storage at −80 °C, prior to thawing and assessment of plasma protein binding of APIs (Section 2.5) and plasma protein and lipid composition in each test species (as follows).

Total plasma protein concentration was measured in triplicate, in pooled plasma samples (diluted 1:100 in RO water), using the Pierce BCA Protein Assay Kit, according to the manufacturer’s instructions.^54^ The BCA assay relies on the formation of a purple complex of bicinchoninic acid with Cu^+^ cations after the reduction of Cu^2+^ by protein (via the biuret reaction).^55^ A NanoQuant Infinite M200 Pro UV–visible light spectrophotometer (Tecan, Männedorf, Switzerland) or a Varioskan LUX microplate reader (Thermo Fisher Scientific, Waltham, MA, USA) was used to quantify total protein concentration based on the intensity of light absorbance by the purple complex at 562 nm, calibrated against a series of Bovine Serum Albumin (BSA) standards (0 to 2 mg/mL).

Albumin concentration was measured in triplicate, in pooled plasma samples (diluted 1:1 or 1:2 in RO water), using the bromocresol purple (BCP) assay kit, according to the manufacturer’s instructions.^56^ BCP forms a yellow-colored complex specifically with albumin (by binding to its lysine residues), and the albumin concentration was determined spectrophotometrically from the intensity of light absorbance by the resulting yellow complex at 610 nm, calibrated against a series of BSA standards (0 to 50 mg/mL).

Total lipid concentration was measured in triplicate in pooled plasma samples (200 μL) extracted with 2-propanol:cyclohexane (ratio 4:5). The plasma/solvent mixture was vortexed for 1 min, subjected to ultrasonication for 2 min, and then centrifuged at 10,000 rpm for 10 min at 4 °C. Supernatant solvent extracts were then evaporated to dryness at 80 °C under vacuum in preweighed glass vials in a Genevac EZ-2 rotary evaporator (BioPharma Group, Winchester, UK) and dried further over silica gel in a desiccator. The lipid remaining in each glass vial was quantified gravimetrically.^57^ Lipid extraction efficiency was assessed based on the % recovery of 3 mg of glycerol trioleate (CAS number: 122-32-7) spiked into replicate plasma samples (200 μL).

### 2.5 New Empirical (*In Vitro*) Data on Plasma Protein Binding of APIs in Fish Compared with Humans

APIs tested for *in vitro* plasma protein binding (*n* = 44) (Supporting Information Table 4) included basic/cationic, acidic/anionic, zwitterionic, and neutral compounds with wide ranging physicochemical and pharmacokinetic properties (Figure 1). These APIs were obtained from commercial suppliers as “external” standards, along with corresponding deuterated “internal” standards, all with certified purity of >98–99%.

**Figure 1:**
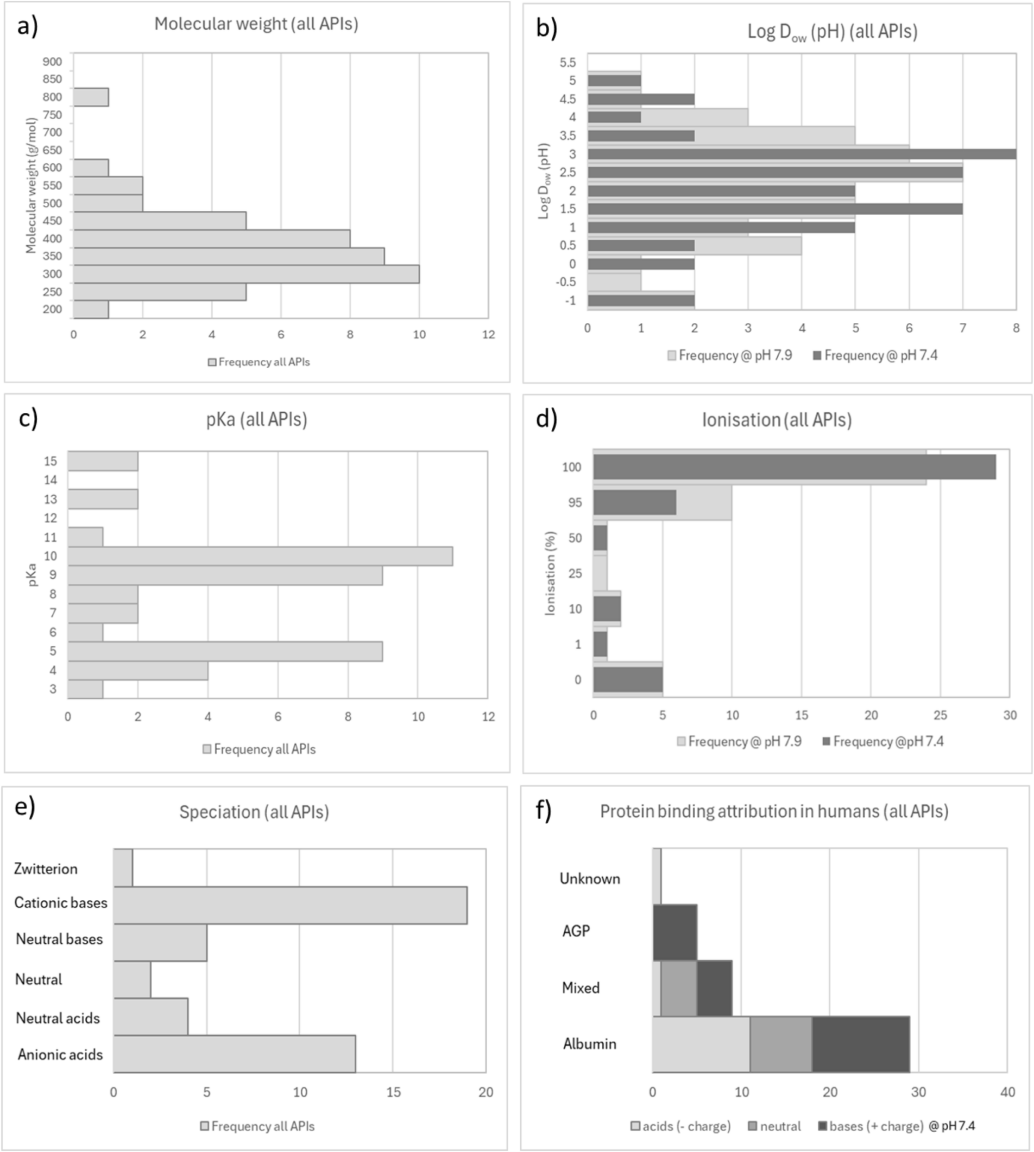
Physico-chemical and pharmacokinetic properties of APIs selected for protein binding assays. The frequency distributions of *n* = 44 APIs selected for *in vitro* protein binding assays are illustrated for the following: a) molecular weight; b) calculated pH-dependent distribution coefficient Log D_ow_ (pH); c) acid dissociation constant (p*K*_a_); d) ionization (%) at pH 7.4 and 7.9); e) speciation-discriminating acidic/basic and ionized/neutral compounds at pH 7.4–7.9 (note b) to e) were obtained from Chemaxon, Marvin 23.12); f) protein binding attribution in humans at pH 7.4 (See Supporting Information Table 3)

Plasma protein binding assays were conducted via rapid equilibrium dialysis (RED) according to the manufacturer’s instructions (Thermo-Fisher, UK)^58^ or HT Dialysis (HTD, Connecticut, USA)^59^ using buffers at species-specific plasma pHs and temperatures (Table 1). Binding assays were conducted in triplicate for each API, using blood plasma pooled from a minimum of 6 individuals for each test species. During dialysis, only unbound API was able to traverse from the plasma through the 8 kDa dialysis membrane into the buffer, while protein-bound API remained in the plasma. The materials incorporated in the dialysis membrane (cellulose) and base plate (high grade PTFE, Teflon) have low nonspecific binding potential,^58^ while dialysis cassettes (ABS-polystyrene) have moderate binding potential.^60^ Therefore, we measured total API recovery (from plasma and buffer) to account for any nonspecific binding.

**Table 1:** Species-Specific Blood Plasma pHs and Relevant Physiological Temperatures Applied for Plasma Protein Binding Assays

	Human	Fathead minnow	Koi carp	Rainbow trout
Plasma (and buffer) pH	7.4	7.7	7.7	7.8–7.9
Plasma (and buffer) incubation temperature (°C)	37	25	25	11^a^ or 15

a Lower temperature for plasma protein binding assays in rainbow trout at the University of Helsinki.^61^

Plasma in each plasma protein binding assay (either 100% plasma for humans, rainbow trout, and koi carp or 10% plasma in pH-matched, isotonic buffer for fathead minnow) was spiked with API “external” standard solution, resulting in a final concentration of API in plasma of 10 μM. Dialysis buffer (e.g., HEPES buffer or phosphate-buffered saline at the required species-specific pH) and spiked plasma were transferred to their respective chambers in the dialysis plate, which was sealed to the air with an adhesive seal. After 4 to 6 h of orbital shaking in the dialysis plate, plasma and adjacent buffer samples were removed to labeled microcentrifuge tubes, vortexed, and left on ice for 15 min. For the majority of APIs, an internal standard (equivalent deuterated API) was added to the plasma, and buffer samples were collected from the dialysis plate prior to vortexing. API standards were then extracted from plasma and buffer samples using acetonitrile (1:1 or 1:2, sample:ACN), and LC–UV/vis/fluorescence or LC–MS/MS analysis was then used, according to the methods summarized in Supporting Information Table 5, to quantify the concentration of API in blood plasma samples versus the concentration of API in buffer.

Unbound fraction of API (f_u_) in human, koi carp, and rainbow trout plasma (all 100%) was calculated:

4 $${\mathrm{100\% plasma f}}_{u}=\mathrm{API concentration in buffer}/\mathrm{API concentration in plasma}$$

Unbound fraction of API (f_u_10) in 10% fathead minnow plasma was also calculated (note in this case, f_u_10 substitutes for f_u_ in eq 4); the equivalent unbound fraction of API in 100% fathead minnow plasma (f_u_FM_) was then calculated:

5 $${100\%\;\mathrm{plasma}\;f}_{\mathrm{u\_FM}}{=f}_{u}{10/(10-9\times f}_{u}10)$$

Relative fraction unbound (Rf_u_) was then calculated: f_u__fish/f_u__human (eq 3).

Plasma protein binding data were generated for a total of 44 APIs by the analytical laboratories of the University of Exeter (*n* = 6 APIs in human, rainbow trout, and fathead minnow), the University of Helsinki (*n* = 30 APIs in rainbow trout), and Lilly Research Laboratories (*n* = 39 APIs in human and/or rainbow trout, fathead minnow, and koi carp). Eleven of the 44 APIs were subjected to intercomparison across more than one analytical laboratory, and a further seven APIs underwent repeat analysis within laboratories.

Molecular predictors of f_u_ established for APIs in humans (molecular weight (MW), lipophilicity, here represented by pH-dependent distribution coefficient (Log D_ow_), acid dissociation constant (p*K*_a_), charge (+, -, ±, 0, for cationic, anionic, zwitterionic, and neutral APIs, respectively) were all included in Generalized Additive Models (GAMs) to identify statistically significant predictors of unbound API fractions (f_u_) for the 44 APIs in each fish species (as well as in humans). The “gam­()” function in the mixed GAM computation vehicle (mgcv) package^62^ was used on the R statistics platform^63^ to develop generalized additive models (GAMs) for each species. In each case, median measured f_u_ values for all 44 APIs were tested for normality (Shapiro–Wilk test) and then transformed using progressively more powerful transformations (log_10_, square root, fourth root) until a normal distribution was achieved—fourth root f_u_ values were used for all species. All molecular predictor variables (above) were included for each of the 44 APIs in the GAM models as continuous (untransformed) variables, apart from molecular charge, which was treated as categorical (with 1, 2, 3, 4 representing +, −, ±, 0). Predictor variables were modeled using smooth terms (s) in combination with basis functions, i.e., knots (k), to allow nonparametric fits with relaxed assumptions on the actual relationship between the response variable f_u_ and predictor variables. Smooth terms were set automatically, whereas k was initially set manually to 4, since the number of data points (*n* = 44 APIs) was <100.^64^ The gam.check function in the mgcv package was used to check that the manually chosen values for k (e.g., k = 4) were not too close to the estimated degrees of freedom in the model. Each GAM model assumes the effects of the smooth functions for each predictor variable are additive such that the link function of the predicted response variable (fourth root f_u_) is the sum of the nonlinear, smoothed functions of individual predictors.

Example: Fourth root f_u_ ∼ s­(MW, k = 4) + s­(LogD_ow_, k = 4) + s­(p*K*_a_, k = 4) + s­(Charge, k = 4), family = Gamma (link = “log”)).

GAM model fit was evaluated using deviance explained (DE %) as a metric representing the proportion of the total deviance in the response variable that the model explains. DE % was used in preference to the adjusted R-squared value (R-sq­(adj)).

## 3 Results

### 3.1 Evaluation of Published Articles Characterizing Plasma Proteins in Fish

Published articles on genome sequencing and annotation relating to plasma proteins in fish indicate that most fish species lack albumin,\ due to evolutionary loss in (i) Acanthopterygii—superorder of bony fish containing 60% of all fish species, including gobies, perches, rice fish, killifish, and cichlids; (ii) Ostariophysi—superorder of bony fish containing 68% of freshwater fish, including carps, minnows, suckers, characins, and catfishes;^30^ and (iii) Chondrichthyes—cartilaginous fish.^34,65^ Albumin-like proteins are, however, conserved in the evolutionarily more ancient salmonids, such as rainbow trout (*Oncorhynchus mykiss*), spotted gar (*Lepisosteus oculatus*), northern pike (*Esox lucius*), and Australian lungfish (*Neoceratodus forsteri*).^30,34^ As in the case for albumin, AGP appears to be absent in most fish, with a few exceptions, including rainbow trout.^35^

Published data concerning plasma protein molecular weights and palmitate binding potentials, which are indicative of albumin-like proteins in evolutionarily more ancient fish species,^39^ largely conform with the available genomic data. Furthermore, apolipoproteins, which are numerous in virtually all fish, appear to have ligand-binding properties similar to those of albumin. For example, in cartilaginous fish, palmitate binds preferentially to LDL and VLDL apolipoproteins,^38^ and in some bony fish, including common carp (*Cyprinus carpio*), palmitate binds preferentially to HDL apolipoproteins.^66^ Our literature search found that data quantifying the importance of different plasma proteins for binding anionic, cationic, and neutral APIs are lacking for fish.

### 3.2 Evaluation of Current Genomic Data to Assess the Conservation of Plasma Proteins in Fish Compared with Humans

Across the current genome data analyzed using Orthofinder,^40^ 27 orthogroups were found, two of which contained genes encoding albuminoids (ALB and GC), one represented AGP (ORM1–3), and the remaining 24 encoded apolipoproteins (Figure 2). The albuminoid orthogroups ALB and GC were found in all tetrapods but not in all teleosts. ALB included human serum albumin and albumin-like genes from the Salmoniformes, Esociformes, Osteoglossiformes and the soldierfish *Myripristis murdjan*, and the other albuminoids afamin (AFM) and alpha-fetoprotein (AFP), which were not found in any fish species (Supporting Information Figure 1). While the tetrapods had single copies of ALB, AFM, and AFP, multiple gene duplication events in some fish species (e.g., pike and salmonids) have resulted in two or three genes encoding an albumin-like protein. GC included the gene encoding vitamin D binding protein (DBP), which was found in all tetrapods and cyprinids, as well as the electric eel and Asian arowana. The genes for AGP (ORM1–3) were found in most tetrapods but not in any fish species.

**Figure 2:**
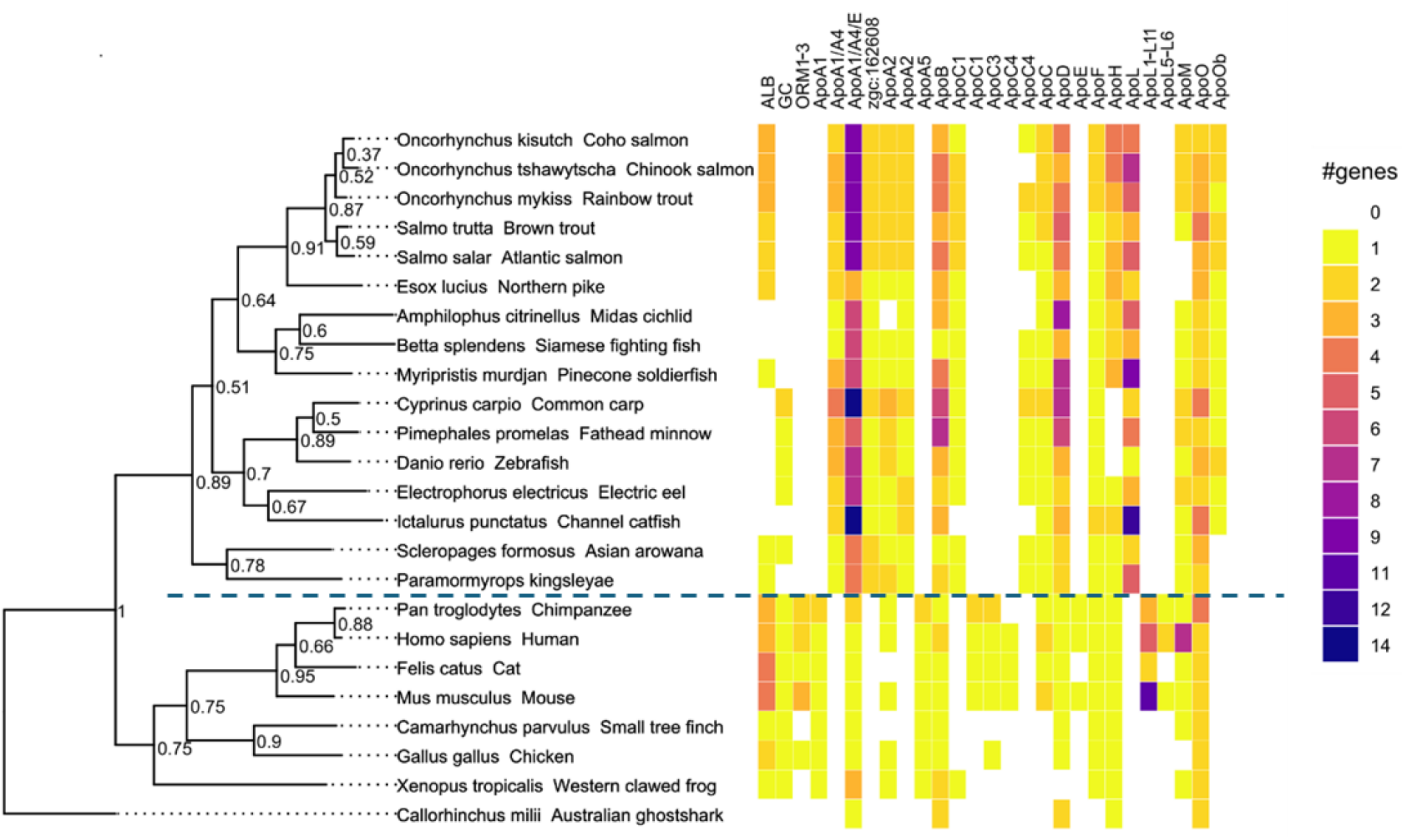
Phylogenetic conservation of blood plasma proteins between teleost fish and tetrapods according to amino acid sequence data. Genome data were analyzed using Orthofinder: species are listed in the first “text” column; teleost fish are located above the dashed line and tetrapods (and ghost shark, rooting the phylogenetic tree) below it. The following columns indicate the numbers of genes representing orthogroups for plasma proteins in each species. ALB = albuminoids, GC = group-specific component gene (albuminoid), ORM = orosomucoid, also known as alpha-1-acid glycoprotein (AGP), Apo = apolipoproteins.

A wide range of genes encoding apolipoproteins were found among the teleost fish (Figure 2). The orthogroups differentiated by OrthoFinder did not always reflect gene nomenclature, and for example, in many cases, homonymous genes were split between orthogroups, while ApoA1, ApoA4, and ApoE were grouped together. All major apolipoprotein groups identified in the tetrapods also occurred in the teleosts, apart from ApoA5, which was not found in any of the teleosts. Multiple gene duplication events occurred during the evolution of the teleosts and resulted in higher genetic diversity in comparison to the tetrapods (such as for ApoA1/A4/E, ApoD, and ApoB; Figure 2, Supporting Information Figures 2–10).

### 3.3 Evaluation of Published Data on Plasma Protein Binding of APIs in Fish Compared with Humans

The Phish-Pharm database^43^ contained plasma protein binding data for 14 APIs (representing antibiotics and antifungals). Here, plasma-bound API fractions were quantified via centrifugal ultrafiltration of blood plasma samples taken from fish, following *in vivo* exposure to each API—with 11 APIs measured in channel catfish, 9 APIs in rainbow trout, and 1–2 APIs measured in other teleost fish, including ayu (*Plecoglossus altivelis*), amberjack (*Seriola dumerili*), common carp (*Cyprinus carpio*), and European eel (*Anguilla anguilla*). Data quantifying plasma protein binding for a further 14 APIs were obtained for rainbow trout from other published literature.^18,19^ In these published studies, binding was quantified via dialysis following the *in vitro* spiking of blood plasma samples. The dialysis buffer for this published work was pH 7.4, which is appropriate for humans, rather than being physiologically relevant (pH 7.9) for rainbow trout. Even so, the relative fraction of unbound API (Rf_u_) was found to be highest for anionic APIs, including NSAIDs (Naproxen = 82 and Ibuprofen = 15; the diuretic:torasemide = 21 and the anticoagulant:warfarin = 17).^18^ All obtained fish plasma protein binding data (and Supporting metadata) compiled from this literature search, along with published data for humans,^44–46^ are presented in Supporting Information Table 6. Predictor variables for unbound fractions of API (f_u_) in fish could not be discerned from the published data.

### 3.4 Analysis of Blood and Blood Plasma Composition in the Selected Model Fish Species Compared with Humans

Total plasma protein concentrations measured in the three selected fish species were similar (35–40 mg/mL) (Table 2) and fell within the range reported in the literature for these and other fish species (20 and 80 mg/mL) (Supporting Information Table 7). Total plasma protein concentrations measured in humans (60 mg/mL) resembled previously reported concentrations (65 and 86 mg/mL)^67,68^ and were higher than those measured in our study fish (Table 2). Higher total blood plasma protein concentrations in humans were, however, compensated to some extent by higher proportions of blood cells versus plasma (i.e., hematocrits ranged from 37 to 52% in humans, compared to 28–34% in our tested fish species). The fish species analyzed were also found to have lower albumin concentrations (23–28 mg/mL) compared with humans (55 mg/mL). The detection of albumin in fathead minnow and koi carp contrasts with their genome sequencing data, which indicate an absence of this protein (Section 3.2). Concentrations of albumin detected within fathead minnow and koi carp also showed considerable variation (with means and standard deviations of 23.0 ± 20.2 (i.e., ± 75%) and 28.4 ± 21.3 (i.e., ± 88%), respectively). Total lipid concentrations in fish were higher than in humans (5 mg/mL); they were two times higher in rainbow trout (11 mg/mL) and four times higher in both fathead minnow and koi carp (19 and 21 mg/mL, respectively) (Table 2).

**Table 2:** Blood Plasma Composition in Selected Model Fish Species Compared with Humans^a^,^b^

		Human	Rainbow trout	Koi carp	Fathead minnow
**Blood pH**	Mean	7.4	7.89	7.68	7.72
	st. dev.	0.04	0.17	0.1	0.12
**Hematocrit**	Mean (%)	37–52 ^69^	30	30–34 ^70^	28
	st. dev.	-	1.0	-	1.2
**Total plasma protein**	Mean (mg/mL)	60.1	35.0	38.5	39.7
	st. dev.	9.5	3.2	1.9	0.6
**Plasma albumin (BCP assay)**	Mean (mg/mL)	55.4	28.2	23.0	28.4
	st. dev.	2.5	0.9	20.2	21.3
**Total plasma lipid**	Mean (mg/mL)	5.0	10.6	21.2	19.2
	st. dev.	0.5	0.6	1.4	0.5

a Published reference hematocrit values for human blood^69^ and koi carp blood.^70^

b All other parameters were measured in this study.

### 3.5 New Empirical (*In Vitro*) Data on Plasma Protein Binding of APIs in the Selected Fish Species Compared with Humans

Total recovery from plasma and buffer following dialysis was >70% for the majority of APIs, while lower recovery rates (>50%) were recorded for 6/44 APIs, potentially indicating nonspecific binding for these APIs (Supporting Information Table 8). Other published data concerning nonspecific binding for both anionic and cationic APIs indicate that these lower recoveries are unlikely to substantially affect the quantification of plasma protein binding.^71^

Variation in plasma protein binding and fractions of unbound API (min-max f_u_ range) measured in fish (within species) and in humans for the intra- and inter-laboratory comparisons were generally less than 3× (Supporting Information Table 9). To provide a robust assessment of the relative fraction of unbound API in each fish species, both median and maximum Rf_u_ values were calculated as follows:

6 $$\mathrm{Median ratio of fraction unbound}\left({\mathrm{Rf}}_{u}\right){=f}_{u}\mathrm{\_median\_fish}/f_{u}\mathrm{\_median\_human}$$

7 $$\mathrm{Maximum ratio of fraction unbound}\left({\mathrm{Rf}}_{u}\right){=f}_{u}\mathrm{\_max\_fish}/f_{u}\mathrm{\_min\_human}$$

Rf_u_ was generally highest in rainbow trout, with the greatest number of APIs having a maximum Rf_u_ of ≥ 10 (Figure 3a). These APIs included 8/13 (62%) anionic APIs, 2/11 (18%) unionized APIs, and 1/20 (5%) cationic APIs (Table 3). The highest maximum Rf_u_ values (in parentheses) were recorded in trout for anionic (A−) APIs, including Mycophenolic acid (56) and the NSAIDs Ibuprofen (55) and Phenylbutazone (100), while the lowest values (≤0.1) were recorded for the cationic (C+) phenothiazine antipsychotic drugs Levomepromazine, Azelastine, and Fluphenazine (Figure 3a). Maximum Rf_u_ values for APIs in koi carp and fathead minnow showed similar trends to those observed for rainbow trout, albeit with lower numbers of APIs with maximum Rf_u_ ≥ 10, i.e., 5/13 (38%) anionic APIs in each case and 1/11 (9%) unionized APIs in the fathead minnow (Figure 3b and c). Calculating median Rf_u_ for each fish species generated similar results to those for maximum Rf_u_, with almost identical proportions of anionic APIs exceeding the arbitrary threshold (3) for median Rf_u_ (Table 3). Comparing f_u_ between the cyprinids (koi carp and fathead minnow), we found that the majority of APIs tested were positioned on or close to the line of parity (Figure 3d). Furthermore, f_u_ was higher in trout compared with fathead minnow for the majority of APIs, regardless of ionic speciation (Figure 3e).

**Table 3:**
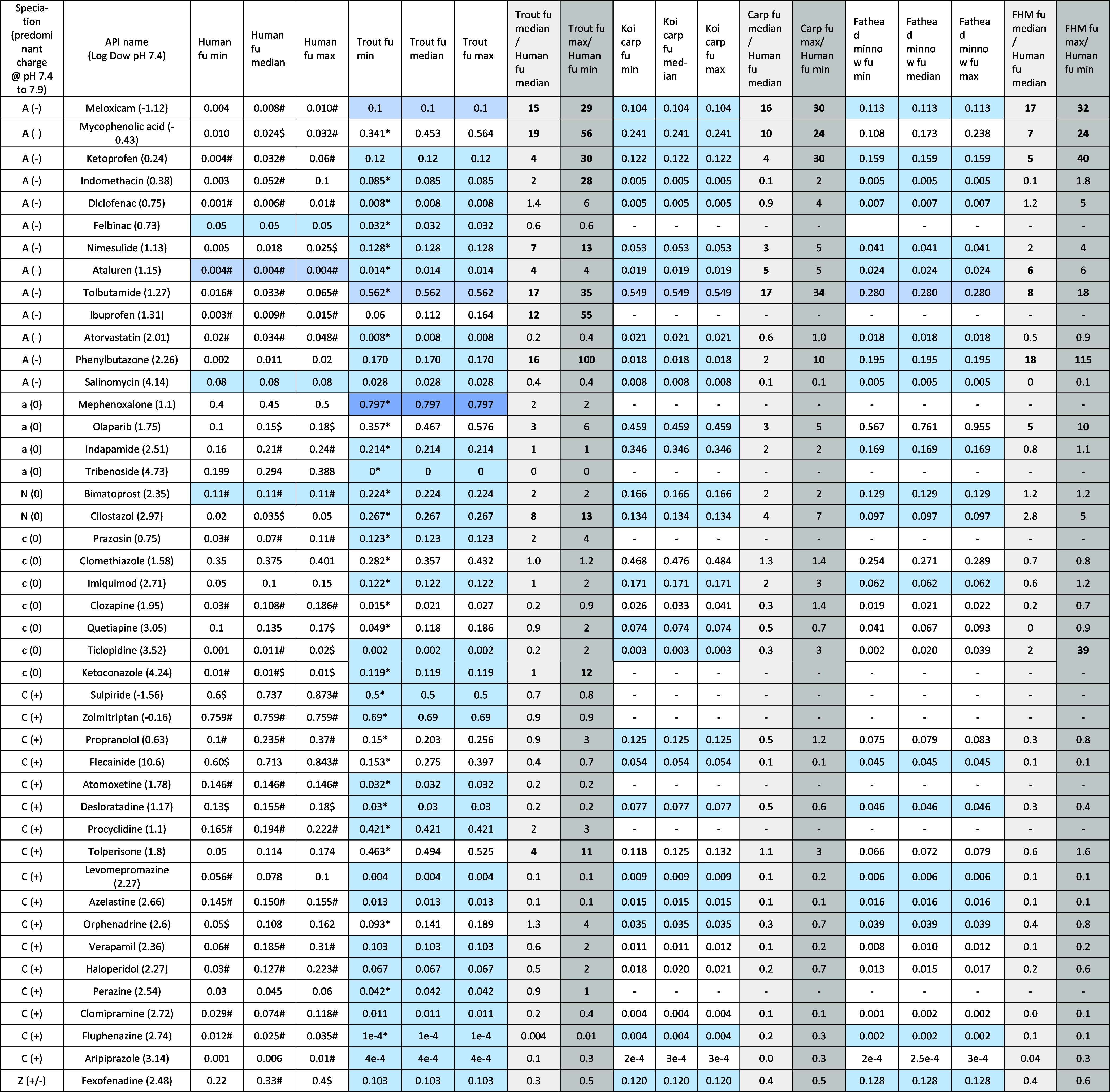
Relative Fractions of Unbound APIs (Rf_u_) in Selected Model Fish Species Compared with Humans^a^,^b^,^c^,^d^,^e^

a Speciation: A = Anion (+); a = Anionic (unionized); *N* = Neutral (0); C = Cation (−); c = Cationic (unionized); Z = Zwitterion (±).

b Adjacent blue-highlighted cells for each species contain identical f_u_ min, median, and max values; in each case, these f_u_ values represent the mean of *n* = 3 replicate measurements made for each species by an individual laboratory. Differing min, median, and max values are taken from more than one laboratory in this study.

c Gray-highlighted cells contain median and maximum relative fractions of unbound API (Rf_u_) (eqs 6 and 7) and **emboldened values** are ≥ 3 and ≥ 10, respectively.

d # and $ indicate data for f_u_ in humans from two available databases.^45,46^

e * indicates that the primary data for f_u_ in rainbow trout are published in parallel work to this study.^61^

**Figure 3:**
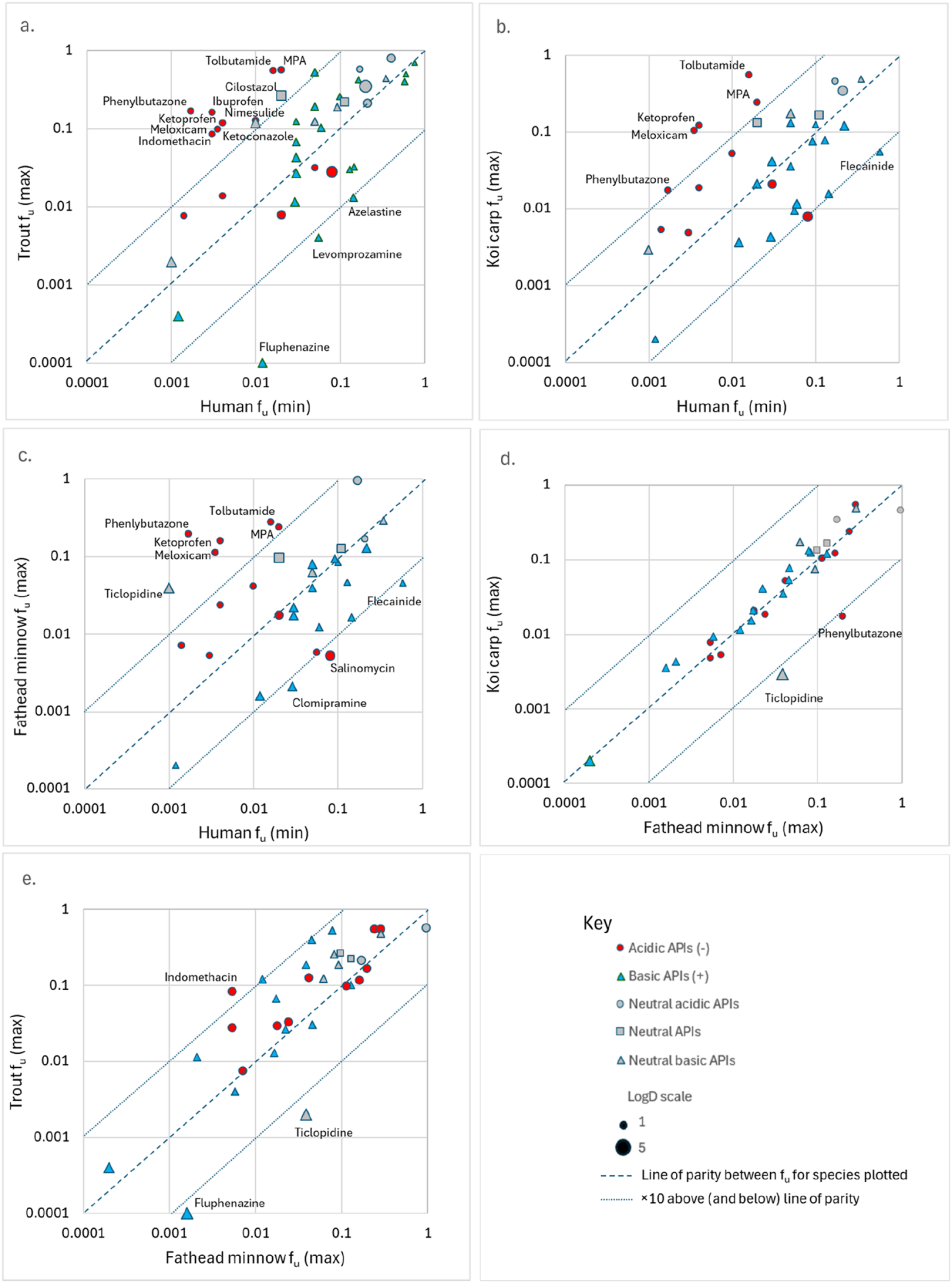
Comparison of fraction of unbound API (f_u_) for acidic/anionic and basic/cationic APIs in fish and human blood plasma (APIs more than ×10 above/below the line of parity are annotated).

To better account for API availability in fish compared to humans, Rf_u_ can be incorporated into the prediction of the therapeutic water concentration (TWC) for APIs in each fish species using the following equation:

8 $$\mathrm{TWC}=\left(C_{\max}{/\mathrm{Rf}}_{u}\right){/P}_{\mathrm{blood:water}}$$

For 6/13 anionic (A−) APIs in rainbow trout, incorporating maximum Rf_u_ reduced TWC by between 10 and 100× compared with TWC calculated using the original FPM model. Conversely, for 12/20 cationic (C+) APIs in rainbow trout, incorporating maximum Rf_u_ reduced TWC by <10×, and for a further 7/20 cationic APIs, this increased TWC (Table 4). Similar trends were observed for TWCs generated using the median (as well as maximum) Rf_u_ for rainbow trout and for koi carp and fathead minnow (Supporting Information Table 10).

**Table 4:** Therapeutic Water Concentrations Predicted Generically for “Fish” Using the Original FPM and Predicted Specifically for Rainbow Trout Using a Refined FPM with Rf_u_ Correction^a^,^b^,^c^,^d^,^e^,^f^

Speciation (predominant charge @ pH 7.4 to 7.9)	API name	Log D_ow_ (pH 7.4) used for “fish”	Pb:w for “fish”	Log D_ow_ (pH 7.9) used for trout	Pb:w for trout	Human *C*_max_ (mg/L)	Trout f_u_ max/Human f_u_ min	TWC original FPM for “fish”(mg/L)	TWC refined FPM for trout (mg/L)	TWC original/TWC refined	NOEC for fish (mg/L)	LOEC for fish (mg/L)	TWC original/LOEC	TWC refined/LOEC
A (−)	Meloxicam	–1.12	0.02	–1.75	0.01	0.4	29	19.94	2.01	9.91				
A (−)	Mycophenolic acid	–0.43	0.06	–0.58	0.05	1 ^77^	35	15.63	0.57	27.20	0.1 ^81^	0.3 ^81^	52.1	1.9
A (−)	Ketoprofen	0.24	0.20	–0.02	0.13	4.22	30	21.39	1.10	19.38				
A (−)	Indomethacin	0.38	0.25	0.02	0.14	0.025	28	0.10	0.01	15.29				
A (−)	Felbinac	0.73	0.45	0.31	0.22	0.86 ^73^	0.6	1.91	6.46	0.30				
A (−)	Diclofenac	0.75	0.47	0.49	0.30	0.5	6	1.08	0.28	3.88	0.32 ^82^	1.0 ^82^	1.1	0.3
A (−)	Mephenoxalone	1.1	0.84	1.1	0.84	2.85 ^76^	2	3.40	1.70	2.00				
A (−)	Nimesulide	1.13	0.88	0.94	0.64	0.1	13	0.11	0.01	9.45				
A (−)	Ataluren	1.15	0.91	0.92	0.62	2 ^72^	4	2.20	0.81	2.72				
A (−)	Tolbutamide	1.27	1.11	1.21	1.01	45	35	40.37	1.28	31.64				
A (−)	Ibuprofen	1.31	1.19	0.87	0.57	15	55	12.58	0.48	26.25	0.68 ^83^		18.5	0.7
A (−)	Atorvastatin	2.01	3.87	1.66	2.15	0.0164	0.4	0.004	0.02	0.22				
A (−)	Phenylbutazone	2.26	5.89	2.14	4.81	12.5	100	2.12	0.03	81.73				
A (−)	Salinomycin	4.14	138.74	3.77	74.49	-	0.4	-	-	-				
a (0)	Olaparib	1.75	2.50	1.75	2.50	7.3 ^78^	3	2.92	0.97	3.00	0.32 ^84^	1.0 ^84^	2.9	1.0
a (0)	Indapamide	2.51	8.96	2.5	8.81	0.0558	1	0.006	0.01	0.98				
a (0)	Tribenoside	4.73	374.02	4.73	374.02	-	0	-	-	-				
N (0)	Bimatoprost	2.35	6.85	2.35	6.85	0.00008	2	1.17 × 10^–05^	5.84 × 10^–06^	2.00				
N (0)	Cilostazol	2.97	19.41	2.97	19.41	1.2	13	0.062	0.0048	13.00				
c (0)	Clomethiazole	1.58	1.88	1.58	1.88	0.1	1	0.053	0.044	1.20				
c (0)	Imiquimod	2.71	12.54	2.71	12.54	0.00032	2	2.552 × 10^–05^	1.28 × 10^–05^	2.00				
c (0)	Quetiapine	3.05	22.21	3.16	26.72	0.1	2	0.005	0.0019	2.41	0.1 ^85^	0.32 ^85^	0.01	0.01
c (0)	Ketoconazole	4.24	164.13	4.26	169.75	4.22	12	0.026	0.002	12.41	0.006 ^86^	0.025 ^86^	1.03	0.08
C (+)	Sulpiride	–1.56	0.01	–1.09	0.02	0.05	0.8	5.22	2.96	1.76				
C (+)	Zolmitriptan	–0.16	0.10	0.32	0.23	0.007	0.9	0.069	0.03	2.02	1 ^87^	3.2 ^87^	0.02	0.01
C (+)	Flecainide	0.6	0.36	1.08	0.81	0.4	0.7	1.11	0.71	1.57				
C (+)	Propranolol	0.63	0.38	1.11	0.85	0.02	3	0.053	0.01	6.72				
C (+)	Prazosin	0.75	0.47	1.09	0.82	0.001	4	0.002	0.0003	7.08				
C (+)	Procyclidine	1.1	0.84	1.59	1.91	0.08	3	0.096	0.01	6.84				
C (+)	Desloratadine	1.17	0.94	1.63	2.04	0.004	0.2	0.004	0.01	0.43				
C (+)	Atomoxetine	1.78	2.63	2.26	5.89	0.16	0.2	0.061	0.14	0.45				
C (+)	Tolperisone	1.8	2.72	2.26	5.89	0.064 ^80^	11	0.024	0.001	23.83				
C (+)	Clozapine	1.95	3.50	2.41	7.57	0.3	0.9	0.086	0.044	1.95	0.018 ^83^	0.031 ^83^	2.8	1.4
C (+)	Haloperidol	2.27	5.99	2.64	11.15	0.005	2	0.0008	0.000	3.73				
C (+)	Levomepromazine	2.27	5.99	2.75	13.41	0.046 ^75^	0.1	0.008	0.034	0.22				
C (+)	Verapamil	2.36	6.96	2.84	15.60	0.02	2	0.003	0.001	4.48	0.3 ^83^	0.6 ^83^	0.005	0.001
C (+)	Perazine	2.54	9.42	2.99	20.08	0.008 ^79^	1	0.0008	0.000	2.13				
C (+)	Orphenadrine	2.6	10.42	3.07	22.97	0.1	4	0.010	0.001	8.81				
C (+)	Azelastine	2.66	11.53	3.11	24.56	0.002	0.1	0.0002	0.001	0.21				
C (+)	Clomipramine	2.72	12.75	3.21	29.06	0.09	0.4	0.007	0.008	0.91	0.01 ^88^	0.1 ^88^	0.07	0.08
C (+)	Fluphenazine	2.74	13.19	3.19	28.10	0.027 ^73^	0	0.002	-	-				
C (+)	Aripiprazole	3.14	25.83	3.62	57.89	0.0273	0.3	0.001	0.002	0.67				
C (+)	Ticlopidine	3.52	48.93	3.8	78.34	0.31	2	0.006	0.002	3.20				
Z (±)	Fexofenadine	2.48	8.52	2.47	8.38	0.131	0.5	0.015	0.031	0.49				

a API speciation: A = Anion (+); a = Anionic (unionized); *N* = Neutral (0); C = Cation (−); c = Cationic (unionized); Z= Zwitterion (±).

b Therapeutic water concentration (TWC) for “fish” = *C*_max_/P_blood:water_ Original FPM (eq 2).

c Therapeutic water concentration (TWC) for rainbow trout = (*C*_max_/Rf_u_)/P_blood:water_ Refined FPM (eq 8).

d Rf_u_ = Trout f_u_ max/Human f_u_ min, and P_Blood:Water_ = 10^(0.73 × Log Dow – 0.88)^.

e Log D_ow_ (pH 7.4) was used for “fish”, and Log D_ow_ (pH 7.9) was used for rainbow trout; values were obtained from Chemaxon, Marvin 23.12. Human *C*_max_ values (mg/L) were obtained mainly from one reference.^44^ Additional data (annotated in the table) were obtained from additional references.^72–80^

f For conservative prediction: TWC ≤ Lowest Observed Effect Concentration. Note: LOECs (and NOECs) were obtained from standardized fish early life-stage tests (OECD 210 guideline) refs. 81–88 .

Refined TWCs incorporating Rf_u_ were more conservative than TWCs calculated using the original FPM model in relation to LOECs for APIs with available chronic effects data for fish (OECD 210 guideline). More specifically, refined TWCs were lower than the lowest observed effect concentration (LOEC) for 2/3 anionic (A−) APIs and 3/3 neutral anionic (a0) APIs. Meanwhile, refined TWCs for cationic (C+) and neutral cationic (c0) APIs were virtually indistinguishable from original TWCs, and collectively (in 5/6 cases), they were (both) lower than reported LOECs (Table 4 and Supporting Information Table 10).

GAM models indicated that statistically significant predictor variables for f_u_ in all three fish species included API lipophilicity (Log D_ow_ (pH)) and API ionization (Charge) (Table 5). The GAM model for koi carp best fit the data for the 44 APIs (R-sq (adj) = 0.48; DE = 64.1%), followed by fathead minnow (R-sq­(adj) = 0.433; DE = 55.8%). GAM models for rainbow trout and humans exhibited poorer fit with the data for the 44 APIs; R-sq­(adj) and DE were lower than those for fathead minnow and koi carp (Table 5).

**Table 5:** Molecular Predictors of Fu in Fish Compared with Humans according to a Generalized Additive Model (GAM)^a^,^b^,^c^

Species	Significant Predvar(s)	Model R-sq. (adj)	Deviance explained (%)
Human	MW*	0.396	33.4
	p*K*_a_**		
	Charge*		
Rainbow trout	Log D_ow_ (pH 7.9) **	0.34	34.1
	Charge *		
Koi carp	Log D_ow_ (pH 7.7) ***	0.48	64.1
	Charge ***		
Fathead minnow	Log D_ow_ (pH 7.7) **	0.433	55.8
	Charge***		

a **Model:** fourth root f_u_ ∼ s­(MW, k = 4) + s­(LogD_ow_, k = 4) + s­(p*K*_a_, k = 4) + s­(Charge, k = 4), family = Gamma (link = “log”).

b Predictor variables (Predvars) are MW, Log D_ow_, p*K*_a_, and Charge; s is a smooth term; k is a basis function (knot).

c Predvar significance *p*-value: *** 0.001; ** 0.01; * 0.05.

## 4 Discussion and Implications

The fish plasma model (FPM) is a valuable tool for screening and prioritizing active pharmaceutical ingredients (APIs) for *in vivo* fish testing, a key testing component in environmental risk assessment guidelines for pharmaceuticals implemented since 2006.^89,90^ An API’s threshold environmental effect concentration (i.e., therapeutic water concentration, TWC) can be predicted for fish using the FPM—based on the total therapeutically effective plasma concentration in humans (*C*_max_)—assuming that human drug targets are conserved in fish. However, only the unbound fraction of API (f_u_) is available for pharmacological action on drug targets in the body, and this fraction can show considerable interspecies variation, as demonstrated for a variety of mammalian test species used to assess drug availability and safety for humans.^91,92^ Given the likelihood of interspecies variation to occur also between mammals and fish, and with the aim of refining FPM predictions of therapeutic water concentrations for pharmaceuticals in fish, we undertook *in vitro* measurements of f_u_ for a diverse range of *n* = 44 APIs in blood plasma obtained from three model fish species: rainbow trout, koi carp, and fathead minnows, and for humans. We also assessed whether f_u_ could be predicted reliably for these model fish species based on key molecular variables (MW, lipophilicity, and charge) established for APIs in humans.

Our *in vitro* results show that the relative fraction of unbound API (Rf_u_ = f_u_ in fish/f_u_ in humans) is greater than a factor of 10 for anionic APIs (supporting our first hypothesis that f_u_ is higher in fish than in humans for anionic APIs). The evidence was most compelling for rainbow trout, in which Rf_u_ was more than 1 order of magnitude higher for 8/13 (62%) of the anionic APIs tested, compared with 5/13 (38%) anionic APIs in both koi carp and fathead minnow. The lower Rf_u_ values for koi carp and fathead minnow, lacking albumin orthologues, were unexpected, given the known importance of albumin for binding anionic APIs in humans^21,25^ (seemingly failing to support our second hypothesis: f_u_ is higher in fish that lack albumin).

In our study, albumin was quantified in blood plasma using the bromocresol purple (BCP) assay, and its detection in koi carp and fathead minnow, in the absence of any known albumin orthologues and with much greater variability in detected concentrations than in rainbow trout and humans, suggests that BCP can bind and form a yellow complex with more proteins than albumin alone. The unexpected detection of albumin in both cyprinid fish species suggests that the BCP method may not entirely distinguish albumin from other albumin-like proteins in these species. We recommend, therefore, that the fidelity of the BCP assay should be clarified in more fish species that possess albumin orthologues versus other species in which orthologues are absent. Other published studies quantifying protein molecular weight and electrophoretic mobility have indicated the presence of “albumin-like” proteins in carp,^93,94^ as well as in Atlantic killifish (*Fundulus heteroclitus*) and Pacific surfperch (*Neoditrema ransonnetii*),^22^ all of which lack albumin orthologs^30^ (Supporting Information Table 11). Quantifying plasma protein binding of palmitate and reference pharmaceuticals (e.g., the anionic API ibuprofen that binds primarily to albumin) would help consolidate understanding in assessments of the presence of “albumin-like” proteins in fish. Similarly, employing reference cationic APIs, for example, propranolol that binds to AGP in humans, together with quantification of protein molecular weight and electrophoretic mobility, would provide further confidence in detecting the presence of “AGP-like” proteins in fish.

Our results, together with existing evidence^18,19^ showing higher Rf_u_ for anionic APIs in fish compared with humans, indicate that fish testing may be required for more APIs than previously indicated by prioritizations based on the original FPM model.^7,8,10^ It is, however, important to note that, based on extensive analysis of dissociation (p*K*_a_) data for small molecule pharmaceuticals,^95,96^ the proportion of anionic APIs (among the estimated >2000 legacy APIs without an environmental risk assessment) is likely to be less than 25%, whereas cationic compounds likely comprise more than 45% of APIs. Before implementing a refined FPM for (re)­prioritizing thousands of legacy APIs, we therefore recommend that *in vivo* testing is targeted on a diverse subset of anionic, neutral, and cationic APIs to assess their uptake and effects in different fish species. In this way, the accuracy and level of conservatism of TWC predictions using both the original version and the refined version of the FPM can be fully assessed (eq 2 and eq 8). This will furthermore help to better understand uncertainties in FPM predictions, which are currently estimated to sum to 3 orders of magnitude, comprising (i) ×10 for extrapolation from humans to mammals; (ii) ×10 for differences among mammalian species; and (iii) ×10 for extrapolation from mammalian to nonmammalian species.^6^

Our intra- and interlaboratory comparisons show that the range of unbound fractions of APIs in each test species was generally less than ×3, therefore requiring a threshold of 3 for defining substantive differences between fish and humans based on median Rf_u_ (eq 6). A higher threshold of 10 was used to highlight substantive interspecies differences based on maximum Rf_u_ (eq 7)—effectively implementing a generic assessment factor of ×10. The latter is a worst-case approach, which is contingent on establishing a realistic range of f_u_ for APIs in both humans and model fish species. Variation in f_u_ between human subjects may range up to ×10 for some anionic APIs, such as diclofenac and indomethacin, and up to ×6 for some cationic APIs.^45^ This variation in f_u_ in humans is often attributed to genetic diversity. For example, f_u_ for propranolol has been shown to be substantially higher (×10 times higher) in Chinese subjects compared to Caucasian subjects. Elevated f_u_ in this instance has been attributed to lower plasma concentrations of the acute-phase protein AGP, which plays a key role in binding this and other cationic drugs.^97^ AGP is also linked to immune response and, as such, blood plasma AGP concentrations, and thus, binding of cationic APIs may vary substantially between healthy and diseased patients.^98^ Disease and aging in humans can also affect concentrations of albumin and its conformation, in turn affecting API binding to this plasma protein.^99,100^ Exogenous substances can furthermore modulate albumin binding of APIs (e.g., tolbutamide), including flavonoids, which are prevalent in vegetarian diets.^101^ Variation in f_u_ within and between fish species has also been linked to variation in genetics, age, and exposure to environmental factors, including environmental pH, temperature, and food availability.^39,64,102^ Nevertheless, empirical data quantifying f_u_ in fish blood plasma remain relatively scarce, and *in silico* predictions of f_u_ in fish are not always accurate, even according to simpler mechanistic models that simulate lipophilic partitioning of neutral compounds.^103^

Molecular predictors of f_u_ (MW, lipophilicity, p*K*_a_ and charge) established for ionizable APIs in humans and mammals^37^ were assessed in humans and fish by applying Generalized Additive Models (GAMs) to f_u_ and molecular data for our diverse set of 44 APIs. Three of the four predictors (MW, p*K*_a_ charge) were highlighted as significant for humans, while only two (lipophilicity and charge) were significant predictors of f_u_ in rainbow trout, fathead minnow, and koi carp. The GAM model fit was best for koi carp (R-sq­(adj) = 0.48; DE = 64.1%), indicating limited accuracy of the model for predicting f_u_ in this species, as well as other fish species tested here. These results may be due, in part, to the limited data set of 44 APIs, as well as interspecies differences in plasma protein composition and physiological conditions, including blood pH and temperature, which are known to affect plasma protein binding.^104^ It is important to note that intra- and inter-species variations in the free fraction of an API in blood plasma may also arise from factors other than plasma protein binding, including API transport and metabolism, and these may differ considerably between humans and fish.^105^ Furthermore, human therapeutic plasma concentrations (*C*_max_) can also vary hugely between individual patients, for example by more than ×10 in the case of anionic APIs, such as mycophenolic acid, and cationic APIs, such as perazine and tolperisone.^46^ Variation in *C*_max_ (as with variation in f_u_) has major implications for the prediction of therapeutic water concentrations of APIs, which are considered likely to elicit effects in fish. Minimum reported *C*_max_ values were used in the work presented here for the original and refined versions of the FPM for predicting therapeutic water concentrations (eq 2 and eq 8).

We conclude that the FPM needs refinement through the application of relative fraction unbound (Rf_u_) to human therapeutic plasma concentrations (*C*_max_) to more accurately predict an API’s threshold environmental effect concentration for fish (i.e., therapeutic water concentration, TWC). This is especially important when using the FPM to screen and prioritize anionic APIs for fish testing because Rf_u_ for these APIs is frequently greater than a factor 10, and the TWC is correspondingly lower than predicted by the currently used version of the FPM.^8,10^ Furthermore, our *in vitro* data for anionic APIs indicate substantially higher Rf_u_ in rainbow trout (a salmonid fish species) compared to other (cyprinid) fish species that we tested, and, on this basis, we advocate the continued use of the rainbow trout as a model species in ongoing refinements of the FPM. We also highlight that predicting f_u_ *in silico* is less straightforward than measuring f_u_ *in vitro,* and this was particularly evident in the case of our analyses with rainbow trout. We emphasize that a deeper understanding of fish plasma proteins, including apolipoproteins and plasma lipids with affinities for lipophilic ligand binding, is required to improve *in silico* predictions of f_u_ in fish. Nevertheless, *in vitro* assays offer a practicable means for measuring this important parameter, and using large-sized rainbow trout offers the advantage of providing larger volumes of blood plasma, enabling the *in vitro* testing of more APIs per fish, thus supporting the reduction, refinement, and replacement (3Rs) of animal testing.

## Supplementary Material



## Notes

Plasma proteins in fish and humans and analytical methods for the quantification of APIs in their blood plasma (PDF)

## References

[1] Gunnarsson L., Jauhiainen A., Kristiansson E., Nerman O., Larsson D. G. (2008). Evolutionary
conservation of human drug targets in organisms used for environmental
risk assessments. Environ. Sci. Technol..

[2] Brown A. R., Gunnarsson L., Kristiansson E., Tyler C. R. (2014). Assessing variation
in the potential susceptibility of fish to pharmaceuticals, considering
evolutionary differences in their physiology and ecology. Philos. Trans. R. Soc., B.

[3] Verbruggen B., Gunnarsson L., Kristiansson E., Österlund T., Owen S. F., Snape J. R., Tyler C. R. (2018). ECOdrug: A database
connecting drugs and conservation of their targets across species. Nucleic Acids Res..

[4] Wilkinson J. L., Boxall A. B. A., Kolpin D. W., Leung K. M. Y., Lai R. W. S., Galbán-Malagón C., Adell A. D., Mondon J., Metian M., Marchant R. A., Bouzas-Monroy A., Cuni-Sanchez A., Coors A., Carriquiriborde P., Rojo M., Gordon C., Cara M., Moermond M., Luarte T., Petrosyan V., Perikhanyan Y., Mahon C. S., McGurk C. J., Hofmann T., Kormoker T., Iniguez V., Guzman-Otazo J., Tavares J. L., Gildasio
De Figueiredo F., Razzolini M. T. P., Dougnon V., Gbaguidi G., Traoré O., Blais J. M., Kimpe L. E., Wong M., Wong D., Ntchantcho R., Pizarro J., Ying G. G., Chen C. E., Páez M., Martínez-Lara J., Otamonga J. P., Poté J., Ifo S. A., Wilson P., Echeverría-Sáenz S., Udikovic-Kolic N., Milakovic M., Fatta-Kassinos D., Ioannou-Ttofa L., Belušová V., Vymazal J., Cárdenas-Bustamante M., Kassa B. A., Garric J., Chaumot A., Gibba P., Kunchulia I., Seidensticker S., Lyberatos G., Halldórsson H. P., Melling M., Shashidhar T., Lamba M., Nastiti A., Supriatin A., Pourang N., Abedini A., Abdullah O., Gharbia S. S., Pilla F., Chefetz B., Topaz T., Yao K. M., Aubakirova B., Beisenova R., Olaka L., Mulu J. K., Chatanga P., Ntuli V., Blama N. T., Sherif S., Aris A. Z., Looi L. J., Niang M., Traore S. T., Oldenkamp R., Ogunbanwo O., Ashfaq M., Iqbal M., Abdeen Z., O’Dea A., Morales-Saldaña J. M., Custodio M., de la Cruz H., Navarrete I., Carvalho F., Gogra A. B., Koroma B. M., Cerkvenik-Flajs V., Gombač M., Thwala M., Choi K., Kang H., Ladu J. L. C., Rico A., Amerasinghe P., Sobek A., Horlitz G., Zenker A. K., King A. C., Jiang J. J., Kariuki R., Tumbo M., Tezel U., Onay T. T., Lejju J. B., Vystavna Y., Vergeles Y., Heinzen H., Pérez-Parada A., Sims D. B., Figy M., Good D., Teta C. (2022). Pharmaceutical pollution of the world’s
rivers. Proc. Natl. Acad. Sci. U. S. A..

[5] Fitzsimmons P. N., Fernandez J. D., Hoffman A. D., Butterworth B. C., Nichols J. W. (2001). Branchial elimination of superhydrophobic organic compounds
by rainbow trout (*Oncorhynchus mykiss*). Aquat. Toxicol..

[6] Huggett D. B., Cook J. C., Ericson J. F., Williams R. T. (2003). A Theoretical
Model
for Utilizing Mammalian Pharmacology and Safety Data to Prioritize
Potential Impacts of Human Pharmaceuticals to Fish. HERA Hum. Ecol. Risk Assess.: Int. J..

[7] Fick J., Lindberg R. H., Tysklind M., Larsson D. G. (2010). Predicted critical
environmental concentrations for 500 pharmaceuticals. Regul. Toxicol. Pharmacol..

[8] Schreiber R., Gündel U., Franz S., Küster A., Rechenberg B., Altenburger R. (2011). Using the fish plasma model for comparative
hazard identification for pharmaceuticals in the environment by extrapolation
from human therapeutic data. Regul. Toxicol.
Pharmacol..

[9] Rand-Weaver M., Margiotta-Casaluci L., Patel A., Panter G. H., Owen S. F., Sumpter J. P. (2013). The read-across hypothesis and environmental
risk assessment
of pharmaceuticals. Environ. Sci. Technol..

[10] Coors A., Brown A. R., Maynard S. K., Nimrod Perkins A., Owen S., Tyler C. R. (2023). minimizing experimental
testing on
fish for legacy pharmaceuticals. Environ. Sci.
Technol..

[11] Nallani G., Venables B., Constantine L., Huggett D. (2016). Comparison of measured
and predicted bioconcentration estimates of pharmaceuticals in fish
plasma and prediction of chronic risk. Bull.
Environ. Contam. Toxicol..

[12] Huggett, D. B. ; Ericson, J. F. ; Cook, J. C. ; Williams, R. T. Plasma concentrations of human pharmaceuticals as predictors of pharmacological responses in fish. In Pharmaceuticals in the Environment–Sources, Fate, Effects and Risks., Kümmerer, K. , Eds.; Springer: Berlin, 2004; pp. 373–386.

[13] Brown J. N., Paxéus N., Förlin L., Larsson D. G. J. (2007). Variations in
bioconcentration of human pharmaceuticals from sewage effluents into
fish blood plasma. Environ. Toxicol. Pharmacol..

[14] Marmon P., Owen S. F., Margiotta-Casaluci L. (2021). Pharmacology-informed
prediction
of the risk posed to fish by mixtures of non-steroidal anti-inflammatory
drugs (NSAIDs) in the environment. Environ.
Int..

[15] Yan Z., Ma L., Carione P., Huang J., Hwang N., Kenny J.R., Hop C.E. (2024). Introducing
the dynamic well-stirred model for predicting hepatic
clearance and extraction ratio. J. Pharm. Sci..

[16] Summerfield S. G., Yates J. W. T., Fairman D. A. (2022). Free drug
theory – no longer
just a hypothesis?. Pharm. Res..

[17] Carlin, M. C. Pharmacology and mechanism of action of drugs, in Max M. In Houck, Encyclopedia of Forensic Sciences, Third ed.; Elsevier, 2023, Vol. 4, pp. 144–154. 10.1016/B978-0-12-823677-2.00086-6.

[18] Henneberger L., Klüver N., Mühlenbrink M., Escher B. (2022). Trout and human plasma
protein binding of selected pharmaceuticals informs the fish plasma
model. Environ. Toxicol. Chem..

[19] Nolte, T. M. ; Ryan, T. A. ; Gunnarsson, L. ; Verbruggen, B. ; Klüver, N. ; Owen, S. F. (2018). Binding of charged organic chemicals to fish plasma protein: Current data availability, modelling aspects and uncertainties; Accessed May 2025. https://www.researchgate.net/publication/353622425_Binding_of_charged_organic_chemicals_to_fish_plasma_protein_current_data_availability_modelling_aspects_and_uncertainties/citations.

[20] Eschmeyer, W. N. Genera, species, references; 2025, (Accessed February 2025): http://researcharchive.calacademy.org/research/ichthyology/catalog/fishcatmain.asp.

[21] Bteich M. (2019). An overview
of albumin and alpha-1-acid glycoprotein main characteristics: Highlighting
the roles of amino acids in binding kinetics and molecular interactions. Heliyon.

[22] Ceciliani F., Lecchi C. (2019). The immune functions of α1
acid glycoprotein. Curr. Protein Pept. Sci..

[23] Sudlow G., Birkett D. J., Wade D. N. (1975). Characterization of two specific
drug binding sites on human serum albumin. Mol.
Pharmacol..

[24] Ravis W. R., Parsons D. L., Wang S. J. (1988). Buffer and pH effects on propranolol
binding by human albumin and alpha 1-acid glycoprotein. J. Pharm. Pharmacol..

[25] Zsila F. (2013). Subdomain
IB is the third major drug binding region of human serum albumin:
Toward the three-sites model. Mol. Pharmaceutics.

[26] Smith S. A., Waters N. J. (2019). Pharmacokinetic
and Pharmacodynamic Considerations
for Drugs Binding to Alpha-1-Acid Glycoprotein. Pharm. Res..

[27] Schley J., Mueller-Oerlinghausen B. (1986). Investigation of the binding of various
tricyclic neuroletpics and antidepressants to alpha 1-acid glycoprotein. J. Pharm. Pharmacol..

[28] Kerkay J., Westphal U. (1968). Steroid-protein XIX.
Complex formation between a1-acid
steroid hormones. Biochim. Biophys. Acta.

[29] Matsumoto K., Sukimoto K., Nishi K., Maruyama T., Suenaga A., Otagiri M. (2002). Characterization of
ligand binding sites on the alpha1-acid
glycoprotein in humans, bovines and dogs. Drug
Metab. Pharmacokinet.

[30] Andreeva A. M. (2022). Evolutionary
transformations of albumin using the example of model species of jawless
Agnatha and bony jawed fish (Review). Inland
Water Biol..

[31] González-Durruthy M., Concu R., Vendrame L. F. O., Zanella I., Ruso J. M., Cordeiro M. N. D. S. (2020). Targeting beta-blocker drug-drug interactions with
fibrinogen blood plasma protein: A computational and experimental
study. Molecules.

[32] Shakour N., Ruscica M., Hadizadeh F., Cirtori C., Banach M., Jamialahmadi T., Sahebkar A. (2020). Statins and C-reactive protein: In
silico evidence on direct interaction. Arch.
Med. Sci..

[33] Li H., Qian Z. M. (2002). Transferrin/transferrin
receptor-mediated drug delivery. Med. Res. Rev..

[34] Andreeva A.
M. (2010). Structure
of fish serum albumins. J. Evol. Biochem. Physiol..

[35] Cairns M. T., Johnson M. C., Talbot A. T., Pemmasani J. K., Re M., Houeix B., Sangrador-Vegas A., Pottinger T. G. (2008). A cDNA
microarray assessment of gene expression in the liver of rainbow trout
(*Oncorhynchus mykiss*) in response to a handling and
confinement stressor. Comp. Biochem. Physiol.,
Part D: genomics Proteomics.

[36] Ascenzi P., Fasano M. (2010). Allostery in a monomeric protein:
The case of human
serum albumin. Biophys. Chem..

[37] Ma S., McGann M., Enyedy I. J. (2021). The influence
of calculated physicochemical
properties of compounds on their ADMET profiles. Bioorg. Med. Chem. Lett..

[38] Metcalf V. J., Gemmell N. J. (2005). Fatty acid transport in cartilaginous fish: Absence
of albumin and possible utilization of lipoproteins. Fish Physiol. Biochem..

[39] Enerstvedt K. S., Sydnes M. O., Pampanin D. M. (2017). Editorial Note to
“Identification
of an albumin-like protein in plasma of Atlantic cod (Gadus morhua)
and its biomarker potential for PAH contamination” [Heliyon
3 (8) (August 2017) e00367]. Heliyon.

[40] Emms D. M., Kelly S. (2019). OrthoFinder: Phylogenetic
orthology inference for comparative genomics. Genome Biol..

[41] Dyer S. C., Austine-Orimoloye O., Azov A. G., Barba M., Barnes I., Barrera-Enriquez V. P., Becker A., Bennett R., Beracochea M., Berry A. (2025). Ensembl 2025. Nucleic Acids Res..

[42] Goldfarb T., Kodali V. K., Pujar S., Brover V., Robbertse B., Farrell C. M., Oh D. H., Astashyn A., Ermolaeva O., Haddad D., Hlavina W., Hoffman J., Jackson J. D., Joardar V. S., Kristensen D., Masterson P., McGarvey K. M., McVeigh R., Mozes E., Murphy M. R., Schafer S. S., Souvorov A., Spurrier B., Strope P. K., Sun H., Vatsan A. R., Wallin C., Webb D., Brister J. R., Hatcher E., Kimchi A., Klimke W., Marchler-Bauer A., Pruitt K. D., Thibaud-Nissen F., Murphy T. D. (2025). NCBI RefSeq: Reference
sequence standards through 25 years of curation and annotation. Nucleic Acids Res..

[43] US FDA Phish-Pharm: A searchable database of pharmacokinetics and drug residue literature in fish; US FDA, 2024, (Accessed May 2025, https://www.fda.gov/animal-veterinary/tools-resources/phish-pharm. 10.1208/s12248-022-00750-w 36195686

[44] Berninger J. P., La Lone C. A., Villeneuve D. L., Ankley G. T. (2015). Prioritization of
pharmaceuticals for potential environmental hazard through leveraging
a large-scale mammalian pharmacological dataset. Environ. Toxicol. Chem..

[45] Krumpholz L., Klimczyk A., Bieniek W., Polak S., Wiśniowska B. (2024). Data set of
fraction unbound values in the *in vitro* incubations
for metabolic studies for better prediction of human clearance. Database.

[46] DrugBank (2025) Database for drug and drug target information; (accessed May 2025), https://go.drugbank.com/.

[47] Koprulu M., Wheeler E., Kerrison N. D., Denaxas S., Carrasco-Zanini J., Orkin C. M., Hemingway H., Wareham N. J., Pietzner M., Langenberg C. (2025). Sex differences
in the genetic regulation of the human
plasma proteome. Nat. Commun..

[48] OECD. Test No. 210: fish, Early-life Stage Toxicity Test, OECD Guidelines for the Testing of Chemicals, Section 2; OECD Publishing: Paris, 2013. 10.1787/9789264203785-en.

[49] OECD. Test No. 215: fish, Juvenile Growth Test, OECD Guidelines for the Testing of Chemicals, Section 2; OECD Publishing: Paris, 2000. 10.1787/9789264070202-en.

[50] Davison W. G., Cooper C. A., Sloman K. A., Wilson R. W. (2023). A method for measuring
meaningful physiological variables in fish blood without surgical
cannulation. Sci. Rep..

[51] Claiborne, J. B. Acid-base regulation.In The Physiology of Fishes; 2nd Evans, D. H. , eds.;1998, CRC: Boca Raton, FL, pp. 177–198.

[52] Gilderhus P. A. (1989). Efficacy
of Benzocaine as an Anesthetic for Sahnonid Fishes. N. Am. J. Fish. Manag..

[53] HM Government Animals (Scientific Procedures) Act 1986; 2025, (Accessed October 2025). https://www.legislation.gov.uk/ukpga/1986/14/contents.

[54] Thermo-Fisher (2024) Pierce BCA Protein Assay Kit User Guide; (Accessed May 2025), https://assets.thermofisher.com/TFS-Assets/LSG/manuals/MAN0011430_Pierce_BCA_Protein_Asy_UG.pdf.

[55] Bianchi-Bosisio, A. Proteins: Physiological Samples. In Encyclopedia of Analytical Science (Second ed.), Paul, Worsfold ; Alan, Townshend ; Colin, Poole , Eds.; Elsevier, 2005; pp. 357–375. 10.1016/B0-12-369397-7/00494-5.

[56] Sigma-Aldridge (2025) BCP (Bromocresol Purple) Albumin Assay Kit Catalog Number MAK125 Technical Bulletin; (Accessed May 2025): https://www.sigmaaldrich.com/deepweb/assets/sigmaaldrich/product/documents/382/812/mak125bul.pdf?srsltid=AfmBOooUEzKPmNP7175V7sz69r7bZlOvnW6jpcFTKsUiVYjrnYhLgsal.

[57] Smedes F. (1999). Determination
of total lipid using non-chlorinated solvents. The Anal..

[58] Thermo-Fisher (2012) The Thermo Scientific RED Device Systems Brochure; (Accessed October 2025): https://documents.thermofisher.com/TFS-Assets/LSG/brochures/1602445-Equilibrium-Dialysis-Brochure.pdf.

[59] HT Dialysis (2025) Reusable 96-well Equilibrium Dialysis Devices; (Accessed October 2025): https://www.htdialysis.com/.

[60] Palmgrén J. J., Mönkkönen J., Korjamo T., Hassinen A., Auriola S. (2006). Drug adsorption to
plastic containers and retention
of drugs in cultured cells under in vitro conditions. Eur. J. Pharm. Biopharm..

[61] Pihlaja, T. ; Brown, A. R. ; Sikanen, T. (in press). Evaluation of in vitro hepatic clearances and nonspecific binding of ionisable human pharmaceuticals in rainbow trout S9 fractions and plasma and their impacts on extrapolated bioconcentration factors.

[62] Wood, S. N. Generalized Additive Models: An Introduction with R, 2nd ed.; Chapman and Hall/CRC Press: Boca Raton, FL, pp.496. 2017.

[63] R Core Team R: a Language and Environment for Statistical Computing.; R Foundation for Statistical Computing. https://www.r-project.org/, 2025.

[64] Thomas, R. J. Data Analysis with R Statistical Software: A Guidebook for Scientists; Publ. Eco-explore, 2015, http://www.eco-explore.co.uk/data-analysis-consulting/data-analysis-guidebook/.

[65] De
Smet W. H. (1978). The total protein content in the blood serum of vertebrates. Acta Zool. Pathol. Antverp.

[66] De
Smet H., Blust R., Moens L. (1998). Absence of albumin in the plasma
of the common carp *Cyprinus carpio*: Binding of fatty
acids to high density lipoprotein. Fish Physiol.
Biochem..

[67] Blanco, A. ; Gustavo Blanco, G. Chapter 3 - Proteins, in Ed(s): Antonio Blanco. InMedical Biochemistry; Gustavo, Blanco Eds.; Second ed.; Academic Press, 2022, pp. 21–75. 10.1016/B978-0-323-91599-1.00004-3.

[68] Cameron, J. M. ; Bruno, C. ; Parachalil, D. R. ; Baker, M. J. ; Bonnier, F. ; Butler, H. J. ; Byrne, H. J. Chapter 10 - Vibrational spectroscopic analysis and quantification of proteins in human blood plasma and serum. In Wood, Vibrational Spectroscopy in Protein Research; Yukihiro, Ozaki ; Malgorzata, Baranska ; Igor, K. ; Lednev; Bayden, R. , Eds.; Academic Press, 2020; pp. 269–314. 10.1016/B978-0-12-818610-7.00010-4.

[69] BioIVT (2025) Red blood cells; (Accessed June 2025), https://bioivt.com/biofluids-blood-derived/red-blood-cells-biofluids-blood-derived.

[70] Tripathi N. K., Latimer K. S., Burnley V. V. (2004). Hematologic
reference intervals for
koi (Cyprinus carpio), including blood cell morphology, cytochemistry,
and ultrastructure. Veterinary Clinical Pathol..

[71] Di L., Umland J. P., Trapa P. E., Maurer T. S. (2012). Impact of recovery
on fraction unbound using equilibrium dialysis. J. Pharm. Sci..

[72] Michorowska S. (2021). Ataluren-promising
therapeutic premature termination codon readthrough frontrunner. Pharmaceuticals.

[73] Liu N., Song W., Song T., Fang L. (2016). Design and evaluation
of a novel felbinac transdermal patch: Combining ion-pair and chemical
enhancer strategy. AAPS Pharm. Sci. Technol..

[74] Dencker S. J., Johansson R., Malm U. (1988). Pharmacokinetic and pharmacodynamic
studies on high doses of fluphenazine. Psychopharmacology.

[75] UK Government Levomprozamine 5 mg/mL oral solution: 2.5 Clinical overview; 2025, (Accessed September 2025), https://assets.publishing.service.gov.uk/media/65f2338a133c221271cd38e4/FOI_23-753_PDF_attachment__1_.pdf.

[76] Uang Y. S., Chen I.-K., Wang L.-H., Hsu K.-Y. (2001). Determination of
mephenoxalone in human plasma sample by high-performance liquid chromatography–fluorescence
detection. J. Chromatogr. B: biomed. Sci. Appl..

[77] University Hospital Sussex Mycophenolic acid; University Hospital Sussex, 2025, (Accessed September 2025), https://pathology.uhsussex.nhs.uk/pug/biochemistry-immunology/biochemistry-tests/405-mycophenolic-acid.

[78] Rolfo C., Isambert N., Italiano A., Molife L. R., Schellens J. H. M., Blay J.-Y., Decaens T., Kristeleit R., Rosmorduc O., Demlova R., Lee M.-A., Ravaud A., Kopeckova K., Learoyd M., Bannister W., Locker G., de Vos-Geelen J. (2020). Pharmacokinetics and safety of olaparib
in patients with advanced solid tumours and mild or moderate hepatic
impairment. Br. J. Clin. Pharmacol..

[79] Breyer-Pfaff U., Nill K., Schied H. W., Gaertner H. J., Giedke H. (1988). Single-dose
kinetics of the neuroleptic drug perazine in psychotic patients. Psychopharmacology.

[80] Quasthoff S., Möckel C., Zieglgänsberger W., Schreibmayer W. (2008). Tolperisone:
A typical representative of a class of centrally acting muscle relaxants
with less sedative side effects. CNS Neurosci
Ther..

[81] Straub J. O., Oldenkamp R., Pfister T., Häner A. (2019). Environmental
risk assessment for the active pharmaceutical ingredient mycophenolic
acid in European surface waters. Environ. Toxicol.
Chem..

[82] Memmert U., Peither A., Burri R., Weber K., Schmidt T., Sumpter J. P., Hartmann A. (2013). Diclofenac: New data on chronic toxicity
and bioconcentration in fish. Environ. Toxicol.
Chem..

[83] Overturf M. D., Overturf C. L., Baxter D., Hala D. N., Constantine L., Venables B., Huggett D.B. (2012). Early life-stage toxicity of eight
pharmaceuticals to the fathead minnow, *Pimephales promelas*. Arch. Environ. Contam. Toxicol..

[84] Astrazeneca Environmental Risk Assessment Data: Olaparib, Version 3 – April 2024; Astrazeneca, 2024, (Accessed February 2026): https://www.astrazeneca.com/content/dam/az/PDF/Sustainability/era/Olaparib.pdf.

[85] Astrazeneca Environmental Risk Assessment Data: Quetiapine fumarate, Version 2 – October 2023; 2023, Astrazeneca, (Accessed February 2026): https://www.astrazeneca.com/content/dam/az/PDF/Sustainability/era/Quetiapine-fumarate.pdf.

[86] Ankley G. T., Jensen K. M., Kahl M. D., Makynen E. A., Blake L. S., Greene K. J., Johnson R. D., Villeneuve D. L. (2007). Ketoconazole
in the fathead minnow (*Pimephales promelas*): Reproductive
toxicity and biological compensation. Environ.
Toxicol. Chem..

[87] Fellows, F. C. ; Hird, F. J. R. Fatty acid binding proteins in the 87) AstraZeneca (2023). Environmental Risk Assessment Data: Zolmitriptan, Version 2– November 2023; Astrazeneca, 1981, (Accessed February 2026): https://www.astrazeneca.com/content/dam/az/PDF/Sustainability/era/Zolmitriptan.pdf.

[88] Sehonova P., Plhalova L., Blahova J., Doubkova V., Marsalek P., Prokes M., Tichy F., Skladana M., Fiorino E., Mikula P. (2107). Effects
of selected tricyclic antidepressants on early-life
stages of common carp (*Cyprinus carpio*). Chemosphere.

[89] European Medicines Agency Guideline On The Environmental Risk Assessment Of Medicinal Products For Human Use; European Medicines Agency, Committee For Medicinal Products For Human Use (CHMP), 01 June 2006, EMEA/CHMP/SWP/4447/00 Corr 21. 2006.

[90] European Medicines Agency Guideline On The Environmental Risk Assessment Of Medicinal Products For Human Use; European Medicines Agency, Committee For Medicinal Products For Human Use (CHMP), 22 August 2024, EMEA/CHMP/SWP/4447/00 Rev. 1- Corr. 2024.

[91] Colclough N., Ruston L., Wood J. M., MacFaul P. A. (2014). Species differences
in drug plasma protein binding. Med. Chem. Comm..

[92] Bardal, S. K. ; Waechter, J. E. ; Martin, D. S. Chapter 2 - Pharmacokinetics. InApplied Pharmacology; Elsevier, 2011, Stan, K. ; Bardal; Jason, E. ; Waechter; Douglas, S. eds.; pp.17–34. 10.1016/B978-1-4377-0310-8.00002-6

[93] Fellows F. C. I., Hird F. J. R. (1981). Fatty acid binding
proteins in the serum of various
animals. Ibid.

[94] Yanagisawa T., Hashimoto K., Matsuura F. (1977). Occurrence of multiple
albumins in
carp blood plasma. Bull. Japan. Soc. i. Fish.

[95] Williams, D. A. ; Lemke, T. L. Foye’s Principles of Medicinal Chemistry; Lippincott williams, 2008, 1070–1079.

[96] Manallack D. T. (2007). The pK­(a)
distribution of drugs: Application to drug discovery. Perspect Medicin Chem..

[97] Zhou H., Adedoyin A., Wilkinson G. R. (1990). Differences
in plasma binding of
drugs between Caucasians and Chinese subjects. Clin. Pharmacol. Ther..

[98] Routelege P. A. (1986). The plasma
protein binding of basic drugs. Br. J. Clin.
Pharmacol..

[99] Adir J., Miller A. K., Vestal R. E. (1982). Effects
of total plasma concentration and age on tolbutamide plasma protein
binding. Clin. Pharmacol. Ther..

[100] Hirata K., Ikeda T., Watanabe H., Maruyama T., Tanaka M., Chuang V. T. G., Uchida Y., Sakurama K., Nishi K., Yamasaki K., Otagiri M. (2021). The binding of aripiprazole
to plasma proteins in chronic renal failure patients. Toxins.

[101] Jing J.-J., Liu B., Wang X., He L.-L., Guo X.-Y., Xu M.-L., Li Q.-Y., Gao B., Dong B.-Y. (2017). Binding of fluphenazine with human serum albumin in
the presence of rutin and quercetin: An evaluation of food-drug interaction
by spectroscopic techniques. Luminescence.

[102] Mc Donald, D. G. ; Milligan, C. L. 2 Chemical Properties of the Blood. In Fish Physiology, Hoar, W.S. ; Randall, D.J. ; Farrell, A.P. , Eds.; Academic Press, 1992; Vol. 12, pp. 55–133. 10.1016/S1546-5098(08)60009-6.

[103] Krause S., Goss K.U. (2021). Relevance of desorption kinetics
and permeability for in vitro-based predictions of hepatic clearance
in fish. Aquat. Toxicol..

[104] Zeitlinger M. A., Derendorf H., Mouton J. W., Cars O., Craig W. A., Andes D., Theuretzbacher U. (2011). Theuretzbacher
U. Protein binding: Do we ever learn?. Antimicrob.
Agents Chemother..

[105] Matthee C., Brown A. R., Lange A., Tyler C. R. (2023). Factors
determining the susceptibility of fish to effects of human pharmaceuticals. Environ. Sci. Technol..

