# Supplementary material for: Accounting for Differences in Plasma Protein Binding between Fish and Humans for Supporting Environmental Risk Assessment of Pharmaceuticals Using the Fish Plasma Model
Source: Environ Sci Technol. 2026 Apr 21;60(17):12748–63. doi: 10.1021/acs.est.5c15513 (PMC13151045; doi:10.1021/acs.est.5c15513)
Supplement: Supplementary file 1 [file es5c15513_si_001.pdf]

Accounting for differences in plasma protein binding between fish and humans for supporting environmental risk assessment of pharmaceuticals using the fish plasma model

Andrew Ross Brown[1*], Maciej Trznadel[1], Siffreya Pedersen[1] Alison Nimrod Perkins[2], Michael Lee[2], Michael Mohutsky[2], Alec Bell[2], Tea LM Pihlaja[3], Tiina Sikanen[3], Mirco Weil[4], Anja Coors[1,4], Rod Wilson[1], Charles R. Tyler[1]

1 Biosciences, University of Exeter, Stocker Road, Exeter, Devon, EX4 4QD, United Kingdom

2 Eli Lilly and Company, Indianapolis, Indiana 46285, United States

3 Faculty of Pharmacy, University of Helsinki, PL 56, Viikinkaari 5, Helsinki, Finland

4 ECT Oekotoxikologie GmbH, Boettgerstr. 2-14, 65439 Floersheim/Main, Germany

* Corresponding author e-mail contact: ross.brown@exeter.ac.uk

Supporting Information (Contents):

SI Table 1: The 22 most abundant proteins represent approximately 99% of the total protein mass in human blood plasma (**Pages S2-S5**)

SI Table 2: Species for which OrthoFinder was used to search for orthologues of genes that encode albuminoid proteins, AGP and apolipoproteins (**Page S6**)

SI Table 3: Protein binding attribution for the studied APIs in humans (pH 7.4) (**Page S7-S8**)

SI Table 4: Physico-chemical properties of APIs and their ionisation status at different physiological pHs in different fish species and humans (**Pages S9-S10**)

SI Table 5: Methodological details for LC-fluorescence and LC-MS/MS analysis of APIs in blood plasma samples and isotonic, pH-matched buffers (**Pages S11-S19**)

SI Table 6: Currently available fish plasma protein binding data (and supporting metadata) for APIs (**Pages S20-21**)

SI Table 7: Total protein concentration in fish blood serum (and plasma) (**Page S22**)

SI Table 8: Assessment of non-specific binding of API – measurement of percentage recovery (**Pages S23-24**)

SI Table 9: Variation between analytical laboratories in measured unbound fractions of APIs selected for intercomparison in fish and humans (**Pages S25-28**)

SI Table 10: Therapeutic water concentrations predicted for rainbow trout, koi carp, fathead minnow versus observed effect concentrations for APIs (**Pages S29-30**)

SI Table 11: Evidence of albumin-like proteins in fish blood plasma (**Page S31**)

SI Figure 1 Gene trees of the albuminoids, albumin, AFP and AFM and the GC gene (**Page S32**)

SI Figure 2 Gene tree of the ApoA1/A4/E orthogroup (**Page S33**)

SI Figure 3 Gene trees of the apolipoprotein A orthogroups (**Page S34**)

SI Figure 4 Gene tree of the apolipoprotein B orthogroup (**Page S35**)

SI Figure 5 Gene trees of the apolipoprotein C orthogroups (**Page S36**)

SI Figure 6 Gene tree of the apolipoprotein D orthogroup (**Page S37**)

SI Figure 7 Gene trees of the apolipoprotein F and H orthogroups (**Page S38**)

SI Figure 8 Gene trees of the apolipoprotein L orthogroups (**Page S39**)

SI Figure 9 Gene trees of the apolipoprotein O orthogroups (**Page S40**)

SI Figure 10 Gene trees of the apolipoprotein M and orosomucoid orthogroups (**Page S41**)

References (not included in main manuscript) (**Pages S41-44**)

SI Table 1: The 22 most abundant proteins represent approximately 99% of the total protein mass in human blood plasma (after Cameron et al., 2020)

Top 10	Top 11-22
1) Albumin	11) Apolipoprotein A1
2) IgGs	12) Apolipoprotein B
3) Transferrin	13) Acid-1-Glycoprotein
4) Fibrinogen	14) Lipoprotein (a)
5) IgAs	15) Factor H
6) Alpha-2-Macroglobulin	16) Ceruloplasmin
7) IgMs	17) Complement C4
8) Alpha-1-Antitrypsin	18) Complement Factor B
9) Complement C3	19) Prealbumin
10) Haptoglobin	20) Complement C9
	21) Complement C1q
	22) Complement C8

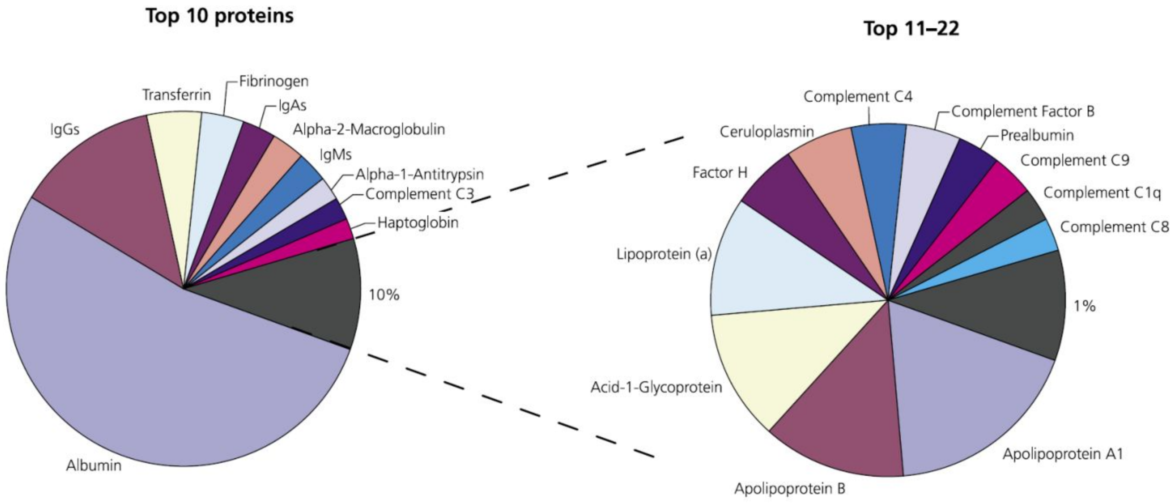

Notes:

Human blood plasma contains more than 300 proteins, but only 22 make up 99% of total plasma protein mass. Human serum albumin (HSA) is the most abundant, accounting for over 50% of total plasma proteins by mass, while globulins represent a further 40% in healthy individuals (Cameron et al., 2020). Albumin is largely responsible for binding anionic/acidic APIs, while the immune-globulin alpha(1)-acid glycoprotein (AGP) is largely responsible for binding cationic/basic APIs (see below). API ligand binding capacity depends on the maximum amount of ligand that binding sites can accommodate and the total number of binding sites available, which in turn depends on plasma protein concentration (Bordbar et al., 2004). Total protein concentrations depend on hydration, nutrition and immune status and range from 65 to 86 g/L in human blood plasma, or 60 to 80 g/L in

human blood serum (excluding clotting proteins, which comprise 8% of total proteins) (Cameron et al., 2020; Blanco and Blanco, 2022).

Plasma protein concentrations in different fish species range between 20 and 80 g/L (**SI Table 5**) and vary with age, nutritional status, genetics and environmental factors such as pH, temperature and food availability (reviewed in McDonald and Milligan, 1992). A similar range of plasma protein concentrations has been recorded across other major taxonomic groups of animals (De Smet, 1978; Enerstvedt et al., 2017).

Plasma proteins in fish resemble those in humans (and mammalian models), including: albumin-like proteins; immunoglobulins (e.g. AGP-like proteins); blood clotting proteins (e.g. fibrinogen and prothrombin); apolipoproteins (e.g. APOA-1-like proteins); hormone binding proteins; Ca^{2+} binding proteins (e.g. vitellogenin); and metal binding proteins (e.g. transferrin) (reviewed in McDonald and Milligan, 1992). Based on data for humans and mammalian models from (pre)clinical studies, plasma proteins which are most likely to responsible for binding APIs in fish are summarised below.

Albumin

Albumins play a key role in maintaining osmotic pressure/blood volume, as well as binding and transporting exogenous and endogenous ligands. Human serum albumin constitutes ~60% of total serum proteins (35-50 g/L), while other human albumins include alpha-fetoprotein (AFP) expressed in the foetus, afamin (AFM) a vitamin E binding protein and group specific component (GC also known as vitamin D binding protein -DBP) (Bteich, 2019; Andreeva, 2022). HSA is a monomeric, heart shaped protein with three homologous domains (I-III), each featuring six to four α-helices, all of which comprise a total of 585 amino acids with a combined molecular weight (MW) of 66.5 kDa (Curry et al., 1998). HSA has seven ligand binding sites, three of which are major binding sites for APIs. Sudlow drug site DS1 contains a number of positively charged amino acid residues including $Lys192^{+}$, $Lys196^{+}$, $Arg215^{+}$, and $Arg254^{+}$ (Yildiz, 2024) and has a preferential binding affinity for bulky, heterocyclic, anionic APIs such as azapropazone, phentylbutazone and warfarin (Sudlow et al., 1975). DS2 mainly comprises hydrophobic nonpolar amino acid residues such as Leu387, Phe403, Leu407, Val433, Ala449 and aromatic Tyr411, (Yildiz, 2024) and preferentially binds aromatic, compounds such as anionic ibuprofen and cationic/basic APIs, including diazepam (Sudlow et al., 1975) and propranolol (Ravis et al., 1988). DS3 was discovered more recently to be another major binding site for acidic, neutral, and basic APIs (e.g. anti-cancer drugs - camptothecin, teniposide, suramin, anticoagulants - dicoumarol, nonsteroidal anti-inflammatory drugs) (Zsila, 2013).

Albumin-like proteins (acidic palmitate-binding proteins with molecular weight 68-75 kDa) have been shown to be present in diverse fish species, including jawless fish (Agnatha), cartilaginous fish (Chondrichthyes*) and bony fish (Osteichthyes) (reviewed in Andreeva, 2010; Enerstvedt et al., 2017). Importantly, albumins in fish blood plasma may be subject to intense catabolism during prolonged periods of starvation and may be far more dynamic than mammalian albumins, with shorter half-lives and varying monomeric and oligomeric structures (Andreeva, 2010). Additionally, the finding of sialized albumin-like proteins in fish (and amphibians) suggests that remaining fish albumins were initially glycoproteins, but lost sialic acid subunits, probably during evolutionary separation of fish and amphibians from reptiles (Metcalf et al., 1998). Albumin-like proteins have been identified in at least 10 ray finned fish species, including eight salmonid species (i.e. Atlantic salmon - *Salmo salar,* brown trout - *Salmo trutta*, Rainbow trout - *Oncorhynchus mykiss*, Coho salmon - *Oncorhynchus kisutch*, Pink salmon - *Oncorhynchus gorbuscha*, Chinook slamon - *Oncorhynchus tshawyfscha*, Brook trout - *Salvelinus fontinalis* and Artic charr - *Salvelinus alpinus*), plus Australian lungfish (*Neoceratodus*

forsteri) and Northern pike (*Esox lucius*) and 2 jawless fish species. Sea lamprey (*Petromyzon marinus*) and American brook lamprey (*Lethenteron appendix*) (**SI Table 7**).

Albumin-like genes (with orthologous amino acid sequences to HSA) have been identified fish, including two genes (*alb1* and *alb2*) in rainbow trout (*Oncorhynchus mykiss*) (Gong, 1997; Cairns et al., 2008) and in Atlantic salmon (*Salmo salar*) (Byrnes and Gannon, 1990). Conversely, orthologs for HSA have been shown to be absent in the zebrafish (*Danio rerio*) (Noel et al., 2010), common carp (*Cyprinus carpio*) (De Smet et al., 1998), Antarctic toothfish (*Dissostichus mawsoni*) (Metcalf et al., 1999) and cartilaginous fish (Metcalf and Gemmel, 2005). Nevertheless, albumin-like genes have been detected zebrafish and carp in the form of the *gc* gene for vitamin D binding protein (Noel et al., 2010; De Smet et al., 1998).

Alpha(1)-acid glycoprotein

Alpha(1)-acid glycoprotein (AGP, also referred to as AAG or orosomucoid – ORM1) is an acute phase protein, which can increase in concentration in plasma by 2 to 6-fold in humans and 2 to 20-fold in other mammalian species in response to inflammation, infection, and cancer (Smith and Waters, 2018) from a basal blood plasma concentration of around 0.3 g/L (3-5% of total plasma proteins) in most of these species (Ceciliani and Lecchi, 2019). AGP has a single (monomeric) polypeptide chain consisting of 183 amino acids in humans and 183–194 amino in other mammals (MW 41–44 kDa) (Smith and Waters, 2018), with five glycan chains bearing sialic acid groups. These groups are negatively charged (Smith and Waters, 2018), attracting basic/cationic APIs, which ultimately bind to negatively charged amino acid residues such as Asp^- and Glu^- in the peptide chain (Bteich, 2019). Two high affinity binding sites are available for basic/cationic APIs, while there is only one binding site for acidic/anionic APIs (Schley and Mueller-Oerlinghausen, 1986), and up to 7 sites for steroids (Kerkay and Westphal, 1968). These binding sites have also been characterized across other mammalian species; cows and dogs lack the acidic API binding site (Matsumoto et al., 2002). Although binding affinity is generally high, the binding capacity of AGP is low, owing to rapid saturation of binding sites in the small glycoprotein; therefore, the fraction of unbound API (f_u) can be high in plasma when their concentrations are elevated. Furthermore, loss of sialic acid groups (desialylation during infection) and lowering of plasma pH have been shown to decrease API binding by AGP (Ponganis and Stanski, 1985; Wong and Hsia, 1983; Ceciliani and Lecchi, 2019).

Homologous amino acid sequences for human AGP appear to be largely absent in fish, with a few notable exceptions, including rainbow trout (Cairns et al., 2008), Atlantic killifish (*Fundulus heteroclitus*) and Pacific surfperch (*Neoditrema ransonnetii*) (Ceciliani and Lecchi, 2019). Other AGP-like proteins have been detected in the blood plasma of several divergent fish species and have been shown to bind cationic ligands, including APIs such as triclosan (Nassef et al., 2011). These include tributyltin-binding protein type 1 (TBT-bp1) found in the blood plasma of Japanese flounder (*Paralichthys olivaceus*) (Shimasaki et al., 2002) and Japanese medaka (*Oryzias latipes*) (Nassef et al., 2011). TBT-bp type 2 (TBT-bp2) has also been detected in Japanese flounder (Oba et al., 2007), nrF-AGP in Pacific surfperch (Nakamura et al., 2009), and AGP-like male-specific protein in mango tilapia (*Sarotherodon galilaeus*) (Machnes et al., 2008), Pacific surfperch (Nakamura et al., 2009) and Japanese flounder (Oba et al., 2007).

Lipoproteins including HDL, LDL, VLDL, and chylomicrons

Neutral and hydrophobic APIs can also bind to plasma lipoproteins (HDL, LDL and VLDL), which combine apolipoproteins and lipids. Fish contain a relatively high lipoprotein concentration (mainly HDL) in their plasma and in rainbow trout for example, apolipoproteins account for 36% of total

protein, compared with only 10% in humans (Babin and Vernier, 1989). In humans, there are several apoliprotein sub-families (Apo- A, B, C, D, E, L, F, H, M, N, and R), which have various immune-modulatory (acute phase) functions and can dramatically increase in concentration in blood plasma, serving as markers for a variety of cancers and neurological and other diseases (Liu et al., 2021). The major apolipoproteins in human HDL are apolipoprotein A-I (ApoA-I) and apolipoprotein A-2 (ApoA-II) (Amthauer et al., 1989). HDL in rainbow trout comprises five apolipoproteins (in order of abundance) ApoA-I-like and ApoA-II-like, APOB and APOC (Babin, 1987) and APOEb (Bakke et al., 2020), while carp HDL contains only ApoA-I-like and ApoA-II-like (Amthauer et al., 1989) and these lipoproteins have been shown to bind palmitate, evidencing albumin-like properties (Nakagawa et al., 1976). The dominant apolipoprotein in zebrafish plasma is ApoA-I-like (Li et al., 2016). ApoE and ApoM genes have also been identified in zebrafish and puffer fish (*Tetraodon nigroviridis*), suggesting conserved biological roles for some of these various lipoprotein systems in fish (Van Dijk et al., 2006).

SI Table 2: Species for which OrthoFinder was used to search for orthologues of genes that encode albuminoid proteins, AGP and apolipoproteins

Class	Order	Family	Species	Genome assembly
**Teleostei**	Osteoglossiformes	Mormyridae	*Paramormyrops kingsleyae*	PKINGS_0.1
		Osteoglossidae	*Scleropages formosus*	fSclFor1.1
	Siluriformes	Ictaluridae	*Ictalurus punctatus*	ASM400665v3
	Gymnotiformes	Gymnotidae	*Electrophorus electricus*	fEleEle1.pri
	Cypriniformes	Cyprinidae	*Pimephales promelas*	GCF_016745375.1
			Cyprinus carpio	Cypcar_WagV4.0
		Danionidae	*Danio rerio*	GRCz11
	Perciformes	Osphronemidae	*Betta splendens*	fBetSpl5.2
		Cichlidae	*Amphilophus citrinellus*	Midas_v5
	Beryciformes	Holocentridae	*Myripristis murdjan*	fMyrMur1.1
	Esociformes	Esocidae	*Esox lucius*	fEsoLuc1.pri
	Salmoniformes	Salmonidae	*Salmo salar*	Ssal_v3.1
			Salmo trutta	fSalTru1.1
			Oncorhynchus mykiss	USDA_OmykA_1.1
			Oncorhynchus tshawytscha	Otsh_v2.0
			Oncorhynchus kisutch	Okis_V2
**Holocephali**	Chimaeriformes	Callorhinchidae	*Callorhinchus milii*	Callorhinchus_milii-6.1.3
**Amphibia**	Anura	Pipidae	*Xenopus tropicalis*	UCB_Xtro_10.0
**Aves**	Galliformes	Phasianidae	*Gallus gallus*	bGalGal1.mat.broiler.GRCg7b
	Passeriformes	Thraupidae	*Camarhynchus parvulus*	Camarhynchus_parvulus_V1.1
**Mammalia**	Rodentia	Muridae	*Mus musculus*	GRCm39
	Carnivora	Felidae	*Felis catus*	Felis_catus_9.0
	Primates	Hominidae	*Homo sapiens*	GRCh38
			Pan troglodytes	Pan_tro_3.0

SI Table 3: Protein binding attribution for study APIs in humans (pH 7.4)

Data and references underlying figure 1f in main manuscript.

API speciation: A = Anion (+); a = Anionic (unionised); N = Neutral (0); C = Cation (-); c = Cationic (unionised); Z= Zwitterion (+/-). Predominant charge was predicted from pH and pKa using Chemaxon Marvin 23.12.

Log D_{ow} (pH 7.4) values were obtained from Chemaxon, Marvin 23.12.

Speciation (predominant charge @ pH 7.4-7.9)	API name (Log Dow pH 7.4)	A - Albumin, AGP, L - Lipo-protein	References
A (-)	Meloxicam (-1.12)	A	Türck et al. (1996)
A (-)	Mycophenolic acid (-0.43)	A	Nowak and Shaw (1995)
A (-)	Ketoprofen (0.24)	A	Czub et al. (2021)
A (-)	Indomethacin (0.38)	A	Mason and McQueen (1974)
A (-)	Felbinac (0.73)	A	Zhand et al. (2011)
A (-)	Diclofenac (0.75)	A	Dutta (2006)
A (-)	Mephenoxalone (1.1)	A	Uang et al. (2001)
A (-)	Nimesulide (1.13)	A	Bernareggi A (1998)
A (-)	Ataluren (1.15)	?	-
A (-)	Tolbutamide (1.27)	A	Adire et al. (1982)
A (-)	Ibuprofen (1.31)	A	Jamali and Brocks (2015).
A (-)	Atorvastatin (2.01)	A	Haghaei et al. (2022)
A (-)	Phenylbutazone (2.26)	A	Chignell and Starkweather (1971)
A (-)	Salinomycin (4.14)	A, AGP	Resham et al (2015)
a (0)	Olaparib (1.75)	A 56% AGP 29%	Europa (2018)
a (0)	Indapamide (2.51)	A, AGP, L	Urien et al. (1988)
a (0)	Tribenoside (4.73)	A	-
N (0)	Bimatoprost (2.35)	A	Sebbag et al. (2020)
N (0)	Cilostazol (2.97)	A	Suri et al. (1998)
c (0)	Clomethiazole (1.58)	A?	Nationa et al. (1977)
c (0)	Imiquimod (2.71)	A, AGP, L	FDA (2008)
c (0)	Quetiapine (3.05)	A	Zargar et al. (2022)
c (0)	Ketoconazole (4.24)	A	Schafer-Korting et al. (1991)
C (+)	Sulpiride (-1.56)	A	Da Silva Fragoso et al. (2016)
C (+)	Zolmitriptan (-0.16)	A	CJN (2000)
C (+)	Flecainide (0.6)	AGP	Conard and Ober (1984)
C (+)	Propranolol (0.63)	AGP	Routledge (1986)
C (+)	Prazosin (0.75)	A, AGP	Dale and Nilsen (1984)
C (+)	Procyclidine (1.1)	A	Hollósy et al. (2006)
C (+)	Desloratadine (1.17)	A	Shihab-Us-Sakib et al. (2012)
C (+)	Atomoxetine (1.78)	A	Sauer et al. (2003)
C (+)	Tolperisone (1.8)	A	Rabbani et al. (2018)
C (+)	Clozapine (1.95)	AGP	Man et al. (2019)
C (+)	Levomepromazine (2.27)	A, AGP	Kandagal et al. (2008)
C (+)	Haloperidol (2.27)	A, AGP	Levinson and Levine (1995).
C (+)	Verapamil (2.36)	AGP	Routledge (1986)
C (+)	Perazine (2.54)	AGP	Brinkschulte et al. (1982)

C (+)	Orphenadrine (2.6)	A	Martínez-Gómez et al. (2007)
C (+)	Azelastine (2.66)	A	Almutairi et al. (2020)
C (+)	Clomipramine (2.72)	A	Braithwaite (1980)
C (+)	Fluphenazine (2.74)	A	Jing et al. (2017)
C (+)	Aripiprazole (3.14)	A	Sakumura et al. (2018)
C (+)	Ticlopidine (3.52)	A, AGP, L	FDA (1999)
Z (+/-)	Fexofenadine (2.48)	A, AGP	FDA (2026)

SI Table 4: Physico-chemical properties of APIs and their ionisation status at different physiological pHs in different fish species and humans

Fish species: Carp = koi carp; FHM = fathead minnow; Trout = rainbow trout. Ionisation (%) was calculated from pH and pKa using Chemaxon Marvin 23.12.

API speciation: A = Anion (+); a = Anionic (unionised); N = Neutral (0); C = Cation (-); c = Cationic (unionised); Z= Zwitterion (+/-).

Speciation (predominant charge @ pH 7.4 to 7.9)	API name	CAS number	Molecular weight (g/mol)	Log K_{ow} Chemaxon	pKa Chemaxon	Log D_{ow} @ pH 7.4 (Humans) Chemaxon	Ionization (%) @ pH 7.4 (Humans) Chemaxon	Log D_{ow} @ pH 7.7 (Carp, FHM) Chemaxon	Ionization (%) @ pH 7.7 (Carp, FHM) Chemaxon	Log D_{ow} @ pH 7.9 (Trout) Chemaxon	Ionization (%) @ pH 7.9 (Trout) Chemaxon
C (+)	Aripiprazole	129722-12-9	448.4	4.79	9.04	3.14	97.75	3.43	95.62	3.62	93.23
A (-)	Ataluren	775304-57-9	284.2	4.43	3.9	1.15	99.97	0.99	99.98	0.92	99.99
C (+)	Atomoxetine	83015-26-3	255.4	3.78	9.4	1.78	99.02	2.07	98.06	2.26	96.96
A (-)	Atorvastatin	134523-00-5	558.6	5.00	4.31	2.01	99.92	1.79	99.96	1.66	99.97
C (+)	Azelastine	58581-89-8	381.9	3.87	8.58	2.66	93.76	2.94	88.27	3.11	82.6
N (0)	Bimatoprost	155206-00-1	415.6	2.35	14.35	2.35	0	2.35	0	2.35	0
N (0)	Cilostazol	73963-72-1	369.5	2.97	14.42	2.97	0	2.97	0	2.97	0
c (0)	Clomethiazole	533-45-9	161.7	1.58	3.18	1.58	0	1.58	0	1.58	0
C (+)	Clomipramine	303-49-1	314.9	4.52	9.2	2.72	98.43	3.02	96.91	3.21	95.20
C (+)	Clozapine	5786-21-0	326.8	3.39	8.83	1.95	96.36	2.23	93.03	2.41	89.40
C (+)	Desloratadine	100643-71-8	310.8	3.85	10.13	1.17	99.73	1.44	99.59	1.63	99.39
A (-)	Diclofenac	15307-86-5	296.1	3.97	4.01	0.75	99.96	0.58	99.98	0.49	99.99
A (-)	Felbinac	5728-52-9	212.2	3.36	4.73	0.73	99.79	0.47	99.89	0.31	99.93
Z (+/-)	Fexofenadine	153439-40-8	501.7	5.68	4.07/9.21	2.48	100	2.47	100	2.47	100
C (+)	Flecainide	54143-55-4	414.3	2.80	9.62	0.6	99.40	0.89	98.80	1.08	98.12
C (+)	Fluphenazine	69-23-8	437.5	3.98	8.61	2.74	94.24	3.01	89.14	3.19	83.81
C (+)	Haloperidol	52-86-8	375.9	3.08	8.14	2.27	84.54	2.51	73.26	2.64	63.35
A (-)	Ibuprofen	15687-27-1	206.3	3.83	4.85	1.31	99.72	1.04	99.86	0.87	99.91
c (0)	Imiquimod	99011-02-6	240.3	2.71	2.89	2.71	0	2.71	0	2.71	0
a (0)	Indapamide	26807-65-8	365.8	2.51	9.35	2.51	1.11	2.5	2.18	2.5	3.40
A (-)	Indomethacin	53-86-1	357.8	3.31	4.38	0.38	99.91	0.15	99.95	0.02	99.97

c (0)	Ketoconazole	65277-42-1	531.4	4.28	6.42	4.24	9.49	4.26	5.00	4.26	3.21
A (-)	Ketoprofen	22071-15-4	254.3	3.46	4	0.24	99.96	0.07	99.98	-0.02	99.99
C (+)	Levomepromazine	60-99-1	328.5	4.29	9.42	2.27	99.06	2.56	98.14	2.75	97.08
A (-)	Meloxicam	71125-38-7	351.4	1.51	4.87	-1.12	99.68	-1.49	99.83	-1.75	99.89
A (-)	Mephenoxalone	70-07-5	223.2	1.10	12.68	1.1	100	1.1	100	1.1	100
A (-)	Mycophenolic acid	24280-93-1	320.3	3.01	3.58	-0.43	99.99	-0.54	100	-0.58	100
A (-)	Nimesulide	51803-78-2	308.3	1.94	6.4	1.13	90.96	1	95.25	0.94	96.25
a (0)	Olaparib	763113-22-0	434.5	1.75	9.96	1.75	0.27	1.75	0.54	1.75	0.86
C (+)	Orphenadrine	83-98-7	269.4	4.08	8.87	2.6	96.70	2.89	93.63	3.07	90.26
C (+)	Perazine	84-97-9	339.5	3.84	8.68	2.54	95.01	2.82	90.51	2.99	85.76
A (-)	Phenylbutazone	50-33-9	308.4	4.22	5	2.26	99.61	2.18	99.80	2.14	99.88
C (+)	Prazosin	19216-56-9	383.4	1.45	8.04	0.75	81.42	0.96	68.72	1.09	58.09
C (+)	Procyclidine	77-37-2	287.4	3.27	9.58	1.1	99.34	1.39	98.69	1.59	97.94
C (+)	Propranolol	525-66-6	259.3	2.50	9.27	0.63	98.66	0.92	97.36	1.11	95.88
c (0)	Quetiapine	111974-69-7	383.5	3.22	7.07	3.05	31.60	3.13	18.80	3.16	12.75
A (-)	Salinomycin	53003-10-4	751	7.01	4.45	4.14	99.89	3.9	99.94	3.77	99.96
C (+)	Sulpiride	15676-16-1	341.4	0.04	8.97	-1.56	97.53	-1.28	95.21	-1.09	92.62
C (+)	Ticlopidine	55142-85-3	263.8	4.03	7.76	3.52	69.44	3.7	53.24	3.8	41.81
A (-)	Tolbutamide	64-77-7	270.3	2.33	5.64	1.27	97.85	1.23	99.13	1.21	99.45
C (+)	Tolperisone	728-88-1	245.4	3.15	8.74	1.8	95.64	2.08	91.66	2.26	87.39
a (0)	Tribenoside	10310-32-4	478.6	4.73	12.36	4.73	0	4.73	0	4.73	0
C (+)	Verapamil	52-53-9	454.6	4.55	9.61	2.36	99.38	2.65	98.77	2.84	98.07
C (+)	Zolmitriptan	139264-17-8	287.4	2.00	9.57	-0.16	99.33	0.13	98.67	0.32	97.91

SI Table 5: Methodological details for LC-fluorescence and LC-MS/MS analysis of APIs in blood plasma samples and isotonic, pH-matched buffers

LC-MS/MS analysis (Lab 1)

Samples for each compound were analyzed in two runs on a Sciex TripleQuad 6500+ mass spectrometer equipped with a Turbo Ion Spray source (600°C). Analytes and internal standards were monitored in MRM mode. Labetalol and niflumic acid served as internal standards. A PAL3 autosampler was used to inject samples onto a Waters Acquity BEH 1.7µm C18 2.1 x 50mm column (40°C). The pumps used were Shimadzu LC-40D x3 units. Analyte and internal standard areas were integrated using SciexOS MultiQuant software. Pharmaceutical plasma protein binding was measured in 2 experiments.

For both analytical runs and all analytes, chromatography conditions were:

**Mobile Phase Components**	**Time (min)**	**Flow (mL/min)**	**%B**
Aqueous Phase (A): 0.1% formic acid in Honeywell Water	0.00	1	1
	0.21	1	1
	0.69	1	99
Organic Phase (B): 0.1% formic acid in Fisher Acetonitrile	1.09	1	99
	1.1	1	1
	1.25	1	1
**Injection Volume (µL)**	2		

SI Table 5 (cont.)

Lab 1 Analytical run 1: A 6-point standard curve of peak area ratios (analyte to internal standard) was created for each analyte and used to fit a linear regression with $1/x^2$ weighting. The standard curve points were 1, 5, 10, 100, 500, and 1000nM.

COMPOUND	COMPOUND	Retention Time	HUMAN			TROUT			MINNOW			CARP		
ID	TRANSITION	(min)	LOQ	Linear Regression	R^2	LOQ	Linear Regression	R^2	LOQ	Linear Regression	R^2	LOQ	Linear Regression	R^2
Clomipramine	315.1 / 86.1	0.725	1nM	y=0.0009977x + 0.00304	0.996	1nM	y=0.00168x + 0.0009818	0.995	1nM	y=0.00203x + 0.0005366	0.996	1nM	y=0.00126x + 0.0003079	0.991
Verapamil	455.4 / 165.2	0.727	1nM	y=0.0006793x + 0.0002581	0.981	1nM	y=0.0006468x + 0.0002154	0.997	1nM	y=0.0006532x + 0.0002847	0.991	1nM	y=0.0009229x + 0.0001186	0.989
Aripiprazole	448.1 / 285.1	0.712	1nM	y=0.00403x - 0.0001005	0.989	1nM	y=0.00480x + 0.0007849	0.988	1nM	y=0.00401x + 0.0005462	0.984	1nM	y=0.00475x + 0.0006963	0.992
Clomethiazole	162.0 / 113.0	0.736	1nM	y=0.00126x + 0.0002315	0.991	1nM	y=0.0007431x + 0.0004229	0.997	1nM	y=0.00108x + 0.0003445	0.990	1nM	y=0.0008282x + 0.0001202	0.990
Clozapine	327.1 / 270.1	0.699	1nM	y=0.00202x + 0.0001446	0.993	5nM	y=0.00137x + 0.00235	0.977	1nM	y=0.00261x + 0.0006013	0.998	1nM	y=0.0008174x + 0.0002336	0.986
Diclofenac	296.0 / 214.1	0.809				1nM	y=0.0007664x + 0.0003043	0.993	1nM	y=0.00105x + 0.0002589	0.990	1nM	0.0007845x + 0.0001763	0.986
Flecainide	415.1 / 301.0	0.711	1nM	y=0.00637x + 0.00107	0.992	1nM	y=0.00374x + 0.00198	0.981	1nM	y=0.00544x + 0.00156	0.990	1nM	y=0.00472x + 0.0007405	0.986
Haloperidol	376.1 / 165.1	0.716	1nM	y=0.01083x + 0.00142	0.991	1nM	y=0.00552x + 0.0004239	0.992	1nM	y=0.00764x + 0.00140	0.992	1nM	y=0.00599x + 0.0009404	0.989
Olaparib	435.2 / 367.1	0.722	1nM	y=0.00287x - 0.000346	0.997	1nM	y=0.00290x + 0.0004918	0.998	1nM	y=0.00278x + 0.0003937	0.998	1nM	y=0.00252x + 0.0015	0.993
Orphenadrine	270.2 / 181.1	0.712	1nM	y=0.00635x + 0.00128	0.991	1nM	y=0.00445x + 0.0005481	0.984	1nM	y=0.00556x + 0.0005913	0.995	1nM	y=0.00458x + 0.00126	0.988
Tolperisone	246.2 / 98.1	0.7	1nM	y=0.00467x + 0.0008834	0.992	1nM	y=0.00419x + 0.0006287	0.991	1nM	y=0.00580x + 0.00152	0.997	1nM	y=0.00642x + 0.00162	0.994

SI Table 5 (cont.)

Lab 1 Analytical run 2: Due to limited fish plasma, fraction unbound was determined by comparing the peak area ratios (analyte to internal standard) in the buffer vs plasma sides. That is, concentration was not determined. This method depends on an assumption of linearity of the analyte response which has been determined in this laboratory to be a good assumption for pharmaceutical compounds due in part to the high linear range of the Sciex 6500 instrument and the low initial concentration of the analyte (500 nM). Additionally, there were 18 overlapping analyses between Experiment 1 and Experiment 2 and for 11 of those, the %CV between the average fraction unbound was ≤16%. Six had %CV between >16 and 32% and those 6 all had low fraction unbound (<0.05). The results from these 17 overlapping analyses support the fraction unbound determination method used in experiment 2. One additional overlapping analysis for olaparib had poor reproducibility in experiment 2 and was not used for evaluation.

Compound ID	Retention Time (min)	Transition
Mycophenolic acid	0.813	321.1 / 207.0
Indomethacin	0.865	358.1 / 139.1
Propranolol	0.75	260.1 / 183.1
Ataluren	0.852	285.1 / 123.0
Nimesulide	0.831	306.9 / 228.9
Tolbutamide	0.822	271.0 / 91.0
Desloratadine	0.73	311.0 / 259.2
Clomethiazole	0.811	162.0 / 113.0
Olaparib	0.768	435.2 / 367.1
Tolperisone	0.772	246.2 / 98.1

Compound ID	Retention Time (min)	Transition
Levomepromazine	0.78	329.1 / 100.1
Atorvastatin	0.844	559.4 / 440.3
Azelastine	0.769	382.3 / 112.1
Bimatoprost	0.79	398.2 / 362.2
Indapamide	0.814	365.9 / 132.1
Quetiapine	0.764	384.0 / 253.1
Imiquimod	0.745	241.1 / 185.1
Verapamil	0.77	455.4 / 165.2
Haloperidol	0.769	376.1 / 165.1
Clomipramine	0.777	315.1 / 86.1

SI Table 5 (cont.)

LC-MS/MS analysis (Lab 2)

Samples were analyzed on a TSQ Vantage triple quadrupole mass spectrometer equipped with heated electrospray (HESI II) source coupled to Surveyor MS Pump Plus HPLC pump with HTC PAL autosampler (all Thermo Fisher Scientific, San Jose, CA).

All compounds were separated using reversed-phase, 3 µm particle size, C18 Hypersil GOLD column 50 mm × 2.1 mm i.d. (Thermo Scientific, San Jose CA, USA) using a linear gradient of water and organic solvents (below). Analytical runs were 3.5 min, flow rate 0.5 mL/min. The initial composition of mobile phase is listed in the table. The gradient consists of linear increase of organic solvent to 100% in 1.5 min and it is maintained for another 1.5 min before returning to the starting condition. Temperature of autosampler was set at 6˚C, while column was kept at a room temperature.

**Compound ID**	**Aqueous Phase (A):**	**Organic Phase (B):**	**Initial composition**	**Retention time [min]**
Ticlopidine	Water +0.1% NH_3 aq	ACN +0.1% NH3 aq	30% B	2.10
Ibuprofen	5 mM Ammonium Acetate	5 mM Ammonium Acaetatein ACN	20% B	1.10
Propranolol	Water +0.1% Formic Acid	Methanol +0.1% Formic Acid	30% B	2.07
Mycophenolic Acid	Water +0.1% Formic Acid	Methanol +0.1% Formic Acid	20% B	1.62
Quetiapine	Water +0.1% Formic Acid	Methanol +0.1% Formic Acid	20% B	1.50
Olaparib	Water +0.1% Formic Acid	Methanol +0.1% Formic Acid	20% B	1.57

HESI probe was operating in positive mode with an ion-spray voltage of 3.75 kV in positive mode and 4.0 kV in negative. The heated capillary temperature was set at 275 °C and the vaporizer temperature was 350 °C. Nitrogen was employed as sheath and auxiliary gas at a pressure of 60 and 2 arbitrary units, respectively. The argon CID gas was used at a pressure of 1.5 mTorr and the optimum collision energy (CE) for each transition was automatically optimized by the software:

**Compound ID**	Aqueous Phase (A):	Organic Phase (B):	**Initial conditions**	**Collision Energy**
Ticlopidine	2.01	+	268.1-129.1	33
			264.1-125.1	32
Ibuprofen	1.10	-	205.1-161.4	5
			208.1-164.2	11
Propranolol	2.07	+	260.2-116.2	17
			267.2-189.2	19
Mycophenolic Acid	1.62	+	321.1-207.1	22
			324.2-210.1	23
Quetiapine	1.50	+	384.2- 253.1	22
			388.2- 221.0	35
Olaparib	1.57	+	435.2-281.0	31
			443.3-375.2	21

SI Table 5 (cont.)

LC-MS/MS analysis (Lab 2)

Quantification of the analytes was performed using characteristic multiple reaction monitoring (MRM) transitions listed in table with internal standard method (stable-labeled Internal standards). Calibration curves for samples were prepared using 9 matrix matched standards with concentration ranging from 20uM to 78nM (LLOQ) for plasma samples and from 5.0 uM to 9.8 nM (LLOQ) for HEPES samples. Quadratic fitting with 1/x weighing was applied. All calibration curves had R^2 greater than 0.999, both: standards and QC samples analysed during the run had calculated concentrations of less than 15% of difference from nominal concentration.

SI Table 5 (cont.)

LC-UV/vis/fluorescence analysis (Lab 3):

Agilent 1260 Infinity II with DAD (G7115A) and fluorescence (G7121B) detectors

Separation column: InfinityLab Poroshell C18 (4.6 x 100 mm, 2.7 µm)

**Test substance**	**Ataluren**		**Atorvastatin**		**Azelastine**	
Detection wavelength(s)	ex 245 nm / em 333 nm		ex 260 nm/ em 392 nm		ex 228 nm/ em 371 nm	
Solvent gradients	Time (min)	%B	Time (min)	%B	Time (min)	%B
Eluent A: 0.1% formic acid (aq.)<br>Eluent B: 0.1% formic acid in methanol	0.00<br>6.00<br>6.50	60<br>100<br>60	0<br>5<br>10<br>12<br>13<br>13.5	10<br>60<br>80<br>100<br>100<br>10	0<br>5<br>10<br>12<br>13<br>13.5<br>16	10<br>60<br>80<br>100<br>100<br>10<br>10
tR (min),<br>CV (n)	4.3,<br>0.38% (45)		11.0,<br>0.08% (45)		7.6,<br>0.14% (45)	
Vinj (µL)	10		50		50	
Linearity	y=0.010286x<br>- 0.123594		y=0.122846x<br>- 0.303606		Y=0.070717x<br>- 0.387761	
R^2	0.999887		0.999713		0.999965	
Detection range (nM)	25-2000		25-2000		25-2000	

**Test substance**	**Felbinac**		**Sulpiride**			
Detection wavelength(s)	ex 258 nm/ em 324 nm		ex 228 nm/ em 357 nm			
Solvent gradients	Time (min)	Time (min)	%B	%B		
Eluent A: 0.1% formic acid (aq.)<br>Eluent B: 0.1% formic acid in methanol	0<br>6<br>6.5<br>9	0<br>5<br>10<br>12<br>13<br>13.5	10<br>60<br>80<br>100<br>100<br>10	60<br>100<br>60<br>60		
tR (min),<br>CV (n)	3.5,<br>0.89% (45)		2.7,<br>0.12% (60)			
Vinj (µL)	10		50			
Linearity	Y=0.088728x<br>+ 3.834898		Y=0.008483x<br>- 0.151965			
R^2	0.903252		0.999894			
Detection range (nM)	50-2000		25-2000			

SI Table 5 (cont.)

LC-UV/vis/fluorescence analysis (Lab 3): Agilent 1200 with DAD (G1315C) and fluorescence (G1321A) detectors

Separation column: Agilent Poroshell 120 EC-C18 (4.6x50mm, 2.7 µm)

**Test substance**	**Atomoxetine**		**Clomethiazole**		**Clozapine**		**Flecainide**	
Detection wavelength(s)	210 nm		254 nm		250 nm		ex 300 nm/ em 370 nm	
Solvent gradients	Time (min)	%B	Time (min)	%B	Time (min)	%B	Time (min)	%B
Eluent A: 0.1% formic acid (aq.) Eluent B: 0.1% formic acid in ACN	0<br>3.5<br>4<br>4.5	10<br>90<br>90<br>10	0<br>3.5<br>4<br>4.5	10<br>90<br>10<br>10	0<br>3.5<br>4<br>4.5	10<br>90<br>90<br>10	0<br>3.5<br>4<br>4.5	10<br>90<br>90<br>10
tR (min), CV (n)	2.38, 0.15% (14)		2.32, 0.08% (18)		1.98, 0.09% (14)		2.33, 0.12% (15)	
Vinj (µL)	10		10		10		10	
Linearity	y= 0.005238x - 0.054625		y = 0.001189x - 0.028261		y = 0.006996x - 0.077946		y = 0.085017x + 0.439936	
R^2	0.9993		0.9301		0.9998		0.9820	
Detection range (nM)	50-2000		100-2000		25-2000		10-2000	

**Test substance**	**Orphenadrine**		**Quetiapine**		**Zolmitriptan**	
Detection wavelength(s)	ex 220 nm/ em 285 nm		254 nm		ex 225 nm/ em 360 nm	
Solvent gradients	Time (min)	%B	Time (min)	%B	Time (min)	%B
Eluent A: 0.1% formic acid (aq.) Eluent B: 0.1% formic acid in ACN	0<br>3.5<br>4<br>4.5	10<br>90<br>90<br>10	0<br>3.5<br>4<br>4.5	10<br>90<br>90<br>10	0<br>3.5<br>4<br>4.5	10<br>90<br>95<br>10
tR (min), CV (n)	2.42, 0.13% (13)		2.12, 0.05% (14)		1.54, 0.24% (15)	
Vinj (µL)	10		10		10	
Linearity	y = 0.013548x - 0.422715		y = 0.004142x - 0.105741		y = 0.080060x - 1.755847	
R^2	0.9993		0.9884		0.9990	
Detection range (nM)	50-2000		25-2000		10-2000	

SI Table 5 (cont.)

LC-UV/vis/fluorescence analysis (Lab 3): Agilent 1200 with DAD (G1315C) and fluorescence (G1321A) detectors

Separation column: Agilent Poroshell 120 EC-C18 (4.6x50mm, 2.7 µm)

**Test substance**	**Bimatoprost**		**Cilostazol**		**Desloratadine**		**Fluphenazine**		**Imiquimod**	
Detection wavelength(s)	210 nm		254 nm		ex 280 nm/<br>em 452 nm		257 nm		242 nm	
Solvent gradients	Time (min)	%B	Time (min)	%B	Time (min)	%B	Time (min)	%B	Time (min)	%B
Eluent A: 0.1% phosphoric acid (aq.)<br>Eluent B: 0.1% phosphoric acid in ACN	0<br>1<br>5<br>5.5<br>6<br>6.5	10<br>30<br>80<br>90<br>90<br>10	0<br>1<br>4<br>5<br>5.5<br>6	10<br>30<br>80<br>90<br>90<br>10	0<br>1<br>4<br>4.05<br>4.5<br>4.6	10<br>10<br>25<br>90<br>90<br>10	0<br>1<br>4<br>5<br>5.5<br>6	10<br>60<br>80<br>90<br>90<br>10	0<br>1<br>4<br>4.5<br>5<br>5.5	10<br>30<br>80<br>90<br>90<br>10
tR (min),<br>CV (n)	2.67,<br>0.45% (45)		2.62,<br>0.17% (45)		2.8,<br>0.36% (60)		1.28,<br>0.35% (45)		1.51,<br>0.14% (45)	
Vinj (µL)	10		10		5		10		10	
Linearity	y=0.003202x<br>+0.105068		y=0.004482x<br>-0.142133		y=0.006538x -<br>0.062910		y=0.005546x<br>-0.036954		y=0.013871x<br>+0.081569	
R^2	0.996542		0.998673		0.999793		0.998554		0.994179	
Detection range (nM)	50-2000		50-2000		25-2000		10-2000		25-2000	
**Test substance**	**Indapamide**		**Indomethacin**		**Ketoconazole**		**Mephen-oxalone**		**Mycophenolic acid**	
Detection wavelength(s)	242 nm		202 nm		231 nm		ex 280 nm/<br>em 310 nm		215 nm	
Solvent gradients	Time (min)	%B	Time (min)	%B	Time (min)	%B	Time (min)	%B	Time (min)	%B
Eluent A: 0.1% phosphoric acid (aq.)<br>Eluent B: 0.1% phosphoric acid in ACN	0<br>1<br>4<br>5<br>5.5<br>6	10<br>30<br>80<br>90<br>90<br>10	0<br>1<br>3.5<br>3.55<br>4.05<br>4.1	60<br>60<br>52<br>10<br>60<br>60	0<br>1<br>4<br>5<br>5.5<br>6	10<br>30<br>80<br>90<br>10<br>10	0<br>1<br>5<br>5.5<br>6<br>6.5	10<br>30<br>80<br>90<br>90<br>10	0<br>1<br>5<br>5.05<br>5.5<br>5.6	25<br>25<br>35<br>90<br>90<br>25
tR (min),<br>CV (n)	2.34,<br>0.11% (45)		3.28,<br>0.22% (45)		1.95,<br>0.08% (45)		1.65,<br>0.13% (45)		4.2,<br>0.37% (60)	
Vinj (µL)	10		10		10		10		10	
Linearity	y=0.004582x<br>+0.746542		y=0.003077x<br>-0.188833		y=0.002834x<br>-0.033296		y=0.023328x<br>-0.790660		y=0.005464x<br>-0.010162	
R^2	0.985449		0.986650		0.998436		0.996861		0.998948	
Detection range (nM)	100-2000		50-2000		25-2000		25-2000		25-2000	

SI Table 5 (cont.)

LC-UV/vis/fluorescence analysis (Lab 3): Agilent 1200 with DAD (G1315C) and fluorescence (G1321A) detectors

Separation column: Agilent Poroshell 120 EC-C18 (4.6x50mm, 2.7 µm)

**Test substance**	**Nimesulide**		**Olaparib**		**Perazine**		**Prazosin**		**Procyclidine**	
Detection wavelength(s)	215 nm		206 nm		250 nm		ex 330nm/<br>em 390 nm		210 nm	
Solvent gradients	Time (min)	%B	Time (min)	%B	Time (min)	%B	Time (min)	%B	Time (min)	%B
Eluent A: 0.1% phosphoric acid (aq.)<br>Eluent B: 0.1% phosphoric acid in ACN	0<br>0.1<br>2<br>3<br>3.1<br>4	30<br>60<br>80<br>90<br>90<br>30	0<br>1<br>4<br>4.05<br>4.5<br>4.6	15<br>15<br>45<br>90<br>90<br>15	0<br>1<br>4<br>5<br>5.5	10<br>40<br>80<br>90<br>10	0<br>0.1<br>3<br>3.1<br>3.5	10<br>20<br>30<br>90<br>10	0<br>1<br>4<br>5<br>5.5	10<br>30<br>80<br>90<br>10
tR (min),<br>CV (n)	0.95,<br>0.60% (45)		3.4,<br>0.13% (45)		1.39,<br>0.78% (45)		1.81,<br>7.4% (45)		2.21,<br>1.14% (45)	
Vinj (µL)	30		5		10		1		20	
Linearity	y=0.005846x<br>+0.051376		y=0.004411x<br>+1.200038		Y=0.005561x-<br>0.100327		y=0.089332x<br>+0.022580		y=0.003557x<br>-0.127896	
R^2	0.984553		0.949426		0.999760		0.999782		0.999612	
Detection range (nM)	250-2000		250-2000		25-2000		25-2000		125-2000	

**Test substance**	**Propranolol**		**Tolbutamide**		**Tolperisone**		**Tribenoside**	
Detection wavelength(s)	ex 292 nm /<br>em 351 nm		200 nm		260 nm		220 nm	
Solvent gradients	Time (min)	%B	Time (min)	%B	Time (min)	%B	Time (min)	%B
Eluent A: 0.1% phosphoric acid (aq.)<br>Eluent B: 0.1% phosphoric acid in ACN	0<br>1<br>4<br>4.05<br>4.5<br>4.6<br>5.3	15<br>15<br>45<br>90<br>90<br>15<br>15	0<br>1<br>4<br>4.05<br>4.5<br>4.6	15<br>15<br>45<br>90<br>90<br>15	0<br>1<br>4<br>5<br>5.5<br>6	10<br>30<br>80<br>90<br>90<br>10	0<br>1<br>4<br>4.5<br>5<br>6	60<br>60<br>80<br>90<br>90<br>60
tR (min),<br>CV (n)	2.9,<br>0.18% (60)		4.27,<br>0.25% (45)		1.61,<br>0.09% (45)		3.47,<br>0.22% (45)	
Vinj (µL)	5		10		10		40	
Linearity	y=0.065741x<br>-0.196744		Y=0.003837x<br>+0.999120		Y=0.002033x<br>+ 0.416644		y=0.002942x<br>+1.1899456	
R^2	0.999955		0.966499		0.996390		0.971721	
Detection range (nM)	25-2000		250-2000		100-2000		100-2000	

SI Table 6: Currently available fish plasma protein binding data (and supporting metadata) for APIs

In vivo fish data for APIs 1-14 data were obtained from Phish-Pharm (US FDA, 2024). APIs 1-2 are antifungals; APIs 3-14 are antibiotics. Dosage: sd (single dose). *In vivo* dosing routes: Bath (immersion), PO (oral; includes gavage or in feed), IC (intra-cardiac), IV (intra-vascular), IVC (intra-vascular cannulated).

In vitro fish data for APIs 15-26: dosing route denoted SP (spiked plasma). Note f_u was measured in PBS buffer @ pH 7.4 (see Refs 19 and 20 below).

Human data (*in vitro*) were obtained from Refs 19, 21-23.

References numbered in table: 1 Plakas et al. (1991); 2 Plakas and James (1989); 3 Nouws et al. (1988); 4 Plakas et al. (2000); 5 Boon et al. (1991); 6 Plakas et al. (1994); 7 Jarobe et al. (1993); 8 Ueno and Tatsuno (2003); 9 Bjorklund and Bylund (1991); 10 Uno (1996); 11 Hayton (1997); 12 Alavi et al. (1993); 13 Kleinow et al. (1992); 14 Kleinow and Lech (1988); 15 Squibb et al. (1988); 16 Michel et al. (1990); 17 Uno et al. (1996); 18 Plakas et al. (1988); 19 Henneberger et al. (2020); 20 Nolte et al. (2018); 21 Krumpholz et al. (2024); 22 Berninger et al. (2016); 23 DrugBank (2025).

API	Fish common name	Fish latin name	Mean fish weight (g)	Assay Temp (°C)	Dose (mg/kg)	Dose & Route	Fish f_u mean & range	Human f_u mean (Ref)	Rf_u	Ref
***In vivo* data**										
1 Aflatoxin B1	Channel catfish	*Ictalurus punctatus*	400	28	0.25	sd / PO	0.05 ±0.02	-	-	1
2 Benzoic acid (acid)	Channel catfish	*Ictalurus punctatus*	500	28	10	sd / IVC	0.82 ±0.01	-	-	2
2 Benzoic acid (acid)	Catfish, channel	*Ictalurus punctatus*	500	28	10	sd / PO	0.83 ±0.02	-	-	2
3 Ciprofloxacin (base)	Common carp	*Cyprinus carpio*	-	20	15	sd / IV	0.784 ±0.02	0.484 [21]	1.62	3
3 Ciprofloxacin (base)	African Catfish	*Clarias gariepinus*	-	25	15	sd / IV	0.798 ±0.03	0.484 [21]	1.65	3
3 Ciprofloxacin (base)	Rainbow trout	*Oncorhynchus mykiss*	-	12	15	sd / IV	0.769 ±0.02	0.484 [21]	1.59	3
4 Flumequine (acid)	Channel catfish	*Ictalurus punctatus*	660	24	1	sd / IVC	0.285 ±0.17	-	-	4
4 Flumequine (acid)	European eel	*Anguilla anguilla*	488	23	9	sd / IM	0.65 ±0.05	-	-	5
5 Furazolidone (acid)	Channel catfish	*Ictalurus punctatus*	680	24	1	sd / IV	0.6	-	-	6
6 Furazolidone (acid)	Channel catfish	*Ictalurus punctatus*	680	24	1	sd / PO	0.46 ±0.07	-	-	6
7 Nalidixic acid (acid)	Rainbow trout	*Oncorhynchus mykiss*	987	14	5	sd / IV	0.84 ±0.12	0.07 [23]	12	7
8 Oxolinic acid (acid)	Rainbow trout	*Oncorhynchus mykiss*	126	15	20	sd / IV	0.55 ±0.24	-	-	8
8 Oxolinic acid (acid)	Rainbow trout	*Oncorhynchus mykiss*	558	16	10	sd / IV	0.73	-	-	9
9 Oxytetracycline (base)	Ayu	*Plecoglossus altivelis*	54	18	100	sd / PO	0.32 ±0.03	-	-	10
9 Oxytetracycline (base)	Ayu	*Plecoglossus altivelis*	54	18	100	sd / PO	0.3 ±0.04	-	-	10
9 Oxytetracycline (base)	Rainbow trout	*Oncorhynchus mykiss*	558	16	20	sd / IV	0.45	-	-	9
10 Proflavine (base)	Rainbow trout	*Oncorhynchus mykiss*	601	12	4 mg/L	4h /BATH	0.191	-	-	11
11 (acid) Sulfachlorpyridazine	Channel catfish	*Ictalurus punctatus*	312	22	60	sd / IC	0.906 ±0.05	-	-	12

API	Fish common Name	Fish latin name	Mean fish weight (g)	Assay Temp (°C)	Dose (µM)	Dose & Route	Fish f_u mean & range	Human f_u mean	Rf_u	Ref
12 Sulfadimethoxine (acid)	Rainbow trout	*Oncorhynchus mykiss*	637	13	42	sd / IVC	0.842 ±0.05	-	-	13
12 Sulfadimethoxine (acid)	Rainbow trout	*Oncorhynchus mykiss*	415	-	42	sd / IVC	0.83	-	-	14
12 Sulfadimethoxine (acid)	Channel catfish	*Ictalurus punctatus*	35	-	40	sd / IV	0.816 ±0.03	-	-	15
12 Sulfadimethoxine (acid)	Channel catfish	*Ictalurus punctatus*	500	-	40	sd / IV	0.816 ±0.03	-	-	15
12 Sulfadimethoxine (acid)	Channel catfish	*Ictalurus punctatus*	500	20	40	sd / IV	0.818	-	-	16
13 Sulfa-1-methoxine (acid)	Amber jack	*Seriola quinqueradiata*	638	21	100	sd / IV	0.942 ±0.02	-	-	17
13 Sulfa-1-methoxine (acid)	Rainbow trout	*Oncorhynchus mykiss*	171	15	100	sd / IV	0.936 ±0.02	-	-	17
14 Tetracycline (base)	Channel catfish	*Ictalurus punctatus*	760	27	4	sd / IV	0.28 ±0.04	-	-	18
***In vitro* data**										
15 Diclofenac (acid)	Rainbow trout	*Oncorhynchus mykiss*	200	20	?	24h /SP	0.01	0.002 [21]	5.0	19
16 Ibuprofen (acid)	Rainbow trout	*Oncorhynchus mykiss*	200	20	?	24h /SP	0.06	0.004 [21]	15.0	19
16 Ibuprofen (acid)	Rainbow trout	*Oncorhynchus mykiss*	2000	15	5	~8h /SP	0.11	0.004 [21]	27.5	20
16 Ibuprofen (acid)	Zebrafish	*Danio rerio*	0.3-1.0	28	5	~8h /SP	0.099	0.004 [21]	24.8	20
17 Naproxen (acid)	Rainbow trout	*Oncorhynchus mykiss*	200	20	?	24h /SP	0.054	6.6E-4 [19]	82.0	19
18 Warfarin (acid)	Rainbow trout	*Oncorhynchus mykiss*	200	20	?	24h /SP	0.121	0.007 [19]	17.3	19
19 Genistein (acid)	Rainbow trout	*Oncorhynchus mykiss*	200	20	?	24h /SP	0.03	0.008 [19]	3.8	19
20 Diphenhydramine (acid)	Rainbow trout	*Oncorhynchus mykiss*	200	20	?	24h /SP	0.15	0.103 [19]	1.5	19
21 Torasemide (acid)	Rainbow trout	*Oncorhynchus mykiss*	200	20	?	24h /SP	0.083	0.004[19]	20.8	19
22 Metoprolol (base)	Rainbow trout	*Oncorhynchus mykiss*	200	20	?	24h /SP	0.156	0.511 [19]	0.3	19
23 Propranolol (base)	Rainbow trout	*Oncorhynchus mykiss*	200	20	?	24h /SP	0.107	0.37 [19]	0.29	19
23 Propranolol (base)	Rainbow trout	*Oncorhynchus mykiss*	2000	15	5	~8h /SP	0.144	0.37 [19]	0.39	20
23 Propranolol (base)	Zebrafish	*Danio rerio*	0.3-1.0	28	5	~8h /SP	0.102	0.37 [19]	0.28	20
23 Propranolol (base)	Fathead minnow	*Pimephales promelas*	5-10	25	5	~8h /SP	0.136	0.37 [19]	0.37	20
23 Propranolol (base)	Common carp	*Cyprinus carpio*	150	15	5	~8h /SP	0.145	0.37 [19]	0.39	20
23 Propranolol (base)	Common carp	*Cyprinus carpio*	150	28	5	~8h /SP	0.194	0.37 [19]	0.52	20
24 Venlafaxine (base)	Rainbow trout	*Oncorhynchus mykiss*	200	20	?	24h /SP	0.09	0.179 [19]	0.5	19
25 Lamotrigine (base)	Rainbow trout	*Oncorhynchus mykiss*	200	20	?	24h /SP	0.261	0.216 [19]	1.2	19
26 Caffeine (base)	Rainbow trout	*Oncorhynchus mykiss*	200	20	?	24h /SP	0.639	0.365 [19]	1.8	19

SI Table 7: Total protein concentration in fish blood serum (and plasma)

The majority of data quantifying mean total protein concentrations in fish relate to serum (which lacks clotting proteins); data relating to plasma are highlighted in parentheses. References numbered: 1) De Smet, 1978 (Micro-biuret method used on serum); 2) Fellows et al., 1980 (Biuret method used on serum); 3) Henneberger et al., 2022 (Lowry method used on plasma); 4) Sandnes et al., 1988 (Biuret method used on serum). 5) Hunn and Greer, 1990 (Lowry method used on serum, Bradford method used on plasma).

**Order/ Family**	**Species common name**	**Species Latin name**	**Total protein concentration (mg/mL) or (mg/g)***	**Reference**
Salmoniformes/Salmonidae	Brook trout	*Salvelinus fontinalis*	50	1
	Brown trout	*Salmo trutta*	36.8	1
	European smelt	*Osmerus eperlanus*	43.5	1
	Rainbow trout	*Oncorhynchus mykiss*	31*	2
			39.7 (plasma)	3
	Atlantic salmon	*Salmo salar*	42.3	4
Cypriniformes/Cyprinidae	Common bream	*Abramis brama*	25.7	1
	Common bleak	*Alburnus alburnus*	25	1
	Goldfish	*Carassius auratus*	31.2	1
	Common carp	*Cyprinus carpio*	27.7	1
	Common dace	*Leuciscus leuciscus*	33.3	1
	Roach	*Rutilus rutilus*	30.7	1
Cypriniformes/Tincidae	Tench	*Tinca tinca*	32.5	1
Siluriformes/Ictaluridae	Brown bullhead	*Ameiurus nebulosus*	37.5	1
Gadiformes/Gaidropsaridae	Five-bearded rockling	*Onos mustelus*	42.5	1
Perciformes/Percidae	Eurasian perch	*Perca fluviatilis*	27.7	1
Perciformes/Psychrolutidae	Shorthorn sculpin	*Myoxocephalus scorpius*	31	1
Acanthuriformes/ Moronidae	Striped bass	*Morone saxatilis*	38<br>36 (plasma)	5
Pleuronectiformes/ Pleuronectidae	European flounder	*Platichthys flesus*	17.1	1
	European plaice	*Pleuronectes platessa*	38	1
Ceratodontiformes/ Protopteridae	Lungfish	*Protopterus* sp.	39.5	1
Scorpaeniformes/ Platycephalidae	Sand flathead	*Platycephalus bassensis*	61*	2
	Long-nosed flathead	*Platycephalus caeruleopunctatus*	56*	2
Tetraodontiformes	Globefish	Species unknown	44	2
Myliobatiformes/ Urolophidae	White-spotted stingaree	*Urolophus paucimaculatus*	37*	2
	Common stingaree	*Trygonoptera testacea*	26*	2
Myliobatiformes/ Myliobatidae	Common eagle ray	*Myliobatis aquila*	39*	2
Rhinopristiformes/ Trygonorrhinidae	Southern fiddler ray	*Trygonorrhina dumerilii*	44*	2
Rajiformes/Rajidae	Melbourne skate	*Spiniraja whitleyi*	21*	2
Heterodontiformes/ Heterodontidae	Port Jackson shark	*Heterodontus portusjacksoni*	28*	2
Carcharhiniformes/ Scyliorhinidae	Draughtsboard shark	*Cephaloscyllium isabellum*	24*	2
Petromyzontiformes/ Mordaciidae	Short headed lamprey	*Mordacia mordax*	62*	2

SI Table 8: Assessment of non-specific binding of APIs – measurement of percentage recovery

Lower recoveries (<50%) are highlighted in bold

API name	RECOVERY mean (%) HUMAN		RECOVERY mean (%) CARP	RECOVERY mean (%) TROUT			RECOVERY mean (%) MINNOW	
	Lab 1	Lab 2	Lab 1	Lab 1	Lab 2	Lab3	Lab 1	Lab 2
**Aripiprazole**	84.9		100.9	106.4			98.1	
**Ataluren**	75.4		84.2	131.5		89.45	91.1	
**Atomoxetine**								
**Atorvastatin**			87.0	114.2		107.3	94.6	
**Azelastine**	98.6		95.9			70.7	83.4	
**Bimatoprost**	89.2		85.3			**33.5**	94.5	
**Cilostazol**	95		73.5			**15.6**	80.1	
**Clomethiazole**	74.3		90.4	100.5			86.9	
**Clomipramine**	90.9		80.6	106.3			92	
**Clozapine**	86.2		92.8	77.3			86	
**Desloratadine**	101.7		152.6			69.97	88.6	
**Diclofenac**			95	148.8		-	99	
**Felbinac**						122.2		
**Fexofenadine**	92.5		82.6	122.3			87.6	
**Flecainide**	80.7		79.9	97.9		-	86.9	
**Fluphenazine**	88.0		81.8			93.9	88.8	
**Haloperidol**	83.5		88.4	95.8		-	90.3	
**Ibuprofen**			-	-		-	-	
**Imiquimod**	92.1		90.1			**25.8**	91.1	
**Indapamide**	89.4		83.2			49.4	96.2	
**Indomethacin**			87.1	106.1		118.8	109.2	
**Ketoconazole**						56.2		
**Ketoprofen**	83.9		79.6	129			90.2	

**Levomepromazine**	85.4		83.8			111.77	124.8	
**Meloxicam**	81.9		85.3	135.1			95.7	
**Mephenoxalone**						67.5		
**Mycophenolic acid**	91.3	91	90.1		118	**47.2**	96.8	111
**Nimesulide**	89.3		85.6			85.3	91.7	
**Olaparib**	88.2	86	99.6	93.7	98	89.4	100.8	97
**Orphenadrine**	84.8		98.1	98.9			95.6	
**Perazine**						85.0		
**Phenylbutazone**	89.7		74.7	108.7			70.8	
**Prazosin**						**46.6**		
**Procyclidine**						60.0		
**Propranolol**	82.3		82.3			64.6	80	
**Quetiapine**	91.1	100	79.2		99		85.9	94
**Salinomycin**			72.4	85.7			90.8	
**Sulpiride**						**28.52**		
**Ticlopidine**	110		94				99	
**Tolbutamide**	77.3		68.1			83.1	117.1	
**Tolperisone**	68.8		87.5	92.6		75	82	
**Tribenoside**						44.3		
**Verapamil**	90.5		101.4	122			93.2	
**Zolmitriptan**								

SI Table 9a: Variation between analytical laboratories in measured unbound fractions of APIs selected for intercomparison in fish and humans

Variation between maximum and minimum f_u values is shown in this table

Fish species: Carp = koi carp; FHM = fathead minnow; Trout = rainbow trout

API name	Human f_u min	Human f_u max	Human fu max/ Human f_u min	Trout f_u min	Trout f_u max	Trout f_u max/ Trout f_u min	FHM f_u min	FHM f_u max	FHM f_u max/ FHM f_u min	Carp f_u min	Carp f_u max	Carp f_u max/ Carp f_u min
Aripiprazole	0.001	-	-	-	0.0004	-	0.0002	0.0003	1.5	0.0002	0.0003	1.5
Clomethiazole	0.35	0.401	1.1	0.282	0.432	1.5	0.2535	0.289	1.1	0.468	0.4835	1
Clomipramine	0.029	0.118	4.1	-	0.0114	-	0.0014	0.0021	1.5	0.0041	0.0043	1
Clozapine	0.046	-	-	0.015	0.027	1.8	0.0192	0.0221	1.2	0.0258	0.0409	1.6
Flecainide	0.586	0.843	1.4	0.153	0.397	2.6	0.066	0.0787	1.2	0.1184	0.1317	1.1
Mycophenolic acid	0.02	0.032	1.6	0.341	0.564	1.7	0.108	0.2382	2.2	-	0.2409	-
Olaparib	0.17	0.18	1.1	0.357	0.576	1.6	0.567	0.9553	1.7	-	0.4585	-
Orphenadrine	0.05	0.167	3.3	0.093	0.189	2	0.0131	0.0172	1.3	0.0182	0.0209	1.1
Propranolol	0.1	0.282	2.8	0.15	0.256	1.7	0.0747	0.083	1.1	-	0.1246	-
Quetiapine	0.092	0.17	1.8	0.049	0.186	3.8	0.0409	0.093	2.3	-	0.074	-
Tolperisone	0.05	0.178	3.6	0.463	0.525	1.1	0.0078	0.0121	1.6	0.0113	0.0116	1

SI Table 9b: Variation in measurements of f_u within and between individual analytical laboratories

Variations in mean f_u measurements and their standard deviations are shown for each analytical lab. Results in bold show variations between labs.

Fish species: Carp = koi carp; FHM = fathead minnow; Trout = rainbow trout

API name	FU mean ± SD (n) HUMAN		FU mean ± SD (n) CARP	FU mean ± SD (n) TROUT			FU mean ± SD (n) MINNOW	
	Lab 1	Lab 2	Lab 1	Lab 1	Lab 2	Lab3	Lab 1	Lab 2
**Aripiprazole**	0.001 ± 0.0001 (n=4)		0.0003 ± 0.0001 (n=4)	0.0004 ± 0.0 (n=2)		-	0.0002 ± 0.0001 (n=4)	
**Ataluren**	0.004 ± 0.0004 (n=2)		0.019 ± 0.0004 (n=2)	**0.034 ± 0.0111 (n=2)**		**0.014 ± 0.0025 (n=3)**	0.024 ± 0.0013 (n=2)	
**Atomoxetine**						0.032 ± 0.006 (n=3)		
**Atorvastatin**			0.021 ± 0.0006 (n=2)	**0.03 ± 0.0028 (n=2)**		**0.008 ± 0.0016 (n=3)**	0.0175 ± 0.0013 (n=2)	
**Azelastine**	0.077 ± 0.0003 (n=2)		0.015 ± 0.0001 (n=2)			0.013 ± 0.005 (n=3)	0.0164 ± 0.0022 (n=2)	
**Bimatoprost**	0.14 ± 0.0182 (n=2)		0.166 ± 0.054 (n=2)			0.23 ± 0.03 (n=3)	0.129 ± 0.0359 (n=2)	
**Cilostazol**	0.048 ± 0.004 (n=2)		0.134 ± 0.0182 (n=2)			0.27 ± 0.06 (n=3)	0.097 ± 0.0284 (n=2)	
**Clomethiazole**	0.41 ± 0.0136 (n=4)		0.476 ± 0.034 (n=4)	**0.456 ± 0.0278 (n=4)**		**0.28 ± 0.06 (n=3)**	0.271 ± 0.0359 (n=4)	
**Clomipramine**	0.035 ± 0.0015 (n=8)		0.0042 ± 0.0005 (n=4)	0.0114 ± 0.0013 (n=2)			0.0017 ± 0.0004 (n=4)	
**Clozapine**	0.047 ± 0.0025 (n=4)		0.033 ± 0.0098 (n=4)	**0.0265 ± 0.0024 (n=2)**		**0.015 ± 0.002 (n=3)**	0.021 ± 0.0025 (n=4)	
**Desloratadine**	0.126 ± 0.012 (n=2)		0.077 ± 0.0038 (n=2)			0.03 ± 0.01 (n=3)	0.046 ± 0.0033 (n=2)	
**Diclofenac**			0.0054 ± 0.0006 (n=2)	0.0077 ± 0.0008 (n=2)			0.007 ± 0.0004 (n=2)	
**Felbinac**						0.03 ± 0.01 (n=3)		
**Fexofenadine**	0.22 ± 0.0155 (n=2)		0.12 ± 0.011 (n=2)	0.103 ± 0.0052 (n=2)			0.128 ± 0.0313 (n=2)	
**Flecainide**	0.6 ± 0.021 (n=4)		0.054 ± 0.003 (n=2)	**0.397 ± 0.025 (n=2)**		**0.15 ± 0.07 (n=3)**	0.045 ± 0.0048 (n=2)	
**Fluphenazine**	0.0144 ± 0.0004 (n=2)		0.004 ± 0.0003 (n=2)			0.0 ± 0 (n=3)	0.0016 ± 0.0001 (n=2)	
**Haloperidol**	0.127 ± 0.005 (n=4)		0.02 ± 0.002 (n=4)	0.067 ± 0.0046 (n=2)			0.015 ± 0.0024 (n=4)	

**Ibuprofen**					0.164 ± 0.009 (n=4)			
**Imiquimod**	0.15 ± 0.0006 (n=2)		0.17 ± 0.0022 (n=2)			0.12 ± 0.002 (n=3)	0.062 ± 0.0014 (n=2)	
**Indapamide**	0.16 ± 0.016 (n=2)		0.346 ± 0.042 (n=2)			0.21 ± 0.06 (n=3)	0.169 ± 0.0352 (n=2)	
**Indomethacin**			0.005 ± 0.0003 (n=2)	**0.014 ± 0.0023 (n=2)**		**0.08 ± 0.006 (n=3)**	0.0053 ± 0.0001 (n=2)	
**Ketoconazole**						0.11 ± 0.02 (n=3)		
**Ketoprofen**	0.006 ± 0.012 (n=2)		0.122 ± 0.013 (n=2)	0.12 ± 0.0153 (n=2)			0.159 ± 0.0123 (n=2)	
**Levomepromazine**	0.036 ± 0.005 (n=2)		0.0094 ± 0.0006 (n=2)			0.004 ± 0.005 (n=3)	0.0058 ± 0.0001 (n=2)	
**Meloxicam**	0.0035 ± 0.0001 (n=2)		0.104 ± 0.013 (n=2)	0.1 ± 0.0081 (n=2)			0.113 ± 0.039 (n=2)	
**Mephenoxalone**						0.8 ± 0.07 (n=3)		
**Mycophenolic acid**	**0.009 ± 0.001 (n=2)**	**0.016 ± 0.001 (n=6)**	0.24 ± 0.068 (n=2)		**0.564 ± 0.005 (n=6)**	**0.34 ± 0.03 (n=3)**	**0.238 ± 0.0886 (n=2)**	**0.108 ± 0.008 (n=6)**
**Nimesulide**	0.005 ± 0.0004 (n=2)		0.053 ± 0.0066 (n=2)			0.13 ± 0.02 (n=3)	0.0414 ± 0.0026 (n=2)	
**Olaparib**	**0.095 ± 0.003 (n=4)**	**0.17 ± 0.006 (n=6)**	0.412 ± 0.068 (n=4)	**0.428 ± 0.0001 (n=2)**	**0.576 ± 0.017 (n=6)**	**0.36 ± 0.07 (n=3)**	**0.666 ± 0.564 (n=4)**	**0.116 ± 0.003 (n=6)**
**Orphenadrine**	0.162 ± 0.007 (n=2)		0.035 ± 0.0013 (n=2)	**0.189 ± 0.0077 (n=2)**		**0.093 ± 0.014 (n=3)**	0.039 ± 0.0013 (n=2)	
**Perazine**						0.044 ± 0.008 (n=3)		
**Phenylbutazone**	0.0017 ± 0.0001 (n=2)		0.018 ± 0.0032 (n=2)	0.17 ± 0.0128 (n=2)			0.195 ± 0.0168 (n=2)	
**Prazosin**						0.12 ± 0.001 (n=3)		
**Procyclidine**						0.42 ± 0.005 (n=3)		
**Propranolol**			0.125 ± 0.0021 (n=2)		**0.256 ± 0.03 (n=4)**	**0.15 ± 0.044 (n=3)**	**0.075 ± 0.0048 (n=2)**	**0.083 ± 0.002 (n=8)**
**Quetiapine**	**0.10 ± 0.0078 (n=2)**	**0.092 ± 0.004 (n=6)**	0.074 ± 0.0033 (n=2)		0.186 ± 0.012 (n=6)		**0.041 ± 0.0002 (n=2)**	**0.093 ± 0.005 (n=6)**
**Salinomycin**			0.008 ± 0.0029 (n=2)	0.028 ± 0.0027 (n=2)			0.0053 ± 0.0005 (n=2)	
**Sulpiride**						0.50 ± 0.144 (n=3)		
**Ticlopidine**	0.007 ± 0.0003 (n=2)		0.003 ± 0.0002 (n=2)		0.002 ± 0.0 (n=4)		**0.0015 ± 0.0 (n=2)**	**0.039 ± 0.007 (n=6)**
**Tolbutamide**	0.015 ± 0.0003 (n=2)		0.549 ± 0.0534 (n=2)			0.56 ± 0.007 (n=3)	0.28 ± 0.1061 (n=2)	

**Tolperisone**	0.174 ± 0.0084 (n=4)		0.125 ± 0.009 (n=4)	**0.525 ± 0.0066 (n=2)**		**0.46 ± 0.08 (n=3)**	0.0724 ± 0.0077 (n=4)	
**Tribenoside**						0.01 ± 0.00 (n=3)		
**Verapamil**	0.115 ± 0.0094 (n=8)		0.0114 ± 0.0007 (n=4)	0.112 ± 0.0115 (n=4)			0.0099 ± 0.0006 (n=4)	
**Zolmitriptan**						0.69 ± 0.08 (n=3)		

SI Table 10: Therapeutic water concentrations predicted for rainbow trout, koi carp, fathead minnow versus observed effect concentrations for APIs

Trout = rainbow trout (blood pH 7.9), carp = koi carp (blood pH 7.7), FHM = fathead minnow (blood pH 7.7).

Therapeutic Water Concentrations (TWC) were calculated according to the refined FPM (Eq 2.1) using both median and maximum Rf_u:
TWC for each fish species = $(C_{max} / Rf_u) / P_{blood:water}$
Where: $P_{blood:water} = 10^{(0.73 \times \text{Log Dow} - 0.88)}$

Log D_{ow} (pH) values were obtained from Chemaxon, Marvin 23.12. Human C_{max} values and LOECs for fish are referenced in **Table 4** in the main manuscript.

Speciation (charge @ pH7.4 to 7.9)	API name (Log Dow, charge)	C_{max} (mg/L)	Log D_{ow} (pH 7.9)	Trout Pb:w	Trout Rf_u median	Trout TWC - Rf_u median (mg/L)	Trout Rf_u max	Trout TWC - Rf_u max (mg/L)	Log D_{ow} (pH 7.7)	Carp & FHM Pb:w	Carp Rf_u median	Carp TWC - Rf_u median (mg/L)	Carp Rf_u max	Carp TWC - Rf_u max (mg/L)	FHM Rf_u median	FHM TWC - Rf_u median (mg/L)	FHM Rf_u max	FHM TWC - Rf_u max (mg/L)	LOEC for fish (mg/L)
A (-)	Meloxicam (-1.12)	0.4	-1.75	0.01	15	3.85	29	2.01	-1.49	0.01	16	2.39	30	1.25	17	2.20	32	1.15	
A (-)	Mycophenolic acid (-0.43)	1	-0.58	0.05	19	1.07	56	0.36	-0.54	0.05	10	1.87	24	0.78	7	2.61	24	0.79	0.3
A (-)	Ketoprofen (0.24)	4.22	-0.02	0.13	4	8.83	30	1.10	0.07	0.15	4	7.47	30	0.93	5	5.72	40	0.72	
A (-)	Indomethacin (0.38)	0.025	0.02	0.14	2	0.11	28	0.01	0.15	0.17	0.1	1.55	2	0.09	0.1	1.43	1.8	0.08	
A (-)	Felbinac (0.73)	0.86	0.31	0.22	0.6	6.05	0.6	6.05	0.47	0.29	-	-	-	-	-	-	-	-	
A (-)	Diclofenac (0.75)	0.5	0.49	0.30	1.4	1.23	6	0.30	0.58	0.35	0.9	1.51	4	0.37	1.2	1.15	5	0.28	1.0
A (-)	Mephenoxalone (1.1)	2.85	1.1	0.84	2	1.92	2	1.71	1.1	0.84	-	-	-	-	-	-	-	-	
A (-)	Nimesulide (1.13)	0.1	0.94	0.64	7	0.02	13	0.01	1	0.71	3	0.05	5	0.03	2	0.06	4	0.03	
A (-)	Ataluren (1.15)	2	0.92	0.62	4	0.92	4	0.92	0.99	0.70	5	0.61	5	0.61	6	0.48	6	0.48	
A (-)	Tolbutamide (1.27)	45	1.21	1.01	17	2.62	35	1.27	1.23	1.04	17	2.60	34	1.26	8	5.09	18	2.47	
A (-)	Ibuprofen (1.31)	15	0.87	0.57	12	2.12	55	0.48	1.04	0.76	-	-	-	-	-	-	-	-	
A (-)	Atorvastatin (2.01)	0.0164	1.66	2.15	0.2	0.03	0.4	0.02	1.79	2.67	0.6	0.01	1.0	0.01	0.5	0.01	0.9	0.01	
A (-)	Phenylbutazone (2.26)	12.5	2.14	4.81	16	0.17	100	0.03	2.18	5.15	2	1.51	10	0.24	18	0.14	115	0.02	
A (-)	Salinomycin (4.14)	-	3.77	74.49	0.4	-	0.4	-	3.9	92.68	0.1	-	0.1	-	0	-	0.1	-	
a (0)	Olaparib (1.75)	7.3	1.75	2.50	3	0.94	6	0.51	1.75	2.50	3	0.96	5	0.64	5	0.58	10	0.31	1.0
a (0)	Indapamide (2.51)	0.0558	2.5	8.81	1	0.01	1	4.7E-03	2.5	8.81	2	3.8E-03	2	2.9E-03	0.8	0.01	1.1	0.01	
a (0)	Tribenoside (4.73)	-	4.73	374.02	0	-	0	-	4.73	374.02	-	-	-	-	-	-	-	-	

N (0)	Bimatoprost (2.35)	8E-05	2.35	6.85	2	5.7E-06	2	5.7E-06	2.35	6.85	2	7.8E-06	2	7.8E-06	1.2	1.0E-05	1.2	1.0E-05	
N (0)	Cilostazol (2.97)	1.2	2.97	19.41	8	0.01	13	4.6E-03	2.97	19.41	4	0.02	7	0.01	2.8	0.02	5	0.01	
c (0)	Clomethiazole (1.58)	0.1	1.58	1.88	1.0	0.06	1.2	0.04	1.58	1.88	1.3	0.04	1.4	0.04	0.7	0.07	0.8	0.06	
c (0)	Imiquimod (2.71)	0.0003	2.71	12.54	1	2.1E-05	2	1.0E-05	2.71	12.54	2	1.5E-05	3	7.4E-06	0.6	4.1E-05	1.2	2.1E-05	
c (0)	Quetiapine (3.05)	0.1	3.16	26.72	0.9	4.3E-03	2	2.0E-03	3.13	25.40	0.5	0.01	0.7	0.01	0	0.01	0.9	4.2E-03	0.32
c (0)	Ketoconazole (4.24)	4.22	4.26	169.75	1	0.02	12	2.1E-03	4.26	169.75	-	-	-	-	-	-	-	-	0.025
C (+)	Sulpiride (-1.56)	0.05	-1.09	0.02	0.7	3.49	0.8	2.84	-1.28	0.02	-	-	-	-	-	-	-	-	
C (+)	Zolmitriptan (-0.16)	0.007	0.32	0.23	0.9	0.03	0.9	0.03	0.13	0.16	-	-	-	-	-	-	-	-	3.2
C (+)	Flecainide (0.6)	0.4	1.08	0.81	0.4	1.28	0.7	0.73	0.89	0.59	0.1	9.01	0.1	7.39	0.1	10.70	0.1	8.77	
C (+)	Propranolol (0.63)	0.02	1.11	0.85	0.9	0.03	3	0.01	0.92	0.62	0.5	0.06	1.2	0.03	0.3	0.10	0.8	0.04	
C (+)	Prazosin (0.75)	0.001	1.09	0.82	2	6.9E-04	4	3.0E-04	0.96	0.66	-	-	-	-	-	-	-	-	
C (+)	Procyclidine (1.1)	0.08	1.59	1.91	2	0.02	3	0.02	1.39	1.36	-	-	-	-	-	-	-	-	
C (+)	Desloratadine (1.17)	0.004	1.63	2.04	0.2	0.01	0.2	0.01	1.44	1.48	0.5	0.01	0.6	4.6E-03	0.3	0.01	0.4	0.01	
C (+)	Atomoxetine (1.78)	0.064	2.26	5.89	0.2	0.05	0.2	0.05	2.07	4.28	-	-	-	-	-	-	-	-	
C (+)	Tolperisone (1.8)	0.16	2.26	5.89	4	0.01	11	2.6E-03	2.08	4.35	1.1	0.03	3	0.01	0.6	0.06	1.6	0.02	
C (+)	Clozapine (1.95)	0.3	2.41	7.57	0.2	0.21	0.9	0.04	2.23	5.60	0.3	0.17	1.4	0.04	0.2	0.28	0.7	0.07	0.031
C (+)	Haloperidol (2.27)	0.005	2.64	11.15	0.5	8.4E-04	2	2.0E-04	2.51	8.96	0.2	3.6E-03	0.7	8.0E-04	0.1	4.7E-03	0.6	9.7E-04	
C (+)	Levomepromazine (2.27)	0.046	2.75	13.41	0.1	0.07	0.1	0.05	2.56	9.75	0.1	0.04	0.2	0.03	0.1	0.06	0.1	0.05	
C (+)	Verapamil (2.36)	0.02	2.84	15.60	0.6	2.3E-03	2	7.5E-04	2.65	11.34	0.1	0.03	0.2	0.01	0.1	0.03	0.2	0.01	0.6
C (+)	Perazine (2.54)	0.008	2.99	20.08	0.9	4.3E-04	1	2.8E-04	2.82	15.09	-	-	-	-	-	-	-	-	
C (+)	Orphenadrine (2.6)	0.1	3.07	22.97	1.3	3.3E-03	4	1.2E-03	2.89	16.97	0.3	0.02	0.7	0.01	0.4	0.02	0.8	0.01	
C (+)	Azelastine (2.66)	0.002	3.11	24.56	0.1	9.4E-04	0.1	9.1E-04	2.94	18.46	0.1	1.1E-03	0.1	1.0E-03	0.1	9.9E-04	0.1	9.6E-04	
C (+)	Clomipramine (2.72)	0.09	3.21	29.06	0.2	0.02	0.4	0.01	3.02	21.12	0.1	0.07	0.1	0.03	0.0	0.18	0.1	0.06	
C (+)	Fluphenazine (2.74)	0.027	3.19	28.10	0.004	0.23	0.01	0.12	3.01	20.76	0.2	0.01	0.3	4.3E-03	0.1	0.02	0.1	0.01	
C (+)	Aripiprazole (3.14)	0.0273	3.62	57.89	0.1	0.01	0.3	1.4E-03	3.43	42.06	0.0	0.01	0.3	2.6E-03	0.04	0.01	0.3	2.6E-03	
C (+)	Ticlopidine (3.52)	0.31	3.8	78.34	0.2	0.02	2	2.0E-03	3.7	66.22	0.3	0.02	3	1.6E-03	2	2.4E-03	39	1.2E-04	

Z (+/-)	Fexofenadine (2.48)	0.131	2.47	8.38	0.3	0.05	0.5	0.03	2.47	8.38	0.4	0.04	0.5	0.03	0.4	0.04	0.6	0.03	

SI Table 11: Evidence of albumin-like proteins in fish blood plasma

* With the exception of rainbow trout, the number of proteins found in the salmonids is uncertain, the numbers provided have been deduced from Davidson et al. (1988).

Superclass: Osteichthyes (bony fishes); Class: Actinopterygii (ray-finned fishes)

Superorder/order/family	Species	Evidence	Reference
Protacanthopterygii Salmoniformes Salmonidae	Rainbow trout (*Oncoryhynchus mykiss*)	Proteins (n=2) with MW ~66 kDa, binds palmitate	Davidson et al. 1988; Davidson et al. 1989; Maillou and Nimmo 1993a
	Brown trout (*Salmo trutta*)	Proteins (n=1*) with MW ~66 kDa, binds palmitate	Davidson et al. 1988
	Brook trout (*Salvelinus fontinalis*)	Proteins (n=1*) with MW ~66 kDa, binds palmitate	Davidson et al. 1988
	Arctic charr (*Salvelinus alpinus)*	Not specified	Padrós and Pelletier, 2000
	Atlantic salmon (*Salmo salar*)	Proteins (n=1*) with MW ~66 kDa, binds palmitate	Davidson et al. 1988
	Pink salmon (*Oncorhynchus gorbuscha*	Proteins (n=2*) with MW ~66 kDa binds palmitate	Davidson et al. 1988
	Chinook salmon (*Oncorhynchus tshawytscha*)	Proteins (n=2*) with MW ~66 kDa binds palmitate	Davidson et al. 1988
	Coho salmon (*Oncorhynchus kisutch*	Proteins (n=2*) with MW ~66 kDa, binds palmitate	Davidson et al. 1988
Protacanthopterygii Esociformes Esocidae	Northern pike (*Esox lucius*)	Protein with similar mobility to albumin, binds to BCP	Mulcahy, 1969
Dipneusti Ceratodontiformes Neoceratodontidae	Australian lungfish, (*Neoceratodus forsteri*)	One protein with MW ~67 kDa	Metcalf et al., 2007

Superclass: Cyclostomi (jawless fishes); Class: Petromyzontida (lampreys)

Petromyzonti Petromyzontiformes Petromyzontidae	Sea lamprey (*Petromyzon marinus*)	One protein with MW ~175 kDa	Gray and Doolittle, 1992; Danis et al., 2000
	American brook lamprey (*Lethenteron appendix*)	One protein with MW ~160 kDa	Danis et al., 2000

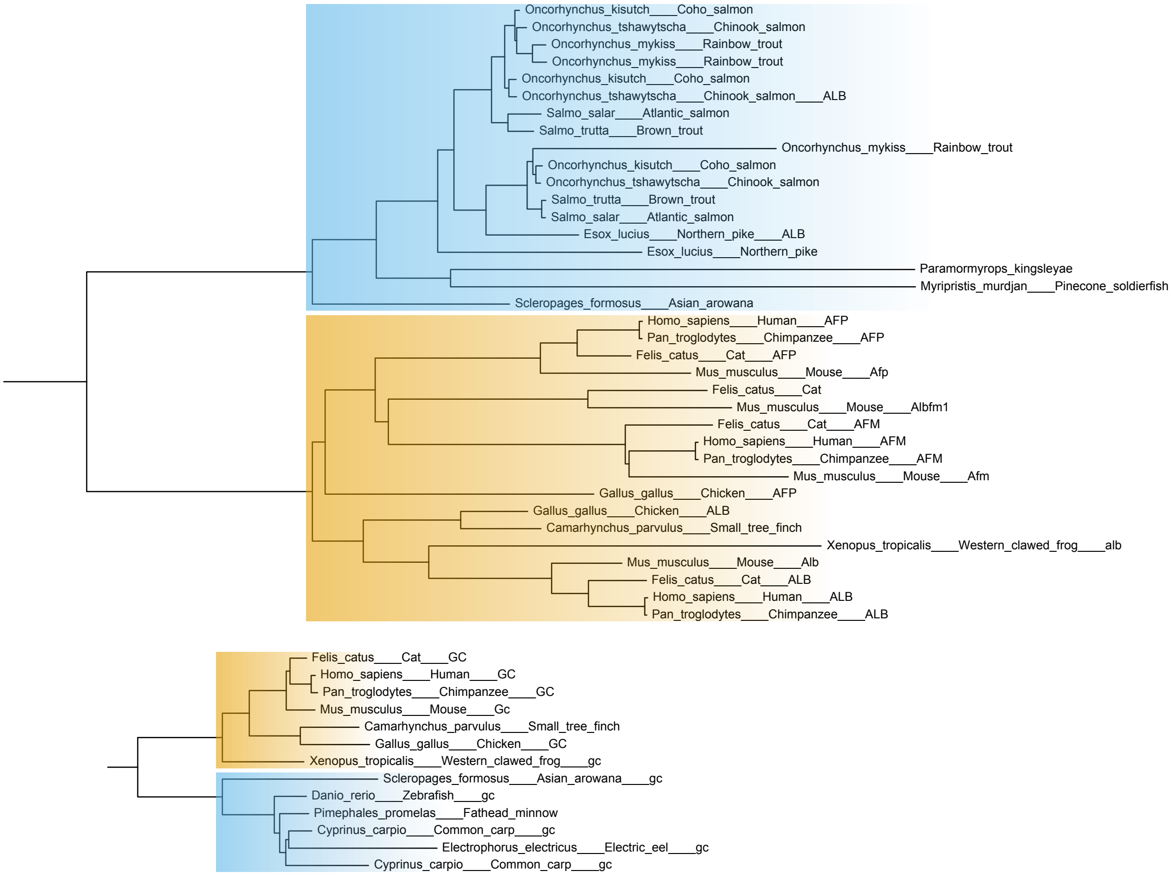

SI Figure 10 *Gene trees of the albuminoids, albumin, AFP and AFM and the GC gene. Tetrapods are highlighted in amber and teleosts in blue. The gene identifiers were used to identify the gene and where possible, a gene symbol was added to the tip label.*

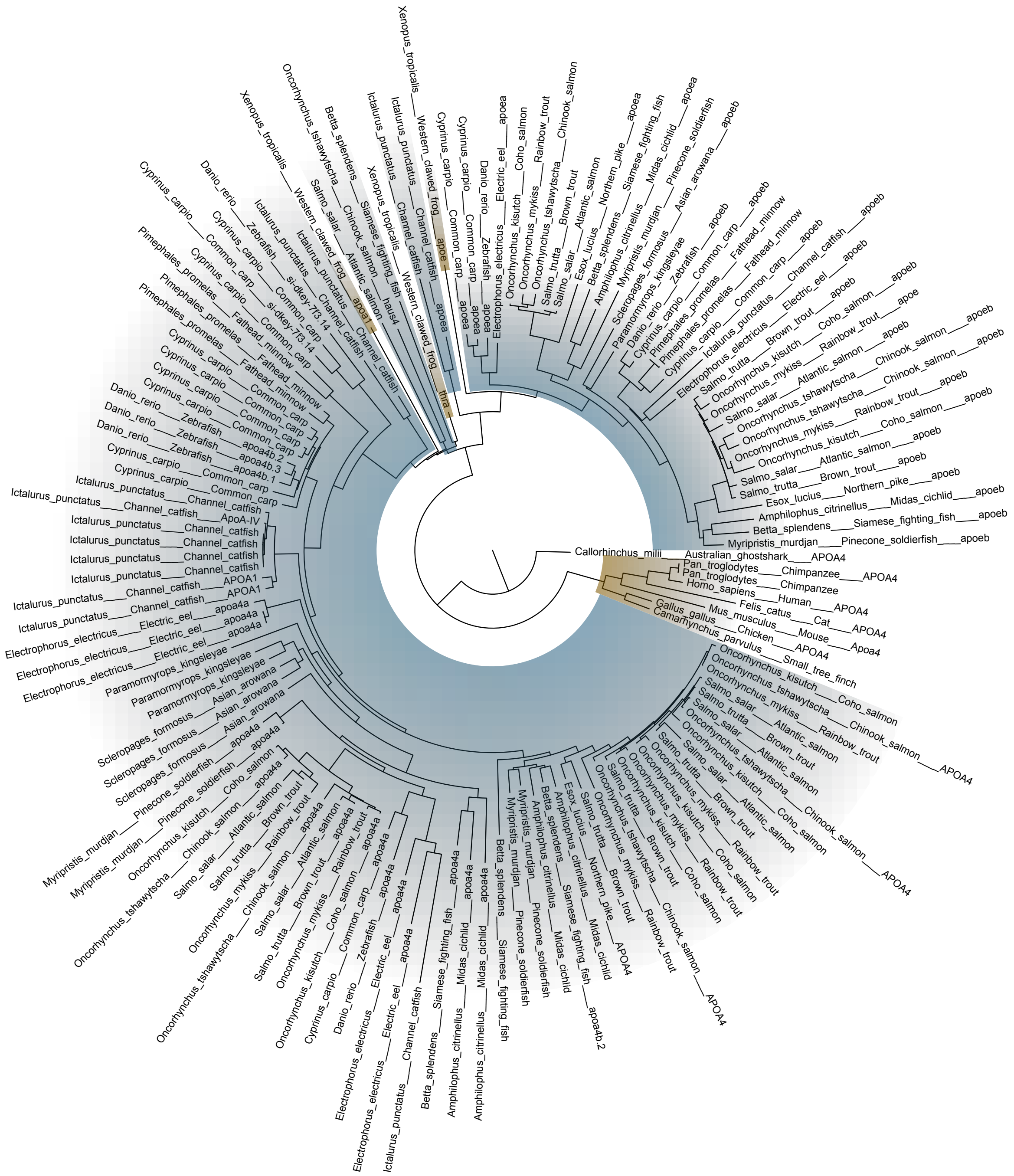

SI Figure 11 *Gene tree of the ApoA1/A4/E orthogroup. Tetrapods are highlighted in amber and teleosts in blue. The gene identifiers were used to identify the gene and where possible, a gene symbol was added to the tip label.*

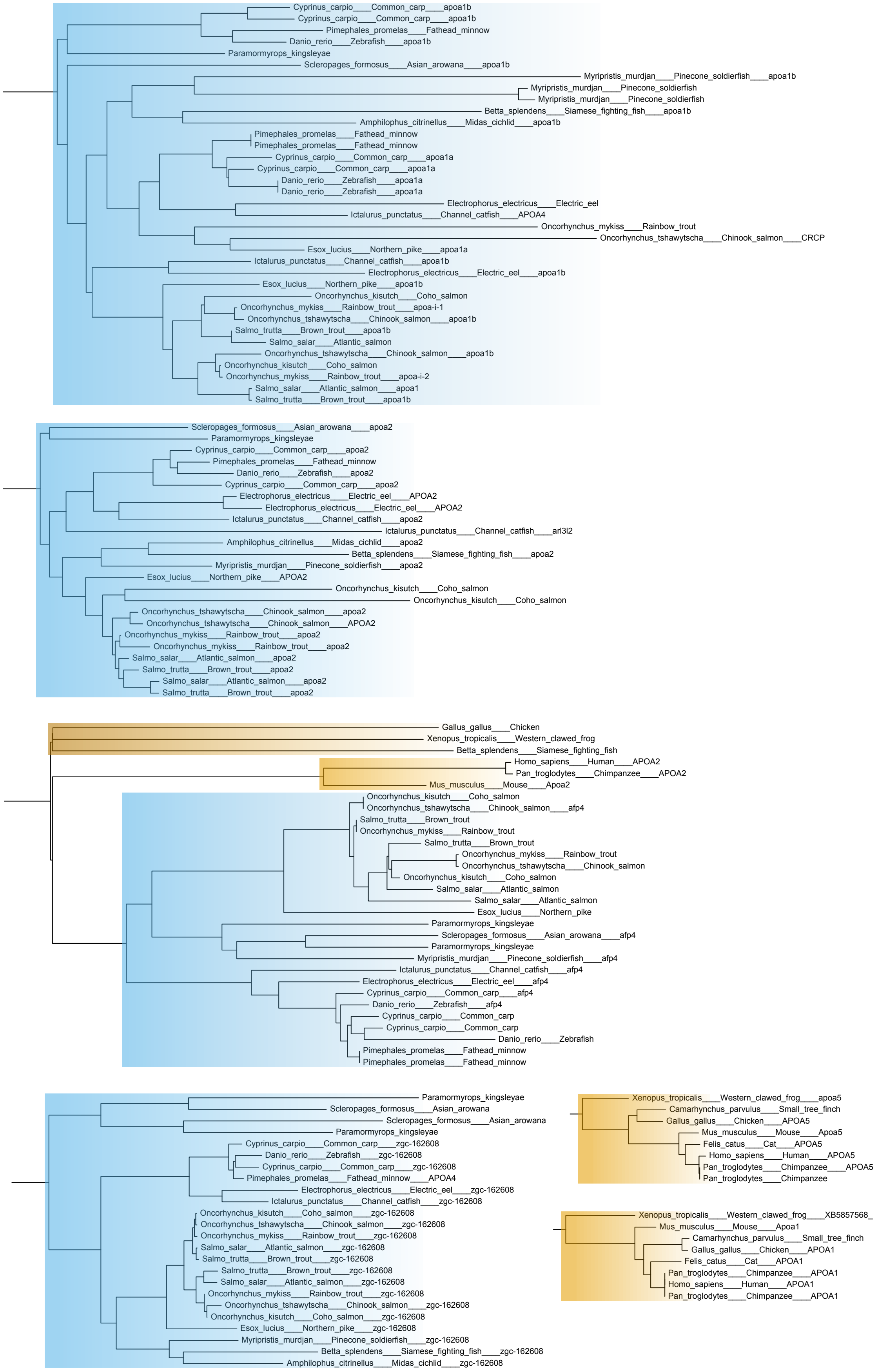

SI Figure 12 *Gene trees of the apolipoprotein A orthogroups. Tetrapods are highlighted in amber and teleosts in blue. The gene identifiers were used to identify the gene and where possible, a gene symbol was added to the tip label.*

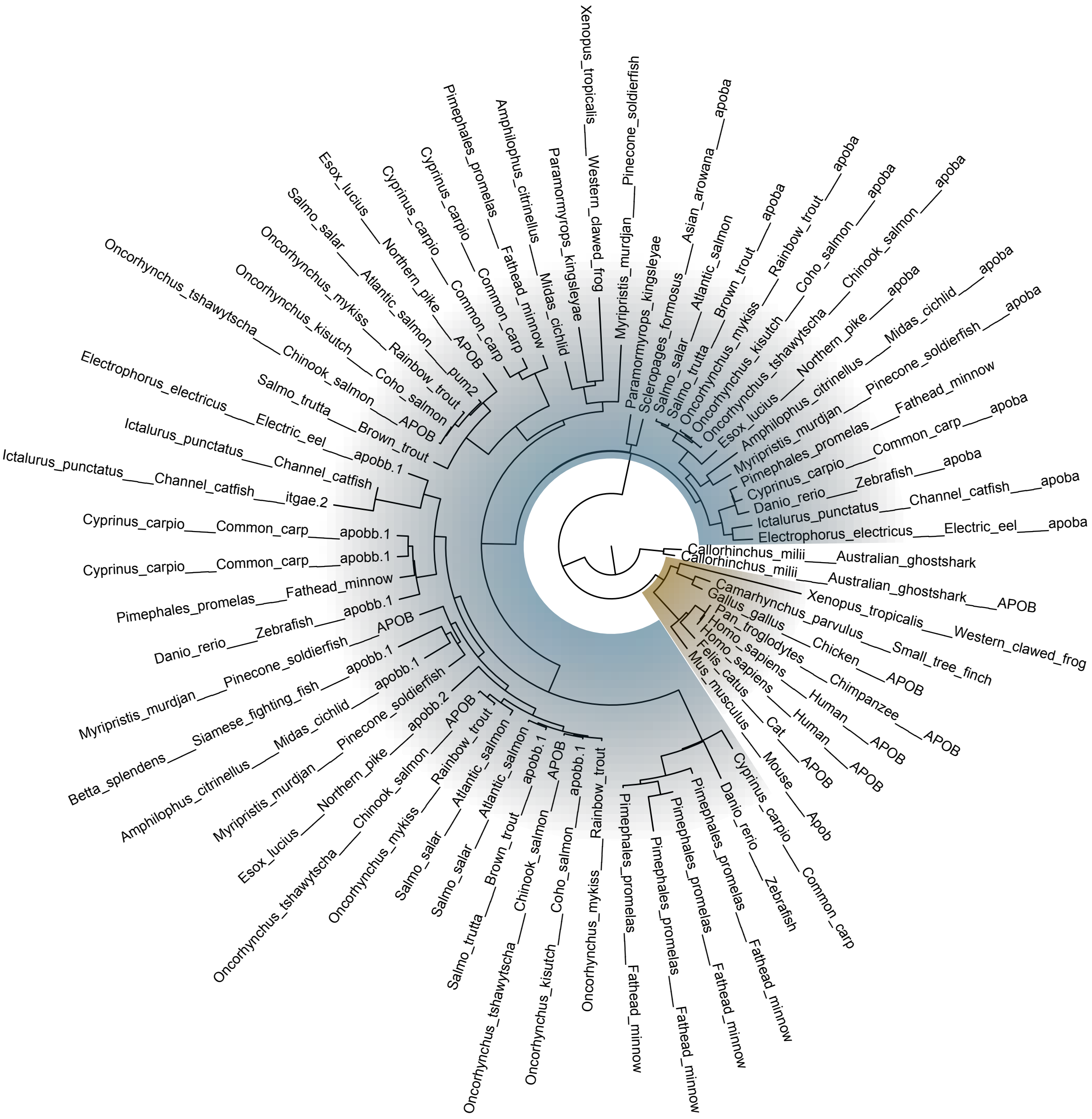

SI Figure 13 *Gene tree of the apolipoprotein B orthogroup. Tetrapods are highlighted in amber and teleosts in blue. The gene identifiers were used to identify the gene and where possible, a gene symbol was added to the tip label.*

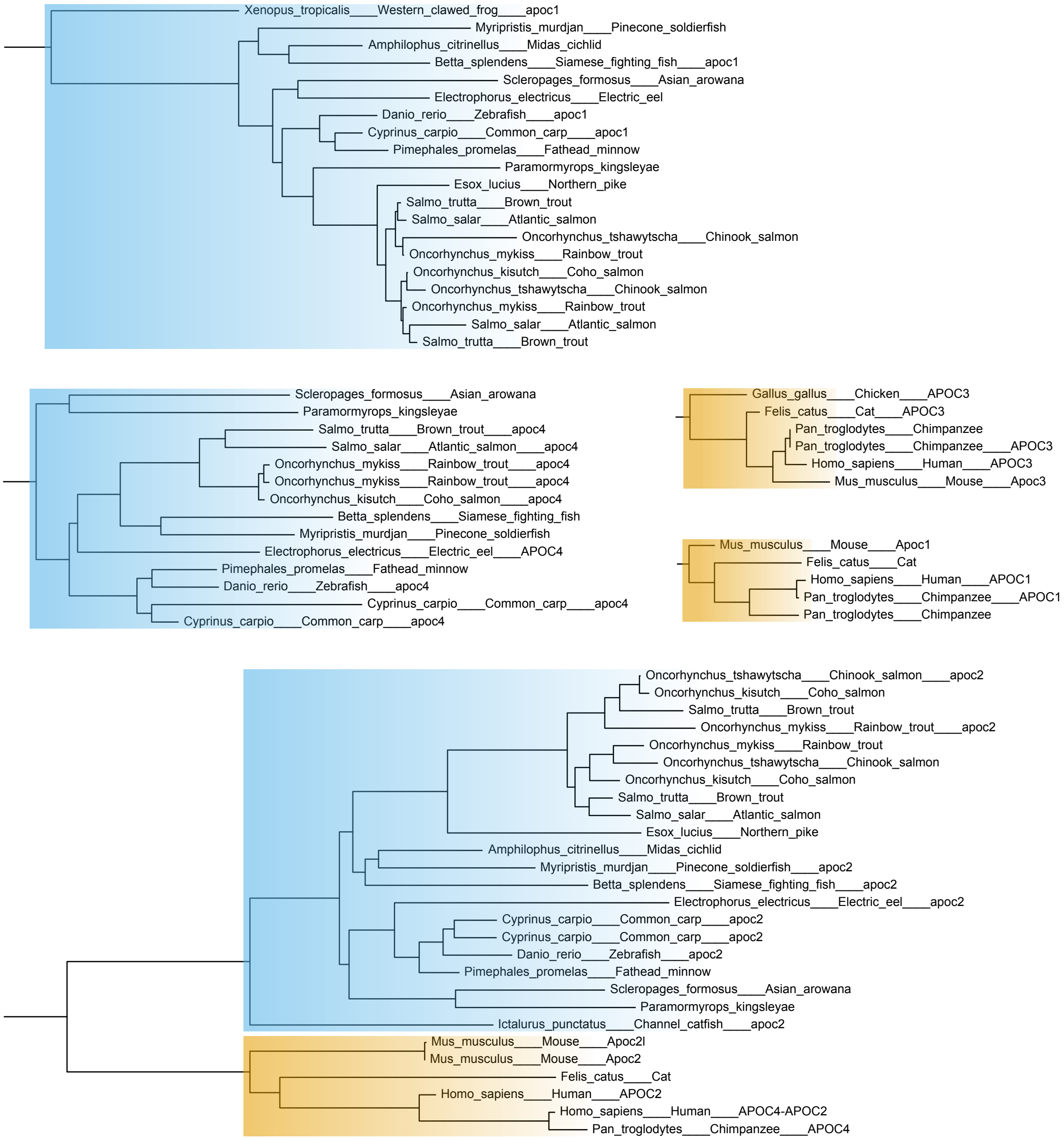

SI Figure 14 *Gene trees of the apolipoprotein C orthogroups. Tetrapods are highlighted in amber and teleosts in blue. The gene identifiers were used to identify the gene and where possible, a gene symbol was added to the tip label.*

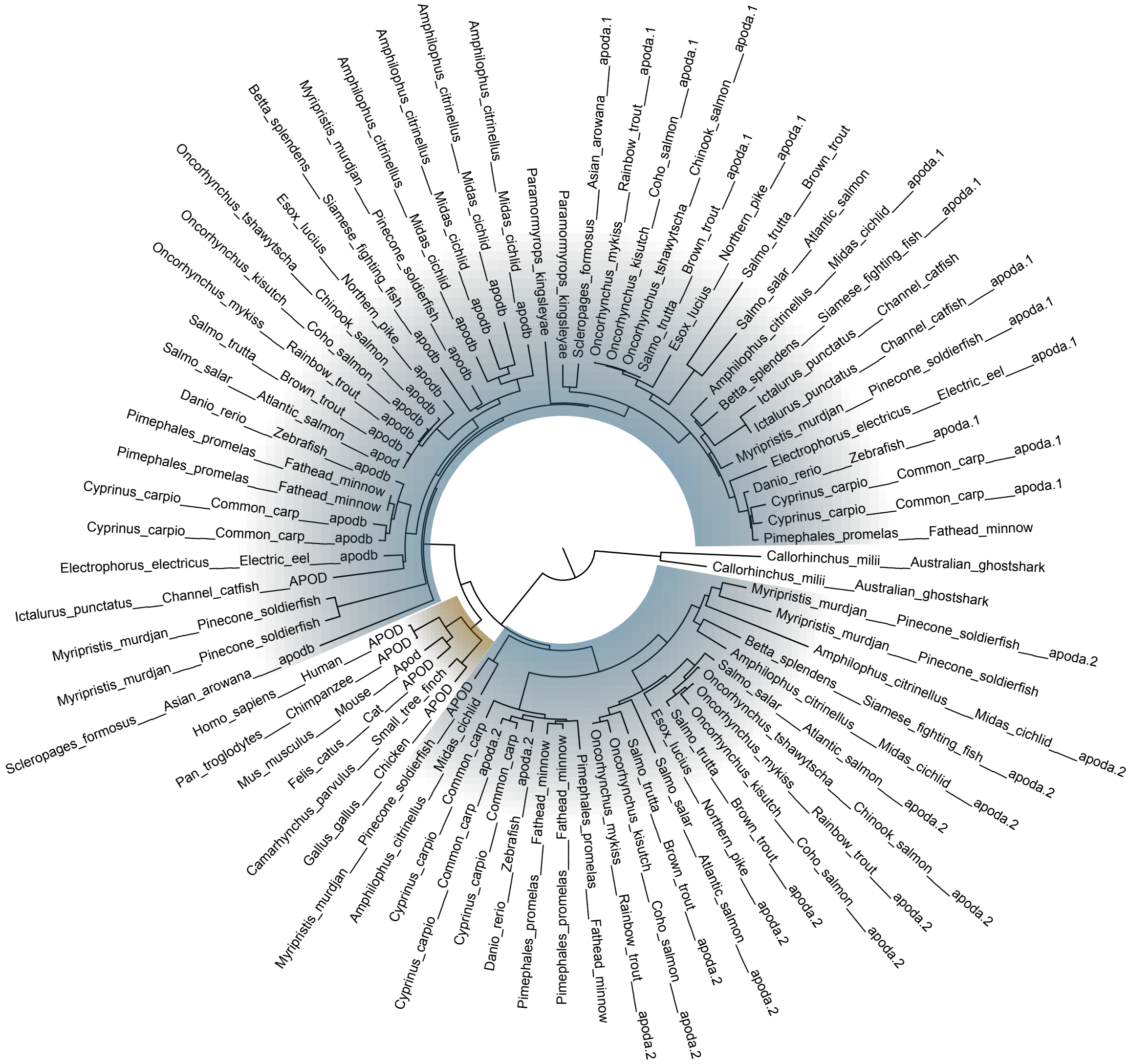

SI Figure 15 *Gene tree of the apolipoprotein D orthogroup. Tetrapods are highlighted in amber and teleosts in blue. The gene identifiers were used to identify the gene and where possible, a gene symbol was added to the tip label.*

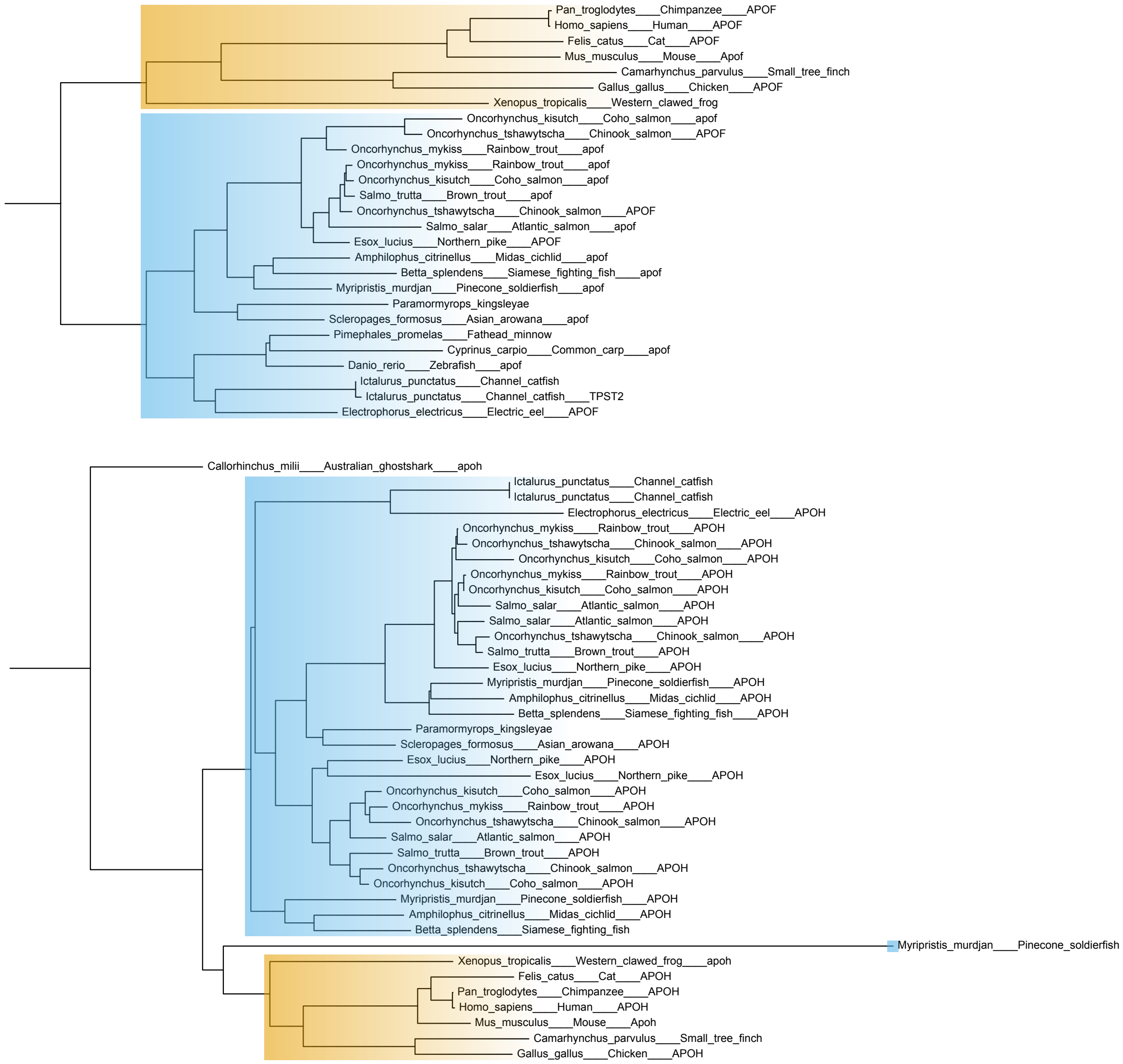

SI Figure 16 *Gene trees of the apolipoprotein F and H orthogroups. Tetrapods are highlighted in amber and teleosts in blue. The gene identifiers were used to identify the gene and where possible, a gene symbol was added to the tip label.*

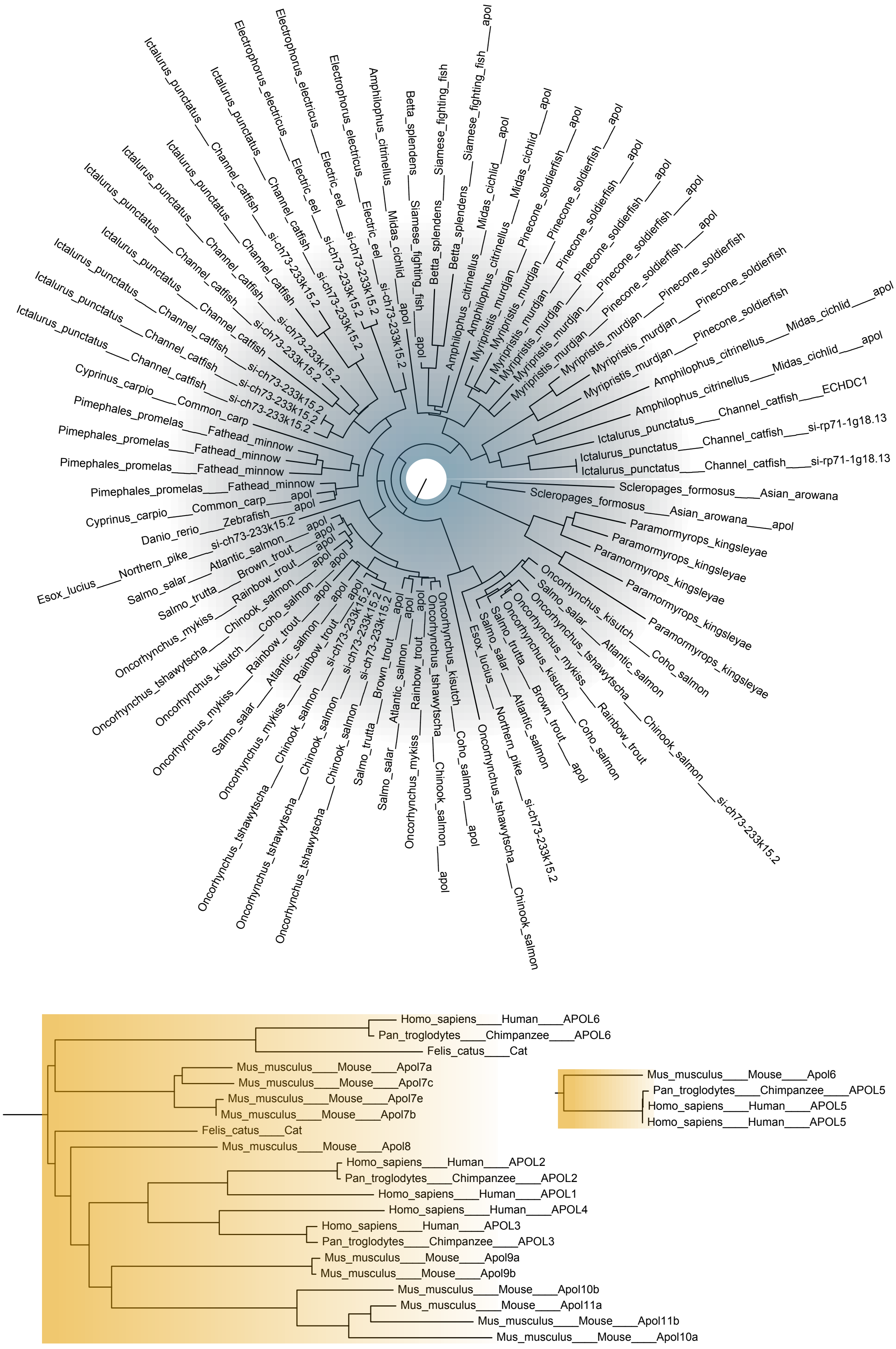

SI Figure 17 *Gene trees of the apolipoprotein L orthogroups. Tetrapods are highlighted in amber and teleosts in blue. The gene identifiers were used to identify the gene and where possible, a gene symbol was added to the tip label.*

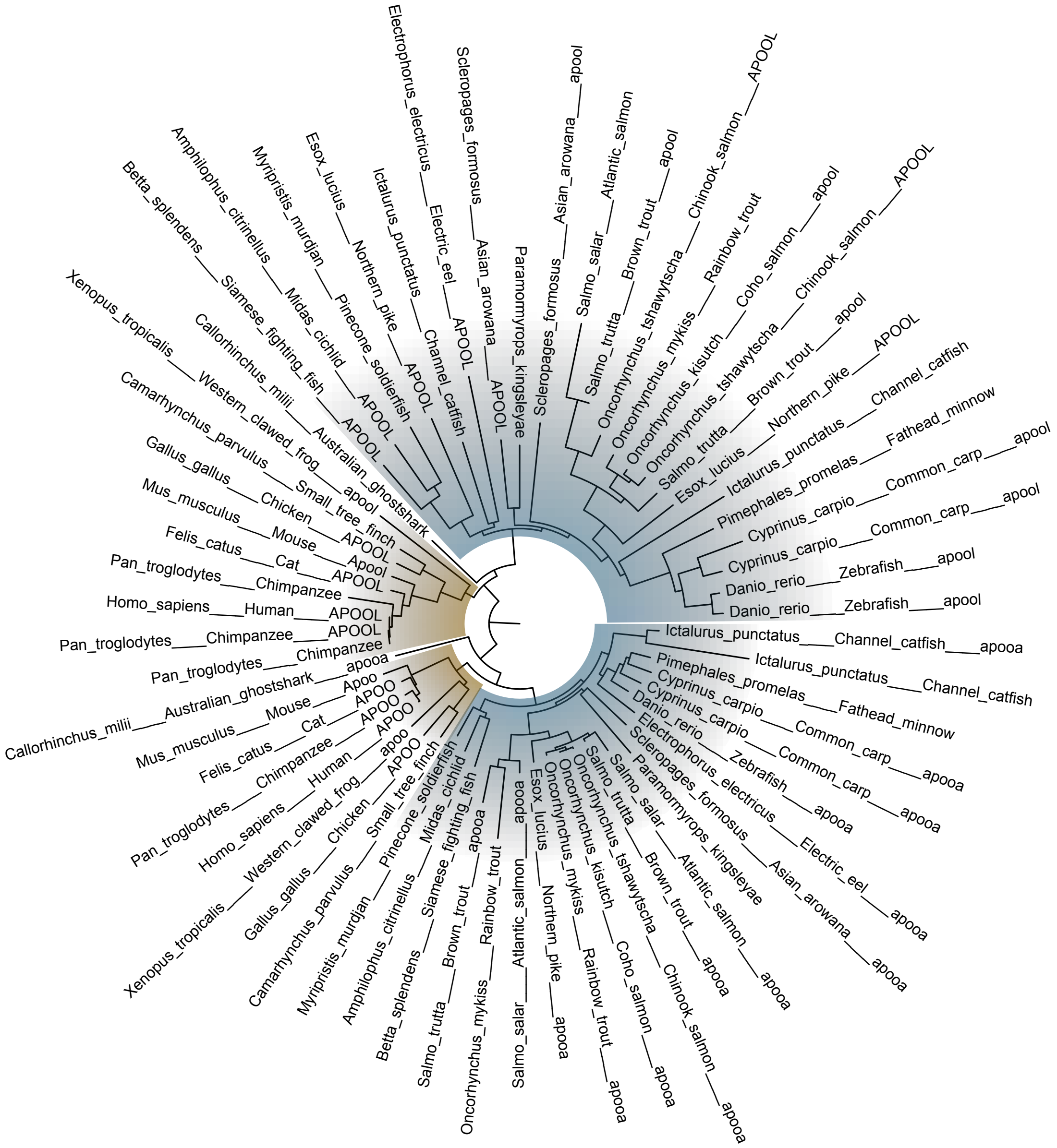

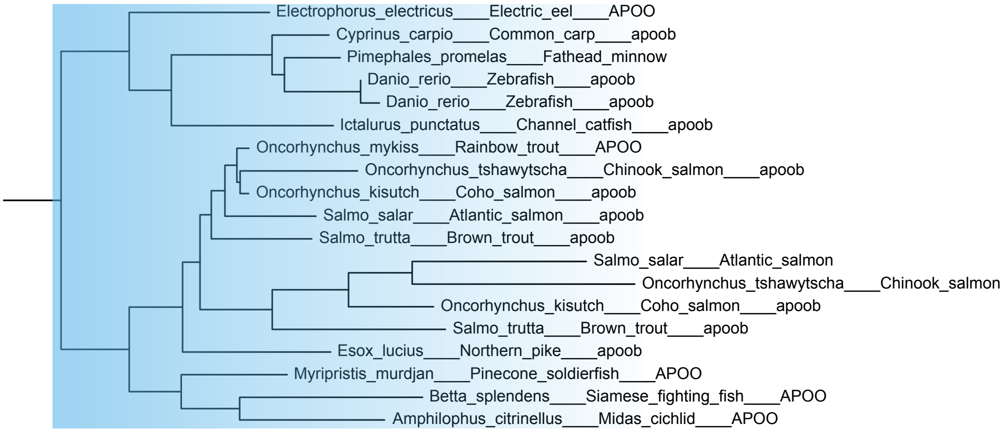

SI Figure 18 *Gene trees of the apolipoprotein O orthogroups. Tetrapods are highlighted in amber and teleosts in blue. The gene identifiers were used to identify the gene and where possible, a gene symbol was added to the tip label.*

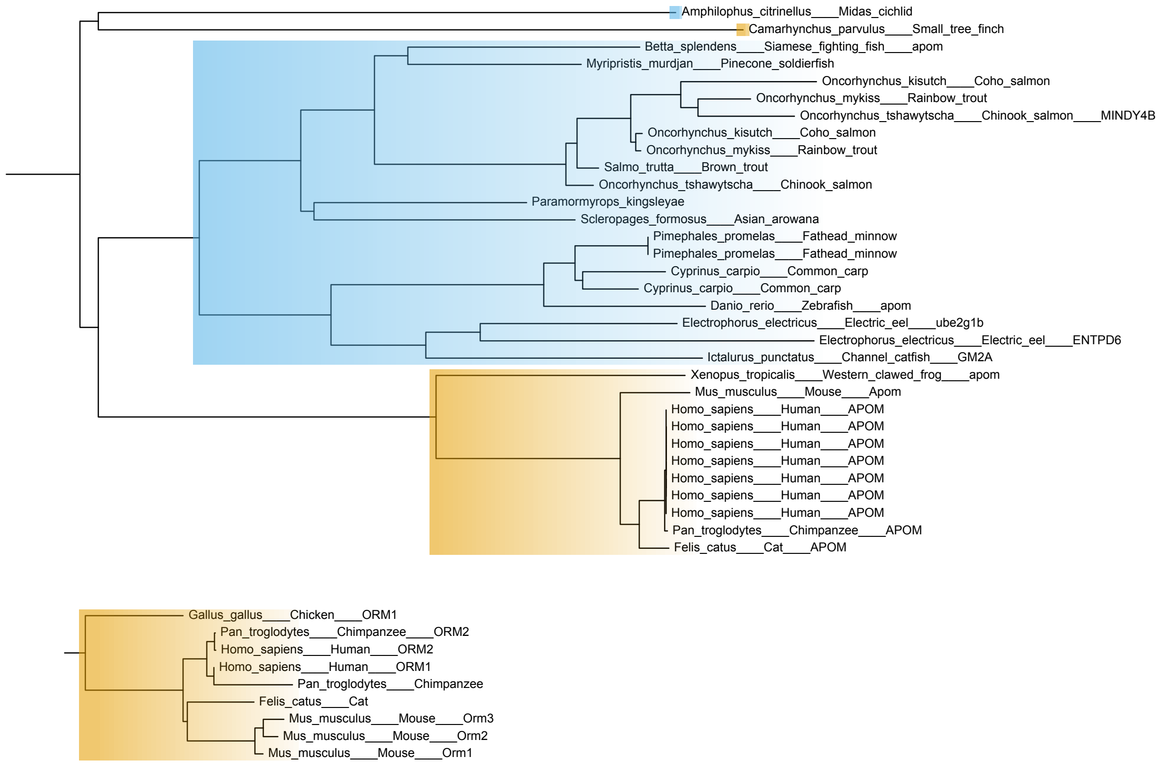

SI Figure 10 *Gene trees of the apolipoprotein M and orosomucoid orthogroups. The tetrapods are highlighted in amber and the teleosts in blue. The gene identifiers were used to identify the gene and where possible, a gene symbol was added to the tip label.*

References (not included in main manuscript)

Alavi FK, Rolf LL, Clarke CR (1993). The pharmacokinetics of sulfachlorpyridazine in channel catfish, Ictalurus punctatus. J Vet Pharmacol Ther. 16(2):232-6. doi: 10.1111/j.1365-2885.1993.tb00168.x.

Amthauer R, Villanueva J, Ines Vera M, Concha M, Krauskopf M (1989). Characterization of the major plasma apolipoproteins of the high-density lipoprotein in the carp (*Cyprinus carpio*). Comparative Biochemistry and Physiology B-Biochemistry & Molecular Biology 92(4): 787-793.

Babin PJ (1987). Plasma-Lipoprotein and Apolipoprotein Distribution as a Function of Density in the Rainbow-Trout (Salmo-Gairdneri). Biochemical Journal 246(2): 425-429.

Babin PJ, Vernier JM (1989). Plasma-lipoproteins in fish. Journal of Lipid Research 30(4): 467-489.

Bakke FK, Monte MM, Stead DA, Causey DR, Douglas A, Macqueen DJ, Dooley H (2020). Plasma Proteome Responses in Salmonid Fish Following Immunization. Front Immunol. 11:581070. doi: 10.3389/fimmu.2020.581070.

Berninger JP, LaLone CA, Villeneuve DL, Ankley GT (2016). Prioritization of pharmaceuticals for potential environmental hazard through leveraging a large-scale mammalian pharmacological dataset. Environ Toxicol Chem. 35(4):1007-20. doi: 10.1002/etc.2965.

Björklund HV, Bylund G (1991). Comparative pharmacokinetics and bioavailability of oxolinic acid and oxytetracycline in rainbow trout (*Oncorhynchus mykiss*). Xenobiotica. 21(11):1511-20. doi: 10.3109/00498259109044401.

Boon JH, Nouws JMF, van der Heijden MHT, Booms GHR, Degen M (1991). Disposition of flumequine in plasma of European eel (Anguilla anguilla) after a single intramuscular injection. Aquaculture 99(3–4): 213-223. https://doi.org/10.1016/0044-8486(91)90242-Y.

Bordbar AK, Saadati Z, Sohrabi N (2004). Analysis of ligand binding process using binding capacity concept. Acta Biochim Pol. 51(4):963-70.

Byrnes L, Gannon F (1990). Atlantic salmon (*Salmo salar*) serum albumin - cDNA sequence, evolution, and tissue expression. DNA and Cell Biology 9(9): 647-655.

Curry S, Mandelkow H, Brick P, Franks N (1998). Crystal structure of human serum albumin complexed with fatty acid reveals an asymmetric distribution of binding sites. Nature Structural and Molecular Biology 5: 827–835. https://doi.org/10.1038/1869.

Danis MH, Filosa MF, Youson JH (2000). An Albumin-Like Protein in the Serum of Non-Parasitic Brook Lamprey (*Lampetra appendix*) Is Restricted to Preadult Phases of the Life Cycle in Contrast to the Parasitic Species *Petromyzon marinus*, Comp. Biochem. Physiol. Biochem. Mol. Biol. 127B: 251–260.

Davidson WS, Birt VL, Birt TP, Green JM (1988). Palmitate-binding, serum albumin-like proteins in salmonids. FEBS Lett. 233(2):299-302. doi: 10.1016/0014-5793(88)80446-0.

Davidson WS, Bartlett SE, Biri TP, Birt VL Green JM (1989). Identification and purification of serum albumin from rainbow trout *(Salmo gairdneri). Comp. Biochem. Physiol.* 93B, 5-9.

Fellows FCI, Hird FJR, Mclean RM, Walker TI (1980). A survey of the non-esterified fatty acids and binding proteins in the plasmas of selected animals. Comp. Biochem. Physiol. 67B:593-597.

Gong, Z.Q., Isolation and characterization of two distinct Rainbow trout albumin genes. 1997, University of Toronto, Toronto, Ontario, Canada.

Gray JE, Doolittle RF (1992). Characterization, primary structure, and evolution of lamprey plasma albumin. Protein Sci. 1(2):289-302. doi: 10.1002/pro.5560010211.

Hayton WL (1997). Whole body autoradiography of rainbow trout exposed to 14C-Acriflavine. Final FDA Report. Contract 223-93-7014 Study 728363-007.

Hunn JB, Greer IE (1990). Colorimetric and refractometer estimates of total plasma protein in striped bass, Morone saxatilis (Walbaum). J. Fish Biol. 36:617-618

Jarboe H, Toth BR, Shoemaker KE, Greenlees KJ, Kleinow KM (1993). Pharmacokinetics, bioavailability, plasma protein binding and disposition of nalidixic acid in rainbow trout (Oncorhynchus mykiss). Xenobiotica. 23(9):961-72. doi: 10.3109/00498259309057035.

Kazuaki Uno, Takahiko Aoki, Ryuji Ueno, Iwao Maeda (1997). Pharmacokinetics and metabolism of sulphamonomethoxine in rainbow trout (*Oncorhynchus mykiss*) and yellowtail (*Seriola quinqueradiata*) following bolus intravascular administration. Aquaculture 153(1–2): 1-8. https://doi.org/10.1016/S0044-8486(97)00012-4.

Kleinow KM, Lech JJ (1988). A review of the pharmacokinetics and metabolism of sulfadimethoxine in the rainbow trout (Salmo gairdneri). Vet Hum Toxicol. 30 Suppl 1:26-30.

Kleinow KM, Beilfuss WL, Jarboe HH, Droy BF, Lech JJ (1992). Pharmacokinetics, bioavailability, distribution, and metabolism of sulfadimethoxine in the rainbow trout (*Oncorhynchus mykiss*). Canadian J. Fisheries and Aquatic Sciences 49:1070-1077. http://www.nrcresearchpress.com/doi/abs/10.1139/f92-118.

Li C, Tan XF, Lim TK, Lin Q, Gong Z (2016). Comprehensive and quantitative proteomic analyses of zebrafish plasma reveals conserved protein profiles between genders and between zebrafish and human. Scientific Reports 6:24329. doi: 10.1038/srep24329.

Liu T, Chen JM, Zhang D, Zhang Q, Peng B, Xu L, Tang H (2021). ApoPred: Identification of apolipoproteins and their subfamilies with multifarious features. Front Cell Dev Biol. 8: 621144. doi: 10.3389/fcell.2020.621144.

Machnes Z, Avtalion R, Shirak A, Trombka D, Wides R, Fellous M, Don J (2008). Male-specific protein (MSP): a new gene linked to sexual behavior and aggressiveness of tilapia males. Horm. Behav. 54: 442-449.

Metcalf VJ, Brennan SO, Chambers GK, George PM (1998). The albumin of the brown trout (*Salmo trutta*) is a glycoprotein, Biochim. Biophys. Acta 1386: 90–96.

Metcalf VJ, Brennan SO, George PM (1999). The Antarctic toothfish (*Dissostichus mawsoni*) lacks plasma albumin and utilises high density lipoprotein as its major palmitate binding protein. Comparative Biochemistry and Physiology B-Biochemistry & Molecular Biology 124(2): 147-155.

Metcalf VJ, George PM, Brennan SO(2007). Lungfish albumin is more similar to tetrapod than to teleost albumins: Purification and characterisation of albumin from the Australian lungfish, *Neoceratodus forsteri*. Comparative Biochemistry and Physiology Part B: Biochemistry and Molecular Biology 147(3): 428-437. https://doi.org/10.1016/j.cbpb.2007.02.009.

Michel CM, Squibb KS, O'Connor JM (1990). Pharmacokinetics of sulphadimethoxine in channel catfish (*Ictalurus punctatus*). Xenobiotica. 20(12):1299-309. doi: 10.3109/00498259009046628.

Mulcahy MF (1969). Blood values in the pike *Esox lucius* L. Journal of Fish Biology 2(3):203-209.

Nakagawa H, Kayama M, Asakawa S (1976). Biochemical Studies on Carp Plasma-Protein .1. Isolation and Nature of an Albumin. Bulletin of the Japanese Society of Scientific Fisheries, 1976. 42(6): 677-685.

Nakamura O, Nozawa Y, Saito E, Ikeda D, Tsutsui S (2009). An alpha-1-acid glycoprotein-like protein as a major component of the ovarian cavity fluid of viviparous fish, *Neoditrema ransonnetii* (Perciformes, Embiotocidae). Comp. Biochem. Physiol. A Mol. Integr. Physiol., 153: 222-229.

Nassef M, Kato-Unoki Y, Furuta T, Nakayama K, Satone H, Shimasaki Y, Honjo T, Oshima Y (2011). Molecular cloning, sequencing, and gene expression analysis of tributyltin-binding protein type 1 in Japanese medaka fish, *Oryzias latipes*. Zool. Sci., 28: 281-285.

Noel ES, Dos Reis M, Arain Z, Ober EA (2010). Analysis of the Albumin/alpha-Fetoprotein/Afamin/Group specific component gene family in the context of zebrafish liver differentiation. Gene Expression Patterns 10(6): 237-243.

Nouws JF, Grondel JL, Schutte AR, Laurensen J (1988). Pharmacokinetics of ciprofloxacin in carp, African catfish and rainbow trout. Vet Q. 10(3):211-6. doi: 10.1080/01652176.1988.9694173.

Oba Y, Shimasaki Y, Oshima Y, Satone H, Kitano T, Nakao M, Kawabata S-I, Honjo T (2007). Purification and characterization of tributyltin-binding protein type 2 from plasma of Japanese flounder, *Paralichthys olivaceus*. J. Biochem. 142: 229-238.

Padrós J, Pelletier É (2000). *In vivo* formation of (+)-anti-benzo[a]pyrene diol-epoxide–plasma albumin adducts in fish. Marine Environmental Research 50(1–5): 347-351. https://doi.org/10.1016/S0141-1136(00)00064-7.

Plakas SM, McPhearson RM, Guarino AM (1988). Disposition and bioavailability of 3H-tetracycline in the channel catfish (*Ictalurus punctatus*). Xenobiotica 18(1):83-93. doi: 10.3109/00498258809055139.

Plakas SM, James MO (1990). Bioavailability, metabolism, and renal excretion of benzoic acid in the channel catfish (Ictalurus punctatus). Drug Metab Dispos. 18(5):552-6.

Plakas SM, Loveland PM, Bailey GS, Blazer VS, Wilson GL (1991). Tissue disposition and excretion of 14C-labelled aflatoxin B1 after oral administration in channel catfish. Food Chem Toxicol. 29(12):805-8. doi: 10.1016/0278-6915(91)90106-h.

Plakas SM, el Said KR, Stehly GR (1994). Furazolidone disposition after intravascular and oral dosing in the channel catfish. Xenobiotica. 24(11):1095-105. doi: 10.3109/00498259409038669.

Plakas SM, El Said KR, Musser SM (2000). Pharmacokinetics, tissue distribution, and metabolism of flumequine in channel catfish (*Ictalurus punctatus*). Aquaculture 187(1–2):1-14. https://doi.org/10.1016/S0044-8486(00)00303-3.

Ponganis KV, Stanski DR (1985). Factors affecting the measurement of lidocaine protein binding by equilibrium dialysis in human serum. J Pharm Sci. 74:57–60. https://doi.org/10.1002/jps.2600740115.

Sandnes K, Lie O, Waagbø R (1988). Normal ranges of some blood chemistry parameters in adult farmed Atlantic salmon, *Salmo salar*. J. Fish Biol. 32:129-136.

Shimasaki Y, Oshima Y, Yokota Y, Kitano T, Nakao M, Kawabata S-I, Imada N, Honjo T (2002). Purification and identification of a tributyltin-binding protein from serum of Japanese flounder, *Paralichthys olivaceus*. Environ. Toxicol. Chem., 21: 1229-1235.

Squibb KS, Michel CM, Zelikoff JT, O'Connor JM (1988). Sulfadimethoxine pharmacokinetics and metabolism in the channel catfish (*Ictalurus punctatus*). Vet Hum Toxicol. 30 Suppl 1:31-5.

Ueno R, Tatsuno T (2003). Pharmacokinetics, Bioavailability, and Protein Binding of Oxolinic Acid in Rainbow Trout. ITE Letters on Batteries, New Technology & Medicine 4, no. 4.

Uno K (1996). Pharmacokinetic study of oxytetracycline in healthy and vibriosis-infected ayu (*Plecoglossus altivelis*). Aquaculture 143(1): 33-42. https://doi.org/10.1016/0044-8486(95)01225-7.

Van Dijk W, Do Carmo S, Rassart E, Dahlbäck B, Sodetz JM (2006). The Plasma Lipocalins α1-Acid Glycoprotein, Apolipoprotein D, Apolipoprotein M and Complement Protein C8γ. In Eds: Akerström B, Borregaard N, Flower DR (2006) Lipocalins. Publ. CRC Press Pages 27. eBook ISBN9780429089886

Wong AKL, Hsia JC (1983). In vitro binding of propranolol and progesterone to native and desialyated human orosomucoid. Can J Biochem Cell Biol. 61:1114–1116. https://doi.org/10.1139/o83-142.

Yildiz M (2024). Computational Analysis of Interactions Between Drugs and Human Serum Albumin. Journal of Molecular Recognition 37(6): e3105. https://doi.org/10.1002/jmr.3105.
